# Shared genetic regulatory networks for cardiovascular disease and type 2 diabetes in multiple populations of diverse ethnicities in the United States

**DOI:** 10.1371/journal.pgen.1007040

**Published:** 2017-09-28

**Authors:** Le Shu, Kei Hang K. Chan, Guanglin Zhang, Tianxiao Huan, Zeyneb Kurt, Yuqi Zhao, Veronica Codoni, David-Alexandre Trégouët, Cardiogenics Consortium, Jun Yang, James G. Wilson, Xi Luo, Daniel Levy, Aldons J. Lusis, Simin Liu, Xia Yang

**Affiliations:** 1 Department of Integrative Biology and Physiology, University of California, Los Angeles, Los Angeles, CA, United States of America; 2 Departments of Epidemiology and Medicine and Center for Global Cardiometabolic Health, Brown University, Providence, RI, United States of America; 3 Hong Kong Institute of Diabetes and Obesity, Department of Medicine and Therapeutics, The Chinese University of Hong Kong, Hong Kong SAR, China; 4 The Framingham Heart Study, Framingham, MA, USA and the Population Sciences Branch, National Heart, Lung, and Blood Institute, Bethesda, MD, United States of America; 5 Sorbonne Universités, UPMC Univ. Paris 06, INSERM, UMR_S 1166, Team Genomics & Pathophysiology of Cardiovascular Diseases, Paris, France; 6 ICAN Institute for Cardiometabolism and Nutrition, Paris, France; 7 Department of Public Health, Hangzhou Normal University School of Medicine, Hangzhou, China; 8 Collaborative Innovation Center for the Diagnosis and Treatment of Infectious Diseases, The First Affiliated Hospital, Zhejiang University, Hangzhou, China; 9 Department of Physiology and Biophysics, University of Mississippi Medical Center, Jackson, MS, United States of America; 10 Department of Biostatistics, Brown University, Providence, RI, United States of America; 11 Departments of Medicine, Human Genetics, and Microbiology, Immunology, and Molecular Genetics, David Geffen School of Medicine, University of California, Los Angeles, Los Angeles, CA, United States of America; 12 Department of Endocrinology, Guangdong General Hospital/Guangdong Academy of Medical Sciences, Guangzhou, Guangdong, China; 13 Institute for Quantitative and Computational Biosciences, University of California, Los Angeles, Los Angeles, CA, United States of America; 14 Molecular Biology Institute, University of California, Los Angeles, Los Angeles, CA, United States of America; New York Genome Center & Columbia University, UNITED STATES

## Abstract

Cardiovascular diseases (CVD) and type 2 diabetes (T2D) are closely interrelated complex diseases likely sharing overlapping pathogenesis driven by aberrant activities in gene networks. However, the molecular circuitries underlying the pathogenic commonalities remain poorly understood. We sought to identify the shared gene networks and their key intervening drivers for both CVD and T2D by conducting a comprehensive integrative analysis driven by five multi-ethnic genome-wide association studies (GWAS) for CVD and T2D, expression quantitative trait loci (eQTLs), ENCODE, and tissue-specific gene network models (both co-expression and graphical models) from CVD and T2D relevant tissues. We identified pathways regulating the metabolism of lipids, glucose, and branched-chain amino acids, along with those governing oxidation, extracellular matrix, immune response, and neuronal system as shared pathogenic processes for both diseases. Further, we uncovered 15 key drivers including *HMGCR*, *CAV1*, *IGF1* and *PCOLCE*, whose network neighbors collectively account for approximately 35% of known GWAS hits for CVD and 22% for T2D. Finally, we cross-validated the regulatory role of the top key drivers using *in vitro* siRNA knockdown, *in vivo* gene knockout, and two Hybrid Mouse Diversity Panels each comprised of >100 strains. Findings from this in-depth assessment of genetic and functional data from multiple human cohorts provide strong support that common sets of tissue-specific molecular networks drive the pathogenesis of both CVD and T2D across ethnicities and help prioritize new therapeutic avenues for both CVD and T2D.

## Author summary

Cardiovascular disease (CVD) and type 2 diabetes (T2D) are two tightly interrelated diseases that are leading epidemics and causes of deaths around the world. Elucidating the mechanistic connections between the two diseases will offer critical insights for the development of novel therapeutic avenues to target both simultaneously. Because of the challenging complexity of CVD and T2D, involving numerous risk factors, multiple tissues, and multidimensional molecular alterations, few have attempted such an investigation. We herein report a comprehensive and in-depth data-driven assessment of the shared mechanisms between CVD and T2D by integrating genomics data from diverse human populations including African Americans, Caucasian Americans, and Hispanic Americans with tissue-specific functional genomics information. We identified shared pathways and gene networks informed by CVD and T2D genetic risks across populations, confirming the importance of well-established processes, as well as unraveling previously under-appreciated processes such as extracellular matrix, branched-chain amino acid metabolism, and neuronal system for both diseases. Further incorporation of tissue-specific regulatory networks pinpointed potential key regulators that orchestrate the biological processes shared between the two diseases, which were cross-validated using cell culture and mouse models. This study suggests potential new therapeutic targets that warrant further investigation for both CVD and T2D.

## Introduction

Cardiovascular disease (CVD) and type 2 diabetes (T2D) are two leading causes of death in the United States [1]. Patients with T2D are at two to six times higher risk of developing CVD compared to those without T2D [2], indicating the importance of targeting common pathogenic pathways to improve the prevention, diagnosis, and treatment for these two diseases. While decades of work has revealed dyslipidemia, dysglycemia, inflammation, and hemodynamic disturbances as common pathophysiological intermediates for both CVD and T2D [3–5], very few studies have directly investigated the genomic architectures shared by the two diseases. While genetic factors are known to play a fundamental role in the pathogenesis of both CVD and T2D [6], a direct comparison of the top risk variants between these diseases has revealed few overlapping loci in genome-wide association studies (GWAS) from multiple large consortia. Aside from the speculation that the strongest genetic risks may be disease-specific, the agnostic approach requiring the application of strict statistical adjustment for multiple comparisons also increases false negative rate because of the lack of “genome-wide significance”.

**Fig 1:**
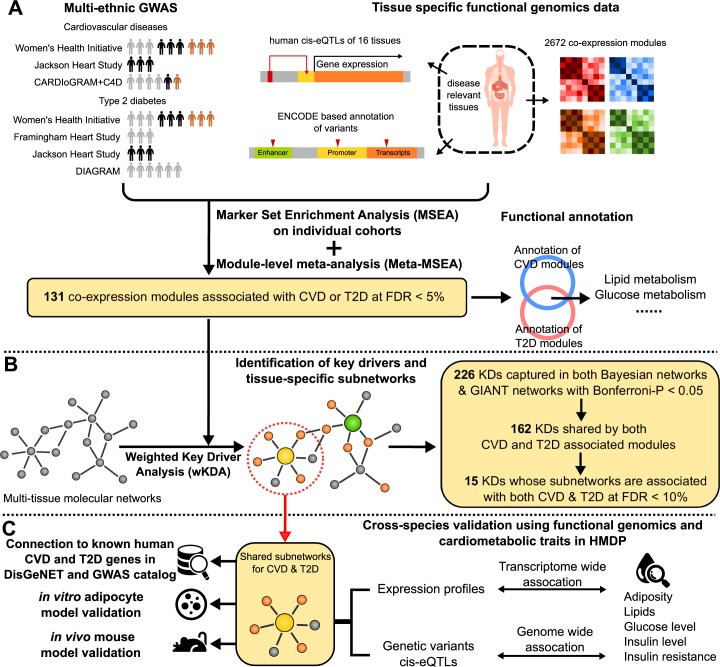
Framework of network-driven integrative genomics analyses. (A) Integration of genetics and functional genomics datasets to identify CVD and T2D associated co-expression modules. The GWAS studies for CVD and T2D were derived from three independent cohorts representing three ethnic populations: WHI (AA, EA, HA), FHS (EA), and JHS (AA). These independent datasets were supplemented with GWAS of coronary artery disease from CARDIoGRAMplusC4D and T2D from DIAGRAM to increase power. We also curated a comprehensive list of tissue-specific functional genomics datasets, including 2672 co-expression modules, human eQTLs of various tissues, and ENCODE based variants annotation. The significant modules were identified by MSEA and Meta-MSEA, and then annotated to reveal shared pathways for CVD and T2D. In MSEA, the co-expression modules were used to define data-driven gene sets each containing functionally related genes, tissue-specificity was determined based on the tissue-origins of the human eQTLs, and ethnic specificity was determined based on the ethnicity of each GWAS cohort. (B) Identification of disease key drivers and subnetworks. We utilized multi-tissue graphical networks to capture key drivers for disease associated co-expression modules using wKDA, then prioritized KDs based on consistency and disease relevance of the subnetworks. (C) Validation of the top key drivers and their subnetworks via intersection with known human CVD and T2D genes from DisGeNET and GWAS catalog, in vitro adipocyte siRNA experiments, and cross-validation at both transcriptomic and genomic levels in the hybrid mouse diversity panels (HMDP).

To meet these challenges, we and others have previously shown that hidden disease mechanisms can be unraveled through the assessment of the combined activities of genetic loci with weak to moderate effects on disease susceptibility by integrating GWAS with functional genomics and regulatory gene networks [7–11]. Importantly, such high-level integration approaches are able to overcome substantial heterogeneity between independent datasets and extract robust biological signals across molecular layers, tissue types, and even species [8, 12–14]. This advantage is mainly conferred by the aggregation of genetic signals from individual studies onto a comparable ground–molecular pathways and gene networks, before conducting meta-analysis across studies [14, 15]. In other words, even if the genetic variants and linkage architecture can be different between studies, the biological processes implicated are more reproducible and comparable across studies [16]. In the current investigation, we employed a systematic data-driven approach that leveraged multi-dimensional omics datasets including GWAS, tissue-specific expression quantitative trait loci (eQTLs), ENCODE, and tissue-specific gene networks (**Fig 1**). GWAS datasets were from three well-characterized and high-quality prospective cohorts of African Americans (AA), European Americans (EA), and Hispanic Americans (HA)—the national Women’s Health Initiative (WHI) [8], the Framingham Heart Study (FHS) [17], and the Jackson Heart Study (JHS) [18]. To maximize the reproducibility of our findings across different populations, we also incorporated meta-analyses of CVD and T2D genetics from CARDIoGRAMplusC4D [19] and DIAGRAM [20]. Further, we comprehensively curated functional genomics and gene networks derived from 25 tissue or cell types relevant to CVD and T2D. A streamlined integration of these rich data sources using our Mergeomics pipeline [14, 15] enabled the identification of shared pathways, gene subnetworks, and key regulators for both CVD and T2D across cohorts and ethnicities. Finally, we validated the subnetworks using adipocyte and knockout mouse models, and confirmed their associations with cardiometabolic traits in the Hybrid Mouse Diversity Panel (HMDP) comprised of >100 mouse strains [21–23].

## Results

### Identification of co-expression modules genetically associated with CVD and T2D across cohorts

**Fig 2:**
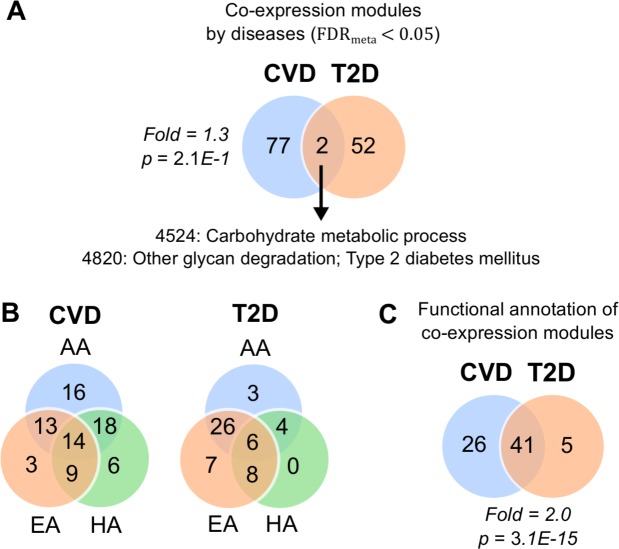
Venn diagrams of overlap in significant co-expression modules and functional categories between diseases and ethnicities. A) Count of module overlaps by disease based on Meta-MSEA; B) Count of module overlaps for each disease by ethnicity based on MSEA of individual studies. Co-expression modules captured in CARDIoGRAMplusC4D and DIAGRAM were not counted due to uncertain ethnic origin; C) Count of independent functional category overlaps by disease based on results from Meta-MSEA in panel A.

We first investigated whether genetic risk variants of CVD and T2D from GWAS of each cohort/ethnicity were aggregated in a functionally coherent manner by integrating GWAS with tissue-specific eQTLs or ENCODE information and gene co-expression networks that define functional units of genes (**Fig 1A**). Briefly, co-expression networks were constructed from an array of transcriptomic datasets of various tissues relevant to CVD and T2D (details in **Methods**). These modules were mainly used to define sets of functionally related genes in a data-driven manner. Genes within the co-expression modules (a module captures functionally related genes) were mapped to single nucleotide polymorphisms (SNPs) that most likely regulate gene functions via tissue-specific eQTLs or ENCODE information. SNPs were filtered by linkage disequilibrium (LD) and then a chi-square like statistic was used to assess whether a co-expression module shows enrichment of potential functional disease SNPs compared to random chance using the marker set enrichment analysis (MSEA) implemented in our Mergeomics pipeline (details in **Methods**) [14]. Subsequently, meta-analyses across individual MSEA results at the co-expression module level were conducted using the Meta-MSEA function in Mergeomics to retrieve robust signals across studies. Among the 2,672 co-expression modules tested, 131 were found to be significant as defined by false discovery rate (FDR) < 5% in Meta-MSEA across studies (**Table 1, S1 Table**). Moreover, the majority of the disease relevant tissues or cell types included in our analysis yielded informative signal, supporting the systemic pathogenic perturbations known for CVD and T2D (**S1 Fig**). Of the significant modules identified, 79 were associated with CVD and 54 with T2D. Two modules were associated with both diseases, with one enriched for “carbohydrate metabolism” genes and the other over-represented with “other glycan degradation; known T2D genes” (**Fig 2A, S1 Table**). Examination of these two shared modules showed that the genetic signals driving the module significance were largely different between CVD and T2D, with 14.8% lead SNPs overlapping for the carbohydrate metabolism module and 5.8% lead SNPs overlapping for the glycan degradation module between diseases. These results indicate that the GWAS signals for the two diseases in each module do not necessarily overlap, but the CVD and T2D genes are likely functionally connected since they are co-expressed in the same modules and annotated with coherent functions. Additionally, the majority of the CVD modules and T2D modules were identified in more than one ethnic group based on MSEA analysis of individual studies, supporting consistency across ethnicities (**Fig 2B)**.

**Table 1:** Summary of top co-expression modules associated with CVD or T2D (FDR < 1% in Meta-MSEA, in column FDR_meta_).

Disease	Module ID	Tissue	Annotation	Gene No.	CAR+C4D/ DIAGRAM	JHS	FHS	WHI	WHI	WHI	P_meta_	FDR_meta_
					Mixed	AA	EA	EA	AA	HA		
**CVD**	4406	O1, O2, O5	NA	154	**3.32E-10**	NS	-	2.83E-02	4.41E-03	NS	5.73E-09	<0.01%
	4522	Adp, Lv, T	Signaling by FGFR mutants	2072	**1.03E-04**	1.62E-02	-	3.80E-02	5.53E-03	2.86E-02	3.39E-08	<0.01%
	4540	O4, O5	NA	1233	**9.72E-04**	NS	-	NS	1.50E-02	5.52E-04	5.07E-07	0.06%
	5242	Adr	Cholesterol Biosynthesis	306	**4.19E-06**	4.71E-02	-	NS	2.31E-02	NS	2.64E-06	0.08%
	4087	Adp, Dg	Carboxylic acid metabolic process	158	**2.34E-06**	NS	-	NS	8.63E-03	2.17E-02	4.24E-06	0.09%
	4019	Ly	Transmembrane transport of small molecules	2876	**1.89E-03**	4.46E-02	-	NS	NS	**6.85E-04**	7.91E-06	0.20%
	4941	O4, O5	Establishment of localization	908	**8.97E-06**	1.52E-02	-	NS	NS	3.94E-02	2.72E-06	0.21%
	5023	Ly	TCA cycle and respiratory electron transport	2890	NS	**6.37E-05**	-	**1.53E-03**	NS	1.50E-02	1.15E-05	0.22%
	blue	O2, O4	Cell cycle	657	1.08E-02	NS	-	NS	NS	**1.77E-04**	3.85E-06	0.30%
	5329	Adr	Biological oxidations	1028	NS	2.32E-02	-	5.01E-03	3.26E-02	2.26E-02	2.21E-05	0.35%
	124	O3, O4	NA	14	NS	**1.48E-03**	-	NS	**7.05E-07**	NS	4.86E-06	0.55%
	4656	O3, O4	Cellular protein complex assembly	371	NS	NS	-	NS	3.64E-03	**2.27E-04**	8.85E-06	0.67%
	4147	O5	NA	111	**1.55E-02**	**2.06E-04**	-	NS	8.85E-03	NS	5.72E-06	0.68%
	4989	Adr	Metabolism of amino acids and derivatives	453	**1.86E-03**	7.41E-03	-	NS	**3.71E-04**	NS	7.81E-05	0.82%
**T2D**	5323	Mn	NA	38	**8.68E-04**	NS	NS	**2.25E-04**	**1.05E-03**	NS	1.58E-07	0.02%
	5250	Adp, Dg, Mn	NA	37	**4.78E-05**	NS	NS	3.01E-02	**3.46E-07**	NS	4.32E-07	0.03%
	4880	Mn	NA	141	8.96E-03	NS	1.18E-02	**5.06E-04**	NS	NS	1.61E-06	0.06%
	6872	Mn	NA	119	NS	**1.26E-03**	7.44E-03	**7.79E-03**	NS	NS	1.26E-06	0.06%
	4879	Ms	NA	376	3.18E-02	NS	**5.88E-04**	NS	**2.66E-03**	**2.20E-03**	1.19E-06	0.14%
	6533	Mn	Cholesterol biosynthesis	48	NS	5.02E-03	NS	NS	NS	**1.26E-06**	1.06E-05	0.25%
	6977	Bld, O3	NA	40	3.66E-02	NS	**4.01E-05**	NS	1.81E-02	4.05E-02	1.71E-06	0.39%
	6675	Mn	Cholesterol biosynthesis	152	**3.72E-03**	3.35E-02	NS	NS	NS	**2.06E-05**	2.56E-05	0.52%
	37	O2	NA	34	**1.94E-03**	5.53E-03	NS	NS	**9.38E-04**	NS	4.95E-06	0.57%
	4302	Adp	NA	40	**2.07E-03**	NS	NS	4.80E-03	**4.05E-06**	NS	9.89E-06	0.71%
	6690	Adr	Complement and coagulation cascades	641	1.93E-02	**1.01E-04**	NS	2.24E-02	NS	NS	1.36E-05	0.86%
	4059	Dg	SLC mediated transmembrane transport	51	NS	3.05E-02	5.80E-03	NS	**1.50E-02**	NS	1.29E-05	0.86%
	4937	Dg	Amino acid metabolic process	80	9.21E-03	NS	5.88E-03	NS	**1.37E-03**	NS	2.11E-05	0.89%
	5059	Ve	TCA cycle and respiratory electron transport	164	**7.31E-04**	NS	2.74E-02	**8.66E-04**	NS	NS	6.64E-06	0.95%

Module IDs were randomly assigned IDs to co-expression modules. The annotation refers to the top functional category enriched in the co-expression modules (Bonferroni-corrected p< 0.05 based on Fisher’s exact test, number of direct overlapping genes > 5). Numbers in scientific format were p-values from MSEA or Meta-MSEA analysis, and those reaching FDR < 20% in individual cohort analysis via MSEA (not the FDR_meta_ in Meta-MSEA) are highlighted in bold. CAR+C4D: CARDIoGRAMplusC4D; Mixed: mixed ethnicities; JHS: Jackson Heart Study; FHS: Framingham Heart Study; WHI: Women’s Health Initiative; AA: African Americans; HA: Hispanic Americans; EA: European Americans; P_meta_ and FDR_meta_: p and FDR values from Meta-MSEA analysis across cohorts. Adp–adipose tissue; Adr—adrenal gland; Bld–Blood; Dg—digestive tract; Lv–liver; Ly–lymphocyte; Mn-monocyte; Ms–muscle; O1 –chromosomal distance mapping based on a 50kb window; O2 –ENCODE-based Regulome SNPs; O3 –combining all tissue-specific eQTLs into a single multi-tissue eSNP set; O4 –merging eQTL sets with Regulome data; O5 –combined mapping (distance, eQTLs, ENCODE); T–thyroid gland; Ve–vascular endothelium.

### Shared biological processes among the CVD/T2D-associated co-expression modules

**Fig 3:**
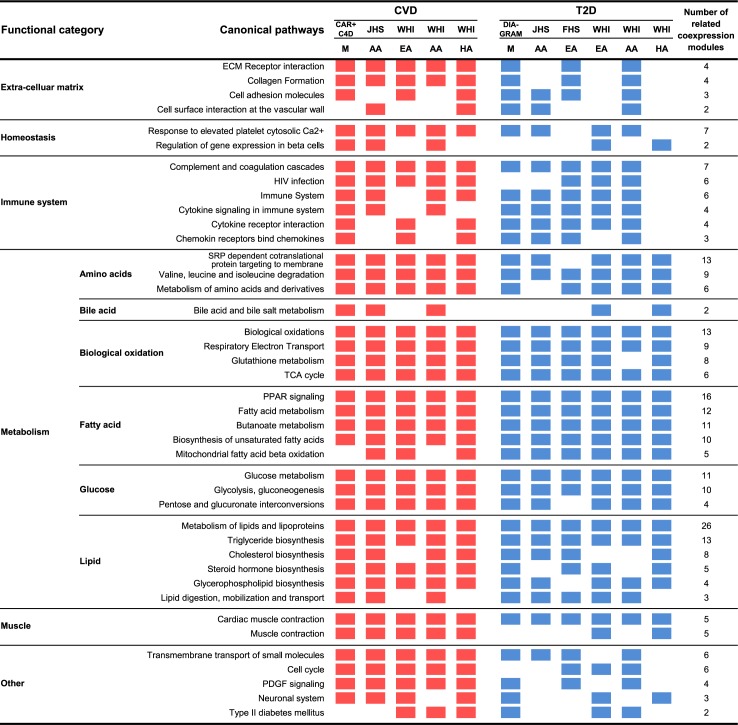
Summary of 41 independent functional categories enriched in both CVD and T2D co-expression modules (Bonferroni-corrected p< 0.05 based on Fisher’s exact test, number of direct overlapping genes > 5). Independent functional categories were defined as the categories with pair-wise overlapping ratio < 10%. Red and blue block indicates that the significant CVD or T2D co-expression modules identified from the study and ethnicity origin are enriched for the particular functional category term. CAR+C4D: CARDIoGRAMplusC4D; M: mixed ethnicities; AA: African Americans; HA: Hispanic Americans; EA: European Americans.

Apart from the two directly overlapping modules, between the CVD- and T2D-associated modules there were many overlapping genes, indicating additional shared functions that contribute to both diseases (**S2 Fig**). Upon annotating the disease-associated modules using functional categories curated in Kyoto Encyclopedia of Genes and Genomes (KEGG) and Reactome while correcting for the overlaps between pathways (method details in **S1 Text**; **S3 Fig**; **S2 Table**), we found significant functional overlaps between the CVD and T2D modules (overlap p = 3.1e-15 by Fisher’s exact test, **Fig 2C**). We further ranked all the enriched functional categories by the number of CVD/T2D modules that were annotated with each functional term (**Fig 3**), which showed a wide spectrum of biological processes shared by both CVD and T2D across ethnicities and cohorts. Of the top ranked processes for the significant co-expression modules identified, we observed well-established pathogenic processes such as lipid and fatty acid metabolism [24], glucose metabolism [25], oxidation [26], and cytokine signaling [27]. Pathways previously implicated mainly for T2D such as beta-cell function were also found to be shared for both CVD and T2D. Interestingly, our completely data-driven approach also identified extracellular matrix (ECM) and branched chain amino acids (BCAA) metabolism as top functional categories whose roles in the development of cardiometabolic disorders have only been implicated in recent experimental work [28–30]. Furthermore, our analysis also revealed under-appreciated processes involving the neuronal system and transport of small molecules.

### Identification and prioritization of key drivers (KDs) and subnetworks for the CVD/T2D-associated modules

The coexpression networks used above mainly served to capture coexpression patterns between genes and to define data-driven gene sets that contain functionally related genes, but they do not carry detailed topology information on gene-gene regulatory relationships. To dissect the gene-gene interactions within and between the 131 disease-associated modules, and to identify key perturbation points shared for both CVD and T2D modules, we used the GIANT networks [31] and Bayesian networks (BNs) from 25 CVD and T2D relevant tissue and cell types, which provide detailed topological information on gene-gene regulatory relationships necessary for the downstream network analysis. The BNs used in our study were generated using similar sets of mouse and human gene expression datasets as used for the co-expression networks, but additionally incorporated genetic data to model causal gene regulatory networks, whereas the GIANT networks were derived based on independent gene expression datasets and protein interaction information. We included both types of gene regulatory networks to increase the coverage of functional connections between genes and only considered KDs identified in both to enhance the robustness of KD prediction.

Specifically, all genes in each of the co-expression modules genetically associated with CVD or T2D as identified in our study were mapped onto the GIANT and BN graphical networks to identify KDs using the weighted key driver analysis (wKDA) implemented in Mergeomics [14], where KDs were defined as genes whose local network neighborhoods demonstrate significant enrichment of genes from disease-associated modules (details in **Methods**; concept depicted in **S4 Fig**). Of note, wKDA gives higher weight to network edges that are consistent across network models constructed from independent studies, therefore alleviating potential bias caused by dataset heterogeneity. We identified 226 KDs that were consistently captured in Bayesian and GIANT network at Bonferroni-corrected p-value < 0.05 **(Fig 1B)**, among which 162 were KDs for both CVD and T2D associated modules. Bonferroni-correction was used here to focus on the strongest KDs for prioritization purposes. To further prioritize these 162 shared KDs, tissue-specific subnetworks of these KDs were evaluated using Meta-MSEA to rank the magnitude of their genetic association with CVD and T2D across cohorts, yielding 15 top-ranked KDs at FDR<10% in Meta-MSEA for CVD and T2D separately (combined FDR<1% for both diseases simultaneously) **(Fig 1B, Table 2)**. The top KD subnetworks were related to similar pathogenic processes highlighted in the previous section, including cholesterol biosynthesis, respiratory electron transport, immune system and ECM. We further inferred the directionality of the effects of each specific KD on both diseases using GWAS signals mapped to each KD based on eQTLs or chromosomal distance (details in **Methods**; results in **S5 Fig**). This analysis differentiated the KDs into those showing consistent direction of association for both CVD and T2D (*ACLY*, *CAV1*, *SPARC*, *COL6A2*, *IGF1*), inverse directions with CVD and T2D (*HMGCR*, *IDI1*), and uncertain directions (**Table 2**). Therefore, the shared KDs do not necessarily affect the risks for the two diseases in the same direction.

**Table 2:** Summary of the 15 key drivers and their corresponding subnetworks shared by CVD and T2D.

Key drivers	Gene name	Sub-net size	Tissues	*P*_*CVD*_	*FDR*_*CVD*_	*P*_*T*2*D*_	*FDR*_*T*2*D*_	No. of CVD module	No. of T2D module	Suggestive genetic effect direction (CVD/T2D)	Subnetwork function
*ACAT2*	Acetyl-CoA Acetyltransferase 2	192	Adp, Dg, Lv, Ms, T	1.24E-03	5.32%	5.37E-03	4.35%	6	7	uncertain	Cell cycle; Cholesterol biosynthesis
*ACLY*	ATP Citrate Lyase	129	Adp, Dg, Lv, Ms	5.96E-04	6.17%	5.78E-05	0.47%	5	6	consistent	Cholesterol biosynthesis; Steroid biosynthesis
*CAV1*	Caveolin 1	954	Adp, Adr, Art, Dg, Ms, T, Ve	1.24E-05	0.20%	3.96E-05	0.32%	7	4	consistent	Immune system; Focal adhesion
*COL6A2*	Collagen Type VI Alpha 2 Chain	294	Adp, Adr, Dg, Ms, T	2.47E-03	4.45%	4.97E-05	0.40%	2	1	consistent	Extracellular matrix
*COX7A2*	Cytochrome C Oxidase Subunit 7A2	152	Adp, Adr, Art, Bld, Dg, Lv, Ly	2.34E-04	3.79%	1.31E-04	1.85%	1	4	uncertain	Respiratory electron transport
*DBI*	Diazepam Binding Inhibitor	181	Adp, Art, Bld, Dg, Is, Lv, Ly, Ms	1.57E-03	7.70%	1.33E-02	6.75%	5	5	uncertain	Respiratory electron transport
*HMGCR*	3-Hydroxy-3-Methylglutaryl-CoA Reductase	75	Art, Dg, Lv, Ms	7.53E-03	9.09%	7.28E-03	4.87%	1	5	opposite	Cholesterol biosynthesis; Steroid biosynthesis
*IDI1*	isopentenyl-diphosphate delta isomerase 1	89	Adp, Art, Dg, Is, Lv, Ms, T	6.77E-03	8.95%	2.13E-03	3.46%	3	4	opposite	Cholesterol biosynthesis; Steroid biosynthesis
*IGF1*	insulin like growth factor 1	993	Adr, Ms	2.65E-03	5.37%	3.71E-04	1.20%	7	2	consistent	Immune system; Focal adhesion
*MCAM*	melanoma cell adhesion molecule	183	Adp, Adr, Art, Ms, T	2.65E-03	7.16%	1.93E-03	5.22%	4	2	uncertain	Extracellular matrix
*MEST*	mesoderm specific transcript	132	Adp, Adr, Lv, Ms	1.66E-03	3.36%	6.84E-04	1.58%	4	2	uncertain	Fibroblast growth factor signaling
*MSMO1*	methylsterol monooxygenase 1	133	Adp, Art, Dg, Lv, Ms, T,	2.38E-03	7.70%	4.34E-05	0.63%	1	4	uncertain	Cholesterol biosynthesis; Steroid biosynthesis
*PCOLCE*	procollagen C-endopeptidase enhancer	307	Adp, Adr, Art, Hy, Lv, Ms	1.14E-03	6.17%	1.71E-06	0.03%	2	2	uncertain	Extracellular matrix
*SPARC*	secreted protein acidic and cysteine rich	482	Adp, Adr, Art, Dg, Lv, Ms, Ve	1.81E-03	9.63%	2.02E-03	8.18%	5	3	consistent	Extracellular matrix
*ZFP36*	ZFP36 ring finger protein	176	Adp, Adr, Art, Lv, Ly, Ms	1.42E-03	8.45%	1.64E-02	7.69%	3	3	uncertain	Hypoxia-inducible factors; CD40 signaling

P and FDR values were based on Meta-MSEA analysis of the KD subnetworks for enrichment of CVD or T2D GWAS signals across cohorts. The subnetwork size indicates the number of neighboring genes directly connected to a KD when all the tissue-specific networks where the KD was found are combined. No. of module columns indicate the number of CVD or T2D–associated co-expression modules from which each KD was identified. Suggestive genetic effect direction was designated “consistent” or “opposite” if the proportion of variants having consistent or opposite effect direction in CVD or T2D was statistically significant in either eQTL mapping or chromosomal distance mapping. Otherwise, “uncertain” was called. Subnetwork function was annotated based on KEGG and Reactome databases. Adp–adipose tissue; Adr—adrenal gland; Art–artery; Dg—digestive tract; Is–Islet; Hy–hypothalamus; Lv–liver; Ly–lymphocyte; Ms–muscle; T: thyroid gland; Ve: vascular endothelium.

### Shared KDs and subnetworks orchestrate known CVD and T2D genes

**Fig 4:**
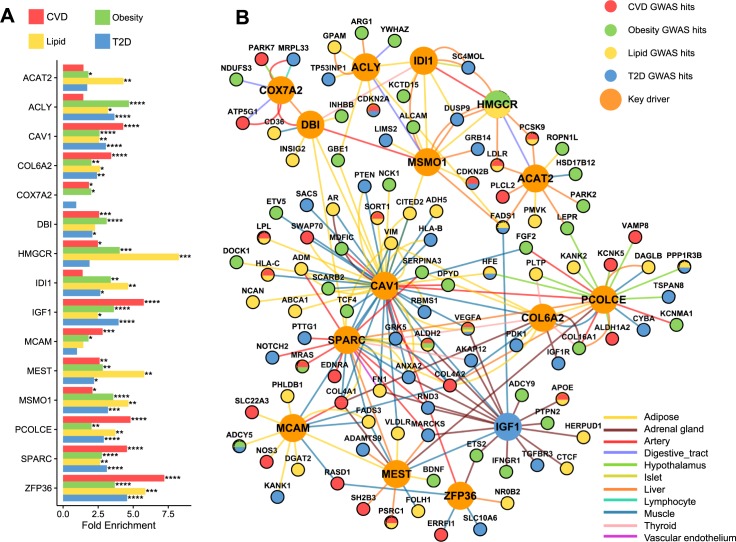
Subnetworks of the top 15 shared KDs orchestrate known genes for CVD, T2D, obesity and lipids. A) Fold enrichment of KD subnetwork genes for known genes related to cardiometabolic traits reported in DisGeNET. *p<0.05, **p<0.01, ***p<0.001, ****p<0.0001. B) Top KD subnetworks with GWAS hits (p < 1e-5 as reported in GWAS Catalog) for cardiometabolic traits. KDs are large nodes. Edge color denotes tissue-origin. Only high-confidence edges (those with weight score in the top 20%) are visualized.

The KDs and subnetworks were identified based on the full spectrum of genetic evidence (from strong to moderate and subtle) from the various GWAS datasets examined in the current study. To assess whether the top KD subnetworks were enriched for previously known disease genes that mostly represent the strong and replicated genes as a means of cross-validation, we manually curated previously reported genes associated with CVD, T2D, and intermediate metabolic traits related to CVD, T2D (glucose, insulin, lipids, obesity) from DisGeNET [32] and the NHGRI GWAS Catalog [6] (**Fig 1C,** genes listed in **S3 Table**). The connection between the top 15 KDs and known genes for CVD, T2D and relevant cardiometabolic traits was confirmed by the significant over-representation of the known disease genes in KD subnetworks, with fold enrichment as large as 8, confirming the strong biological importance of these KDs (**Fig 4A**). Further, the top 15 KDs showed direct connections to 28 GWAS hits reaching genome-wide significance (p < 5e-8) for CVD and 16 for T2D, which account for 35% (fold = 3.35, p = 7.18e-10) and 22% (fold = 2.16, p = 8.08e-4) of all reported significant GWAS signals for CVD and T2D in GWAS catalog, respectively. Two of the 15 top KDs, namely *HMGCR* and *IGF1*, were previously identified as signals of genome-wide significance for obesity, lipids and T2D, all risk factors of CVD. Additionally, network visualization revealed tissue-specific KDs and interactions of CVD and T2D genes in many disease-relevant tissues including adipose, adrenal gland, artery, blood, digestive tract (small intestine, colon), hypothalamus, islet, liver, lymphocyte, skeletal muscle, thyroid, and vascular endothelium (**Fig 4B**). *PCOLCE* represents an intriguing hypothalamus KD that interacts with important energy homeostasis genes like leptin receptor *LEPR*, suggesting a role of neurohormonal control in CVD and T2D pathogenesis. In contrast, *CAV1* appeared to interact extensively with other KDs in peripheral tissues, especially in the adipose tissue.

### Experimental validation of *CAV1* subnetworks using an *in vitro* adipocyte model and *in vivo* knockout mouse model

**Fig 5:**
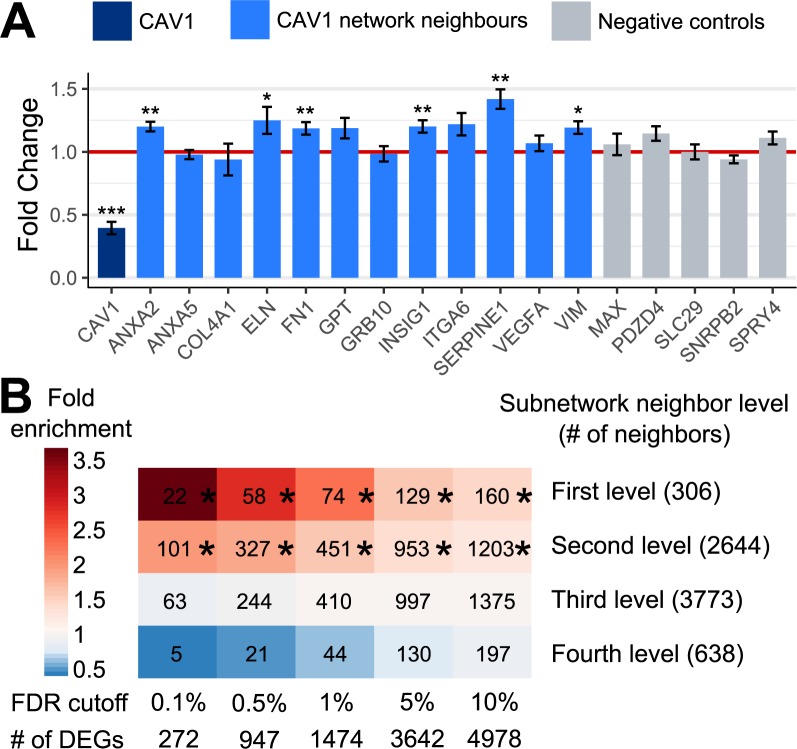
Validation of *CAV1* subnetwork using *in vitro* siRNA knockdown (A) and *in vivo* knockout mouse model (B). A) Fold change of expression level for *CAV1* subnetwork and negative control genes 2 days after *Cav1* knockdown using two siRNAs separately. Twelve *CAV1* neighbors were randomly selected from the first and second level neighboring genes of *CAV1* in adipose network. Five negative controls were randomly selected from the genes not connected to *CAV1* or its first level networks in adipose network. Statistical significance of genes was determined by linear model, adjusting for batch effect and siRNA differences. N = 6/siRNA group, mean ± SEM, *p < 0.05, **p < 0.01, ***p<0.001. B) Overlap of *CAV1* neighboring genes in the adipose tissue subnetwork at various distance levels with the differentially expressed genes in the gonadal adipose tissue in *Cav1* knockout mice (N = 3/group). Overlap p-value is determined by Fisher’s exact test. *Overlap p < 0.05 after Bonferroni correction.

*CAV1* is a robust KD for CVD- and T2D-associated modules across multiple tissues, with the adipose tissue subnetwork of *CAV1* containing the largest number of neighboring genes (**Fig 4B**). In addition, adipose tissue is the only tissue where *CAV1* is a KD in both the Bayesian networks and GIANT networks. These lines of evidence implicate the potential importance of *CAV1* adipose subnetwork in the shared pathogenesis for both diseases. Indeed, *Cav1-/-* mice have been shown to alter the lipid profile, susceptibility to atherosclerosis, and insulin resistance [33, 34]. To assess whether perturbation of this potential KD induces changes in the subnetwork genes as predicted by our network modeling, we performed validation by conducting siRNA-mediated knock down of *Cav1* in differentiating mouse 3T3-L1 adipocytes and by evaluating the whole transcriptome alteration in mouse gonadal adipose tissue between wild type and *Cav1*^*-/-*^ mice [33] (**Fig 1C**; details in **Methods**). Of the 12 adipose network neighbors of *Cav1* that were tested *in vitro*, 6 exhibited significant changes in expression level on day 2 after ~60% *Cav1* knockdown using two siRNAs against *Cav1*. In contrast, none of the 5 negative controls, which were randomly selected among adipocyte genes that are not connected to *Cav1* or its first level neighbors in the adipose network, were affected after *Cav1* perturbation (**Fig 5A**). *Cav1* knockdown also led to decreased expression of *Pparg*, a major adipocyte differentiation regulator (**S6 Fig**), supporting a role of *Cav1* in adipocyte differentiation as previously observed [35].

In 3-month-old *Cav1*^*-/-*^ mice which showed perturbed lipid and insulin sensitivity profiles, we observed 1,474 differentially expressed genes (DEGs) at FDR<1%. We found that the first and second level neighbors of *CAV1* in our predicted subnetwork showed significant enrichment for DEGs in adipose tissue induced by *Cav1* knockout, with the degree of fold enrichment increasing as the statistical cutoff used to define DEGs became more stringent (**Fig 5B**; subnetwork view with DEGs in **S7 Fig**). On the contrary, the third and fourth level neighbors of *CAV1* in our predicted subnetwork did not exhibit such enrichment of DEGs (**Fig 5B**). These experimental findings support that *CAV1* is a key regulator of the subnetwork and the network structure predicted by our network modeling is reliable, although it is difficult to discern whether the network changes are related to alterations in adipocyte differentiation status. We also observed strong enrichment for the focal adhesion pathway in both the predicted *Cav1* adipose subnetwork (p = 9.6e-14 by Fisher’s exact test, fold enrichment = 6.0) and the differential adipose genes in *Cav1*-/- mice (p = 6.9e-9, fold enrichment = 3.5).

### Shared KDs are associated with CVD and T2D traits in experimental mouse models

**Fig 6:**
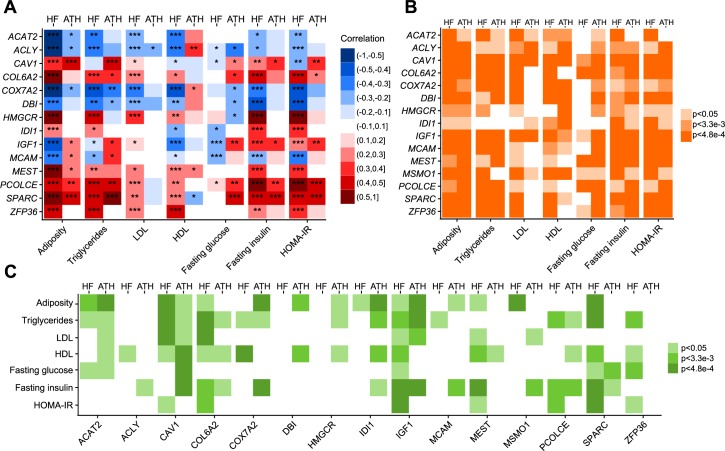
Associations of KDs and subnetworks with cardiometabolic traits in mice. (A) Association between KD expression and cardiometabolic traits in adipose tissue from HF-HMDP (HF) and aorta tissue from atherogenic-HMDP (ATH) as determined by Pearson correlation. *p< 0.05; **p< 0.05 after Bonferroni correction for the KD number; ***p< 0.05 after Bonferroni correction for the number of KDs and traits. (B) Transcriptomic-wide association of KD subnetworks and cardiometabolic traits in adipose tissue from HF-HMDP, and aorta tissue from atherogenic-HMDP, as evaluated by MSEA. (C) Genome-wide genetic association of KD subnetworks and cardiometabolic traits based on adipose eQTL mapping in HF-HMDP, and aorta eQTL mapping in Atherogenic-HMDP, as determined by MSEA. p<0.05, p<3.3–3, and p<4.8e-4 correspond to uncorrected and Bonferroni-corrected p-values (correcting for the number of KDs or for the number of KD and trait combinations).

We further assessed the transcriptomic profiling in adipose (relevant to T2D and CVD) and aorta tissue (main site of CVD) in relation to 7 cardiometabolic phenotypes including adiposity, lipid levels (triglyceride, LDL, HDL), fasting glucose, fasting insulin and HOMA-IR, across >100 mouse strains in two HMDP panels [21–23]. HMDP is a systems genetics resource that comprises more than 100 commercially available mouse strains differing in genetic composition, and has emerged as a power tool to study complex human diseases [22, 36]. The biological relevance of HMDP to human pathophysiology has been reproducibly demonstrated [37–39]. Moreover, HMDP data was completely independent of the human-focused genetic datasets and the network datasets used in our primary integrative analysis (**Fig 1C**). Here we selected two specific HMDP panels, high-fat (HF) and atherogenic (ATH), in which mice were either fed with a high-fat high-sucrose diet or underwent transgenic expression of human APOE-Leiden and CETP gene as a pro-atherogenic background, respectively. These two panels were chosen for their representativeness of human T2D (the HF panel) and CVD (the ATH panel) pathology. First, we investigated the correlation between the expression of 14 top KDs (no probe for KD *MSMO1* in HMDP) and cardiometabolic traits in the adipose and aorta tissues assessed in HMDP. All 14 KDs displayed significant trait association in HMDP, with the association for 11 KDs replicated in both the HF and ATH HMDP panels (**Fig 6A**). Next, we retrieved the adipose and aorta gene-trait correlation statistics for the top KD subnetwork genes, and used MSEA to test whether genes in the KD subnetworks displayed an overall overrepresentation of strong trait association in HMDP. Again, the 14 KD subnetworks showed significant trait association after Bonferroni correction (**Fig 6B**). These findings support that the close involvement of the KDs in cardiometabolic trait perturbation we predicted based on human datasets can be cross-validated in mouse models.

### Causal implication of the shared KD subnetworks in experimental mouse models

*Cav1* knockout in mice led to dysreuglation of the predicted subnetwork (**Fig 5B**) and significant alterations in cardiometabolic phenotypes [33, 34], supporting the causal role of *CAV1* in both CVD and T2D. To further investigate the potential causal role of the top KDs and their subnetworks in CVD and T2D, we conducted integrative analysis of the KD subnetworks to assess their disease association using GWAS results for the 7 cardiometabolic traits from HMDP and tissue-specific cis-eQTLs (**Fig 1C**). By mapping GWAS signals to genes using adipose or aorta eQTLs and testing for enrichment of genetic association with cardiometabolic traits within the KD subnetwork genes using MSEA, we found consistent and significant association between cardiometabolic traits and the subnetworks of KDs *ACAT2*, *CAV1*, *COL6A2*, *IGF1*, *PCOLCE*, and *SPARC* across adipose and aorta (**Fig 6C**). These results informed by mouse GWAS support a potential causal role of these top KDs in perturbing gene networks in multiple tissues to trigger CVD and T2D.

## Discussion

CVD and T2D are highly correlated complex diseases and share many common risk factors. Multiple genetic variants may individually exert subtle to strong effects on disease pathogenesis, and in aggregate perturb diverse pathogenic pathways [8, 9, 13, 19, 20, 40]. In this systems-level, data-driven analysis of GWAS from several large and high-quality cohorts of diverse ethnicities, integrated with functional data (from ENCODE, eQTLs, tissue-specific co-expression and regulatory networks constructed from human and mouse experiments), we identified both known and novel pathways and gene subnetworks that were genetically linked to both CVD and T2D across cohorts and ethnicities. Further, KDs in tissue-specific subnetworks appear to regulate many known disease genes for increased risk of CVD and T2D. Lastly, we experimentally validated the network topology using *in vitro* adipocyte and data from *in vivo* gene knockout models, and confirmed the role of the top KDs and subnetworks in both CVD and T2D traits in independent sets of mouse studies.

The data-driven nature of the current study offers several strengths. First, we incorporated the full-scale of genetic variant-disease association from multiple cohorts, ethnicities and disease endpoints, allowing for the detection of subtle to moderate signals, as well as comparison and replication of results across diseases and populations. More importantly, by focusing on results that demonstrate consistent significance at pathway and network level, we overcome the difficulties in harmonizing independent datasets that are complicated by substantial heterogeneity due to platform differences and population substructure. This is because disease signals across populations are more conserved at pathway level than at individual variant and gene levels [12, 14, 16]. Second, the comprehensive incorporation of tissue-specific eQTLs, coupled with the use of tissue-specific networks, enhances our ability to achieve better functional mapping between genetic variants and genes, and uncover systems-level regulatory circuits for CVD and T2D in a tissue-specific fashion. Third, data-driven modules and networks used in this study increase the potential for novel discovery as gene-gene interactions are defined by data rather than prior knowledge. As the network models were from many independent studies reflecting diverse physiological conditions, leveraging these datasets and network models offers more comprehensive coverage of biological interactions than any given dataset can provide and has proven a valuable approach to unveil novel biological insights [9, 13, 41]. While some of our findings confirmed those from previous canonical pathway-based analysis on disease processes including ECM-receptor interaction and cell-adhesion, and KDs such as *SPARC* [8], our data-driven approach in the current study uncovered numerous novel genes, pathways, and gene subnetworks. A likely reason for the enhanced discovery potential of co-expression modules is that several interacting pathways could be co-regulated in a single module, or a pathway could interact with other poorly annotated processes in a module to together confer disease risk. The use of modules capturing such interactions improves the statistical power, in contrast to testing the pathways individually. Lastly, we conducted cross-validation studies in support of the functional roles of specific KDs and subnetworks in CVD and T2D using independent experimental models.

We acknowledge the following limitations in our study. First, our analyses were constrained by the coverage of functional datasets that are currently available, which causes uneven tissue coverage between data types and statistical bias towards more commonly profiled tissues such as adipose and liver, making it difficult to achieve precise inference for all relevant tissues. Although we believe this does not necessarily undermine the validity of the main findings from our study, we acknowledge that we likely have missed relevant biology from tissues with fewer studies and smaller sample sizes. Further investigation is needed when additional relevant datasets become available. Secondly, our FDR estimates in MSEA do not take into consideration the gene overlap structure among co-expression modules, due to the challenge in accurately adjusting for the various degrees of overlaps between module pairs. To alleviate this limitation, we focus on modules and pathways demonstrating consistency across datasets and merge overlapping modules subsequently. Thirdly, although we conducted validation experiments on the *CAV1* subnetwork in both *in vitro* and *in vivo* models and cross-validated the importance of the predicted top key drivers and subnetworks in two independent large-scale mouse population studies, further experiments are warranted to thoroughly test the causality of the predicted KDs and elucidate the detailed tissue-specific mechanisms of the KDs on CVD and T2D. This is particularly important considering the limited overlaps in the modules and KDs identified from our study and the ones identified in two recent multi-tissue network analysis of cardiometabolic diseases [10, 11]. Only 7 KDs overlapped including *APOA1*, *CD2*, *CEBPD*, *CENPF*, *CSF1R*, *CTSS*, *UBE2S*. Methodological differences in network inference and key driver analysis and differences in the pathophysiological conditions of the study populations could explain the discrepancies. Lastly, ethnic-specific and sex-specific mechanisms await future exploration.

There are several direct implications that can be drawn from the results of our integrative analyses of both observational and experimental data. First, it appears that pathogenic pathways for CVD and T2D are indeed common in ethnically diverse populations. These shared pathways capture most of the critical processes that have been previously implicated in the development of either T2D or CVD, including metabolism of lipids and lipoproteins, glucose, fatty acids, bile acids metabolism, biological oxidation, coagulation, immune response, cytokine signaling, and PDGF signaling. Second, BCAA metabolism and ECM are among the top and common pathways identified. Our finding on BCAA is consistent with recent work relating serum levels of BCAA to risk of CVD and T2D in large prospective cohorts [42, 43], although whether BCAA is a “pathophenotype” or strong pathogenic factor has been debated [28, 44]. Our findings support a causal role of BCAA because 1) both CVD and T2D risk variants were enriched in the co-expression modules related to BCAA degradation, and 2) 15 genes in the BCAA pathway were part of the top KD subnetworks, representing a significant enrichment of BCAA genes (fold enrichment = 3.02, Fisher’s exact test p = 1.4e-5). Of note, BCAA genes themselves carry few genetic risk variants for CVD and T2D, albeit their network neighboring genes are highly enriched for disease variants, which may result from negative evolutionary pressure due to the critical role of BCAA. More recently, Jang and colleagues have shown BCAA catabolism can cause insulin resistance, providing further support for the causal role of BCAA for both CVD and T2D [45]. Our finding on the role of ECM in both CVD and T2D is also in line with recent reports [8, 13, 29, 30, 46]. In the top enriched subnetworks, ECM genes appear to exert strong effect (**Fig 4B**) coordinating other processes such as cholesterol metabolism, energy homeostasis, and immune response across a wide range of peripheral tissues and endocrine axis. This substantiates the importance of ECM modeling as a mechanistic driver for CVD and T2D.

Secondly, our comprehensive network modeling identified critical disease modulators and key targets whose functional roles were subsequently supported by multiple cross-validation efforts. This supports the use of network modeling to unravel and prioritize promising top targets that may have high pathogenic potential for both CVD and T2D. The KDs we identified can be considered as “highly confident” for the following reasons: 1) they are KDs for both CVD and T2D associated modules, 2) the tissue-specific subnetworks of these KDs show significant and replicable association with both diseases, 3) their subnetworks are highly enriched with known CVD and T2D genes, 4) *in vitro* siRNA knockdown and *in vivo* knockout mouse experiments confirm the role of a central KD *CAV1* in regulating the downstream genes as predicted in our network model, and 5) both the expression levels of KDs and the genetic variants mapped to the KD subnetworks are significantly associated with CVD and T2D relevant traits in independent mouse populations with naturally occurring genetic variations.

Thirdly, most KDs are not GWAS signals reaching genome-wide significance, nor are they rare-variant carrying genes, indicating that standard genetic studies miss important genes that orchestrate known CVD and T2D genes. The phenomenon may reflect a negative evolutionary pressure experienced by the KDs due to their crucial functions. In support of this hypothesis, we found a significant enrichment of human essential genes lacking functional variations among the 162 KDs identified in our study [47] (Fold = 1.41, p = 9.02e-3). This is consistent with previous findings [8, 9, 13] reaffirming the power and reliability of our approach in uncovering hidden biological insights particularly in a systematic integrative manner.

The connections between KDs and other disease genes revealed by our study warrant future investigation into the potential gene-gene interactions. Indeed, a closer examination of the biological functions from the top shared KDs further corroborates their disease relevance. For instance, our network modeling identified *HMGCR* as a top KD, consistent with its primary role as the target for cholesterol-lowering HMG-CoA inhibitors, namely statins. Our directionality inference analysis indicates that *HMGCR* is associated with CVD and T2D in opposite directions. This is consistent with the recent findings that genetic variations in *HMGCR* that decrease CVD risk cause slightly increased T2D risk, and statin drugs targeting *HMGCR* reduces CVD risk but increases T2D risk [48–50]. *CAV1* and *IGF1* represent two tightly connected multi-functional KDs. *CAV1* null mice were found to have abnormal lipid levels, hyperglycemia, insulin resistance and atherosclerosis [33, 34]. Consistent with these observations, we found strong association of *CAV1* expression levels as well as *CAV1* network with diverse cardiometabolic traits in both human studies and mouse HMDP panels. Our data-driven approach also revealed the central role of *CAV1* in adipose tissue by elucidating its connection to a large number of CVD and T2D GWAS genes and to genes involved in focal adhesion and inflammation (**Fig 4**), which could drive adipocyte insulin resistance [51, 52]. The regulatory effect of *CAV1* on neighboring genes was subsequently validated using *in vitro* adipocyte and *in vivo* mouse models. Moreover, our network modeling also captured the central role of *CAV1* in muscle and artery tissues, suggesting multi-tissue functions of *CAV1* in the pathogenic crossroads for CVD and T2D. The other multi-functional KD, *IGF1*, is itself a GWAS hit for fasting insulin and HOMA-IR. Despite being primarily secreted in liver, in our study *IGF1* demonstrated an adrenal gland and muscle specific regulatory circuit with CVD and T2D genes, suggesting that it may confer risk to these diseases through the adrenal endocrine function and muscle insulin sensitivity. The three ECM KDs we identified, *SPARC*, *PCOLCE* and *COL6A2*, were especially interesting due to their consistent and strong impact on diverse cardiometabolic traits shown in our cross-validation analyses in HMDP (**Fig 4, Fig 6**). *SPARC* encodes osteonectin, which is primarily circulated by adipocytes. It inhibits adipogenesis and promotes adipose tissue fibrosis ^50^. *SPARC* is also associated with insulin resistance and coronary artery lesions ^51, 52^. *PCOLCE* (procollagen C-endopeptidase enhancer) represents a novel regulator for hypothalamus ECM that could potentially disrupt the neuroendocrine system. *COL6A2*, on the other hand, provides new insights into how collagen may affect cardiometabolic disorders: in adrenal tissue *COL6A2* is connected to *IGF1R*, the direct downstream effector for KD *IGF1*. Importantly, our directionality analysis suggests that while some KDs such as *CAV1* may have similar directional effects on CVD and T2D, cases like *HMGCR* that show opposite effects on these diseases are also present. Therefore, it is important to test the directional predictions to prioritize targets that have the potential to ameliorate both diseases and deprioritize targets with opposite effects on the two diseases.

In summary, through integration and modeling of a multitude of genetics and genomics datasets, we identified key molecular drivers, pathways, and gene subnetworks that are shared in the pathogenesis of CVD and T2D. Our findings offer a systems-level understanding of these highly clustered diseases and provide guidance on further basic mechanistic work and intervention studies. The shared key drivers and networks identified may serve as more effective therapeutic targets to help achieve systems-wide alleviation of pathogenic stress for cardiometabolic diseases, due to their central and systemic role in regulating scores of disease genes. Such network-based approach represents a new avenue for therapeutic intervention targeting common complex diseases.

## Methods

### Identification of qualified SNPs from GWAS of CVD and T2D

Detailed GWAS information including sample size, ethnicity and genotyping platform was described in **S4 Table** and **S1 Text**. Briefly, p-values of qualified single nucleotide polymorphisms (SNPs) at minor allele frequency > 0.05 and imputation quality > 0.3 for CVD and T2D were collected for all available GWAS datasets (WHI-SHARe, WHI-GARNET, JHS, FHS, CARDIoGRAMplusC4D [19], and DIAGRAM [20]). SNPs meeting the following criteria were further filtered out: 1) ranked in the bottom 50% (weaker association) based on disease association p-values and 2) in strong linkage disequilibrium (LD) (r2 > 0.5) according to ethnicity-specific LD data from Hapmap V3 [53] and 1000 Genomes[54]. For each GWAS dataset, LD filtering was conducted by first ranking SNPs based on the association p values and then checking if the next highest ranked SNP was in LD with the top SNP. If the r^2^ was above 0.5, the SNP with lower rank was removed. The step was repeated by always checking if the next SNP was in LD with any of the already accepted ones.

### Curation of data-driven gene co-expression network modules

Using the Weighted Gene Co-expression Network Analysis (WGCNA)[55], we constructed gene co-expression modules capturing significant co-regulation patterns and functional relatedness among groups of genes in multiple CVD- or T2D-related tissues (including aortic endothelial cells, adipose, blood, liver, heart, islet, kidney, muscle and brain) obtained from nine human and mouse studies (**S5 Table**). Modules with size smaller than 10 genes were excluded to avoid statistical artifacts, yielding 2,672 co-expression modules. These coexpression modules were used as a collection of data-driven sets of functionally connected genes for downstream analysis. The potential biological functions of each module were annotated using pathway databases Reactome and KEGG, and statistical significance was determined by Fisher’s exact test with Bonferroni-corrected p< 0.05.

### Curation of functional genomics from eQTLs and ENCODE

eQTLs establish biologically meaningful connections between genetic variants and gene expression, and could serve as functional evidence in support of the potential causal role of candidate genes in pathogenic processes[56, 57]. We therefore conducted comprehensive curation for significant eQTLs in a total of 19 tissues that have been identified by various consortia (including the Genotype-Tissue Expression (GTEx) [58], Muther [59] and Cardiogenics [60], and additional independent studies; **S6 Table)**. Additional functional genomics resources from ENCODE were also curated to complement the eQTLs for SNP-gene mapping (**S1 Text**).

### Identification of genetically-driven CVD and T2D modules using Marker Set Enrichment Analysis (MSEA)

MSEA was used to identify co-expression modules with over-representation of CVD- or T2D-associated genetic signals for each disease GWAS in each cohort/ethnicity in a study specific manner. MSEA takes into three input: 1) Summary-level results of individual GWAS (WHI, FHS, JHS, CARDIoGRAM+C4D, DIAGRAM) for the LD-filtered SNPs; 2) SNP-gene mapping information, which could be determined by tissue-specific cis-eQTLs, ENCODE based functional annotation and chromosome distance based annotation. Cis-eQTLs is defined as eQTLs within 1MB of the transcription starting sites of genes. For ENCODE, we accessed the Regulome database and used the reported functional interactions to map SNPs to genes by chromosomal distance. Only SNPs within 50kb of the gene region and have functional evidence in Regulome database were kept; 3) Data-driven co-expression modules from multiple human and mouse studies as described above. Tissue-specificity was determined by the tissue origins of eQTLs and ethnic specificity was determined by the ethnicity of each GWAS cohort, since the human disease genetic signals and human eQTL mapping were the main driving factors to determine the significance of the modules. MSEA employs a chi-square like statistic with multiple quantile thresholds to assess whether a co-expression module shows enrichment of functional disease SNPs compared to random chance [14]. The varying quantile thresholds allows the statistic to be adoptable to studies of varying sample size and statistical power. For the list of SNPs mapped to each gene-set, MSEA tested whether the SNP list exhibited significant enrichment of SNPs with stronger association with disease using a chi-square like statistic: $\chi =\sum_{i=1}^{n}\frac{O_{i}-E_{i}}{\sqrt{E_{i}}+\kappa }$, where n denotes the number of quantile points (we used ten quantile points ranging from the top 50% to the top 99.9% based on the rank of GWAS p values), O and E denote the observed and expected counts of positive findings (i.e. signals above the quantile point), and κ = 1 is a stability parameter to reduce artefacts from low expected counts for small SNP sets. The null background was estimated by permuting gene labels to generate random gene sets matching the gene number of each co-expression module, while preserving the assignment of SNPs to genes, accounting for confounding factors such as gene size, LD block size and SNPs per loci. For each co-expression module, 10000 permuted gene sets were generated and enrichment P-values were determined from a Gaussian distribution approximated using the enrichment statistics from the 10000 permutations and the statistics of the co-expression module. Finally, Benjami-Hochberg FDR was estimated across all modules tested for each GWAS.

To evaluate a module across multiple GWAS studies, we employed the Meta-MSEA analysis in Mergeomics, which conducts module-level meta-analysis to retrieve robust signals across studies. Meta-MSEA takes advantage of the parametric estimation of p-values in MSEA by applying Stouffer’s Z score method to determine the meta-Z score, then converts it back to a meta P-value. The meta-FDR was calculated using Benjamini-Hochberg method. Co-expression modules with meta-FDR < 5% were considered significant and included in subsequent analyses.

### Identification of key drivers and disease subnetworks

We used graphical gene-gene interaction networks including the GIANT networks [31] and Bayesian networks (BN) from 25 CVD and T2D relevant tissue and cell types **(S7 Table**, **S1 Text**) to identify KDs. If more than one dataset was available for a given tissue, a network was constructed for each dataset and all networks for the same tissue were combined as a union to represent the network of that tissue, with the consistency of each network edge across datasets coded as edge weight. The co-expression modules genetically associated with CVD or T2D identified by Meta-MSEA were mapped onto these graphical networks to identify KDs using the weighted key driver analysis (wKDA) implemented in Mergeomics [14]. wKDA uniquely consider the edge weight information, either in the form of edge consistency score in the case of BNs or edge confidence score in the case of GIANT networks. Specifically, a network was first screened for suitable hub genes whose degree (number of genes connected to the hub) is in the top 25% of all network nodes. Once the hubs have been defined, their local one-edge neighborhoods, or “subnetworks” were extracted. All genes in each of the CVD and T2D-associated gene sets that were discovered by meta-MSEA were overlaid onto the hub subnetworks to see if a particular subnetwork was enriched for the genes in CVD/T2D associated gene sets. The edges that connect a hub to its neighbors are simplified into node strengths (strength = sum of adjacent edge weights) within the neighborhood, except for the hub itself. The test statistic for the wKDA is analogous to the one used for MSEA: $\chi =\frac{O-E}{\sqrt{E}-\kappa }$, except that the values O and E represent the observed and expected ratios of genes from CVD/T2D gene sets in a hub subnetwork. In particular, $E=\frac{N_{k}N_{p}}{N}$ is estimated based on the hub degree *N*_*k*_, disease gene set size *N*_*p*_ and the order of the full network *N*, with the implicit assumption that the weight distribution is isotropic across the network. Statistical significance of the disease-enriched hubs, henceforth KDs, is estimated by permuting the gene labels in the network for 10000 times and estimating the P-value based on the null distribution. To control for multiple testing, stringent Bonferroni adjustment was used to focus on the top robust KDs. KDs shared by CVD and T2D modules are prioritized based on the following criteria: i) Bonferroni-corrected p< 0.05 in wKDA, ii) replicated by both GIANT networks and Bayesian networks, and iii) the genetic association strength between the KD subnetworks (immediate network neighbors of the KDs) and CVD/T2D in Meta-MSEA. Finally, Cytoscape 3.3.0 [61] was used for disease subnetwork visualization.

### Inference of the direction of genetic effects of KD subnetworks

We used the genetic effect direction of KDs as a proxy for probable effect direction of the KD subnetworks. For each KD, we retrieved their tissue-specific eQTLs as well as variants within 50kb of the gene region, whose genetic association information was available in both CARDIoGRAMplusC4D and DIAGRAM, the two large meta-consortia of GWAS for CVD and T2D. CVD/T2D association beta-values of mapped variants of KDs were then extracted, and the signs of beta-values were examined to ensure they were based on the same reference alleles between GWAS. Lastly, for all mapped variants on each KD, the signs of the beta-value for CVD and T2D were compared and statistical significance of the proportion of variants with similar or opposite effect direction between diseases was determined by z-test.

### Validation of KD subnetwork topology using siRNA knockdown in adipocytes

We chose to validate the predicted adipose subnetwork of a top ranked KD of both CVD and T2D, *Cav1*, in 3T3-L1 adipocytes. Cells were cultured to confluence and adipocyte differentiation was induced using MDI differentiation medium (**S1 Text**). Two days after the initiation of differentiation, cells were transfected with 50 nM *Cav1* siRNAs (3 distinct siRNAs were tested and two of the strongest ones were chosen) or a scrambled control siRNA. For each siRNA, two separate sets of transfection experiments were conducted, with three biological replicates in each experiment. Two days after transfection, cells were collected for total RNA extraction, reverse transcription and quantitative PCR measurement of 12 predicted *Cav1* subnetwork genes and 5 random genes not within the subnetwork as negative controls (**S1 Text**). β-actin was used to normalize the expression level of target genes.

### Validation of KD subnetwork topology using Cav1 null mice

We accessed the gonadal white tissue gene expression data of 3-month-old wild type and *Cav1*^*-/-*^ male mice (N = 3/group) from Gene Expression Omnibus (GEO accession: GSE35431). Detailed description of the data collection procedures has been described previously [33]. Gene expression was profiled using Illumina MouseWG-6 v2.0 expression beadchip and normalized using robust spline. Differentially expressed genes (DEGs) between genotype groups were identified using linear model implemented in the R package Limma and false discovery rate was estimated using the Benjamini-Hochberg procedure [62]. DEGs at different statistical cutoffs were compared to *CAV1* subnetwork genes at different levels (i.e., 1, 2, 3, or 4 edges away from *CAV1*) to assess overlap and significance of overlap was evaluated using Fisher’s exact test.

### Validation of KD subnetworks using mouse HMDP studies

To further validate the role of KD subnetworks in CVD and T2D, we incorporated genetic, genomic and transcriptomic data from HMDP (comprised of >100 mouse strains differing by genetic composition) [21–23]. HMDP data was from two panels, one with mice fed with a high-fat diet (HF-HMDP)[22], and the other with hyperlipidemic mice made by transgenic expression of human APOE-Leiden and CETP gene (ATH-HMDP)[23]. For HF-HMDP, we retrieved gene-trait correlation data for adipose tissue (due to its relevance to both CVD and T2D) and 7 core cardiometabolic traits including adiposity, fasting glucose level, fasting insulin level, LDL, HDL, triglycerides and homeostatic model assessment-insulin resistance (HOMA-IR). For ATH-HMDP, we retrieved aorta gene-trait correlation (aorta tissue is the main site for CVD in mice) for all 7 traits. In addition to assessing the trait association strengths of individual KDs, we also used MSEA to evaluate the aggregate association strength of the top CVD/T2D KD subnetworks with the traits at both transcription and genetic levels through transcriptome-wide association (TWAS) and GWAS in HF-HMDP and ATH-HMDP (**S1 Text**).

## Supporting information

S1 Text Supplemental methods and references. (DOCX)

S1 Fig Number of significant co-expression modules found by different gene-SNP mapping types. (EPS)

S2 Fig Heatmap of pair-wise overlapping ratio (Jaccard index) between the 79 co-expression modules associated with CVD (y-axis) and 54 modules (x-axis) associated with T2D. (EPS)

S3 Fig Overlap ratio plots between co-expression modules and the annotated functional terms. All the annotated pathways reach >5% overlap ratio either on the pathway side or on the module side. Specifically, a majority (251 out of 278, 90.3%) of the annotated pathways had > = 5% of genes overlapping with the modules to which they were assigned. For the 27 annotated pathways where <5% pathway genes were represented, these were the cases where the co-expression modules were small and the pathways were large, but all of them showed overlap of >5% module genes. The minimum, maximum, mean and median numbers of the overlapping genes for the annotations are 5, 170, 19 and 13, respectively.
(EPS)

S4 Fig Concept of key driver analysis (KDA). KDA requires gene regulatory networks capturing gene-gene interactions. Hub genes that show high degrees of connections to other networks genes are first identified, and their adjacent network neighbors (subnetworks) were extracted. All genes in each CVD/T2D associated module are used as input and mapped onto each hub subnetwork to assess whether a hub subnetwork was enriched for the genes in the input modules. The hubs whose subnetworks show significant enrichment of CVD/T2D module genes are defined as potential key drivers.
(EPS)

S5 Fig Scatter plots of the GWAS beta-values of variants mapped to the top 15 KDs. (A) Gene-variant mapping based on eQTLs only; (B) Gene-variant mapping based on eQTLs and chromosomal distance. Percentage indicates the proportion of mapped variants with the same effect direction between CVD and T2D. Statistical significance of the difference of the proportion from random expectation is determined by z-test.
(EPS)

S6 Fig Expression changes in adipocyte differentiation state markers 3 days after the in vitro siRNA knockdown of Cav1. Statistical significance of genes was determined by Student’s t-test. N = 3/group, mean ± SEM, **p < 0.01.
(EPS)

S7 Fig Visualization of CAV1 adipose subnetwork. Red color indicates significantly up-regulated genes (FDR < 1%) in *Cav1*^-/-^ mice, and blue color indicates significantly down-regulated genes (FDR < 1%) in *Cav1*^-/-^ mice.
(EPS)

S1 Table Summary of significant co-expression modules (FDR < 5%) associated with CVD or T2D. (DOCX)

S2 Table Functional annotation terms of the significant co-expression modules. (DOCX)

S3 Table List of previously reported genes associated with CVD, T2D, and intermediate metabolic traits related to CVD, T2D from DisGeNET and GWAS Catalog. (DOCX)

S4 Table Summary information of genome-wide association studies. (DOCX)

S5 Table Data resources and references for co-expression networks. (DOCX)

S6 Table Data resources and references for expression QTLs. (DOCX)

S7 Table Data resources and references for gene-gene regulatory networks. (DOCX)
