# Supplementary material for: Shared genetic regulatory networks for cardiovascular disease and type 2 diabetes in multiple populations of diverse ethnicities in the United States
Source: PLoS Genet. 2017 Sep 28;13(9):e1007040. doi: 10.1371/journal.pgen.1007040 (PMC5634657; doi:10.1371/journal.pgen.1007040)
Supplement: S1 Text — (DOCX) [file pgen.1007040.s001.docx]

# S1 Text. Supplemental Methods and References

## Supplemental Methods

## Genome-wide Association Studies of CVD and T2D

A total of five GWAS were incorporated in the study, including WHI, JHS, FHS, CARDIoGRAMplusC4D and DIAGRAM. All studies were approved by institutional review committees and acquired informed consent from participants. WHI, JHS, and FHS are independent studies. Although FHS was included as a part of CARDIoGRAMplusC4D and DIAGRAM, the sample size of FHS (n=4461 for CVD, n=8338 for T2D) was only 2-7% of CARDIoGRAMplusC4D (n=184305) and DIAGRAM (n=110452). Thus, we consider all five GWAS as independent with each other. Individual summary level GWAS data used in the study was first pruned to only keep the SNPs in the top 50% range ranked by p-values to reduce noise from weak signals[[1](#_ENREF_1)]. Remaining SNPs were further filtered for linkage disequilibrium using r^2^ > 0.5 as the cutoff. Ethnicity-specific pairwise r^2^ values of SNPs were retrieved from Hapmap v3 [[2](#_ENREF_2)] and 1000 Genomes data [[3](#_ENREF_3)].

## Gene-SNP Mapping Resources

In additional to eQTL data, we included five additional gene-SNP mapping resources, 1) combining all tissue-specific eSNPs into a single multi-tissue eSNP set, 2) chromosomal distance mapping based on a 50kb window, 3) ENCODE project based Regulome SNPs[[4](#_ENREF_4)], where only SNPs supported by at least two different kinds of RegulomeDB functional evidences are mapped to genes based on a 50kb window, 4) merging eSNP sets with Regulome data, 5) combining all available mapping resources. As a result, a total of 21 sets of SNP-gene mapping were used for each co-expression module.

**Mergeomics Package**

“Mergeomics” is an open-source bioconductor package (<https://bioconductor.org/packages/devel/bioc/html/Mergeomics.html>) used to capture systems-level pathological perturbation from multiple layers of biological data such as genetics, genomics, transcriptomics, epigenomics, canonical pathways and gene networks.

**Functional Annotation of Canonical Pathways**

We collected 186 KEGG pathways and 674 Reactome pathways from MsigDB [[5](#_ENREF_5)]. Functional categories were assigned to modules if Bonferroni-corrected enrichment reached P < 0.05 and there are more than five overlapping genes. For the over-represented functional categories, we considered those that had > 10% overlap ratio as redundant and only kept the categories with the highest number of associated disease modules. This was achieved by first ranking the annotations according to the number of linked modules, and then recursively removing the terms that have ≥ 10% overlapping ratio with the higher ranked annotation terms. The designation of trait, study and ethnicity to each of the functional categories was determined by the assigned modules.

**Bayesian Networks of Gene-Gene Interactions**

We utilized Bayesian network models of gene-gene interactions that take into account both the genotypes that affect gene expression (causal direction known), and the statistical relationships between gene expression levels (causal direction uncertain), using the established method by Zhu et al. [[6](#_ENREF_6), [7](#_ENREF_7)]. Bayesian network models from human and mouse studies were constructed based on genetics and gene expression data generated from multiple tissues from previously published studies, each involving hundreds of individuals (details and references in **S7 Table**).

Bayesian networks are directed acyclic graphs in which the edges of the graph are defined by conditional probabilities that characterize the distribution of states of each gene given the state of its parents [[8](#_ENREF_8)]. The network topology defines a partitioned joint probability distribution over all genes in a network. The likelihood of a Bayesian network model given observed genomic data is determined using Bayes formula. For each dataset, 1000 Bayesian networks, each using different random seeds, were reconstructed using Monte Carlo Markov Chain simulation [[9](#_ENREF_9)]. Bayesian Information Criteria was used to determine the model with the best fit for each network. From the resulting set of 1000 networks, edges that appeared in greater than 30% of the networks were used to define a consensus network for a given dataset. To infer causal directions between genes in a network, genetic information was used as priors by allowing genes with *cis*-eSNPs to be parent nodes of genes without *cis*-eSNPs and preventing genes without *cis*-eSNPs to be parents of genes with *ci*s-eSNPs [[10](#_ENREF_10)]. Bayesian network provides a natural framework for integrating diverse data and reconstruct biological causal networks.

**GIANT Networks of Gene-Gene Interactions**

We downloaded the raw networks of relevant tissues from GIANT database [[11](#_ENREF_11)]. The original connectivity of the GIANT networks was too high, which breaks the scale-free assumption of biological network topology and may lead to increased number of spurious results in subsequent key driver analysis. Thus we performed a filtering algorithm that step-wisely removed the networks edges with low confidence score and recursively evaluated the scale-freeness of the network. The removal process terminated as soon as the network reached boundary scale-freeness as defined by the criterion that at least 25% nodes has a degree (number of genes connected to the node] of 1.

**Extraction of Disease/trait Associated Genes from DisGeNET and GWAS Catalog**

Only genes with disease association score > 0.01 were extracted from DisGeNET [[12](#_ENREF_12)] (accounting for ~ top 10% of genes reported in DisGeNET). The reported traits in DisGeNET were categorized into major groups as follows, 1) CVD: "Atherosclerosis", "Cardiovascular Diseases", "Acute myocardial infarction", "Myocardial Infarction", "Myocardial Ischemia", "Brain Ischemia", "Ischemia"; 2) T2D: "Insulin Resistance", "Impaired glucose tolerance", "Diabetes Mellitus, Non-Insulin-Dependent"; 3) Obesity: "Obesity", "Obesity, Abdominal", "Obesity, Visceral", "Obesity, Morbid", "Adult-onset obesity", "Moderate obesity", "Familial obesity", "Hypothalamic obesity", "Overweight and obesity", "Overweight", "Body Weight", "Moderate obesity", "Familial obesity", "Hypothalamic obesity", "Overweight and obesity", "Overweight", "Body Weight"; 4) Lipid: "Dyslipidemias", "Hyperlipidemia", "Hypercholesterolemia", "Hypercholesterolemia, Familial".

The reported traits in GWAS catalog [[13](#_ENREF_13)] were categorized into major groups as follows, 1) CVD: "Coronary heart disease", "Coronary artery disease", "Coronary artery disease or ischemic stroke", "Coronary artery disease or large artery stroke", "Myocardial infarction", "Ischemic stroke", "Large artery stroke", "Stroke (ischemic)"; 2) T2D: Type 2 diabetes; 3) Obesity: "Body mass index", "Obesity", "Obesity (early onset extreme)", "Obesity (extreme)"; 4) Lipid: "HDL cholesterol", "Cholesterol, total", "Triglycerides", "LDL cholesterol". The latest results from CARDIoGRAM were not yet included in GWAS catalog and were manually added into the GWAS gene list for CVD.

The full list of CVD/T2D associated genes are shown in the **S3 Table**.

## Cell Culture of 3T3-L1 Cells and Differentiation

3T3-L1 cells were obtained from ATCC and cultured to confluence in high glucose and pyruvate DMEM containing 10% calf serum by changing every 2 days according to the manufacturer’s instructions. Two days after confluence, the medium was changed to MDI differentiation medium (0.5 mM methylisobutylxanthine, 1.0 μM dexamethasone, 1.0 μg/ml bovine insulin in DMEM containing 10% fetal bovine serum). Two days later, the MDI was replaced with adipocyte maintenance medium containing 1.0 μg/ml bovine insulin and 10% fetal bovine serum in DMEM. The medium was replaced with fresh adipocyte maintenance medium every two to three days.

## RNA Interference

Three siRNAs against *CAV1* were purchased from Sigma (siRNA ID: SASI_Mm01_00141141, St. Louis, MO) and two were chosen for further use based on the knockdown efficiency. A random sequence siRNA purchased from Sigma was used as negative control (siRNA ID: SIC001). Two days after induction with MDI, cells were transfected with 50 nM siRNA using Lipofectamine® RNAiMAX Reagent (ThermoFisher Scientific, Waltham, MA) according to the recommended manufacturer’s protocol. For each siRNA, a total of six biological replicates (2 separate sets of transfection experiments, each with 3 replicates) were used and cells were collected two days after transfection to extract RNA for quantitative PCR analysis.

## Reverse Transcription and Quantitative PCR

Total RNA was purified from 3T3-L1 cells using Direct-zol RNA MiniPrep Kit with DNase I (Zymo Research, Irvine, CA) according to the manufacturer’s instructions. The first-strand cDNA were synthesized from 2 μg of total RNA with High-Capacity cDNA Reverse Transcription Kit (ThermoFisher Scientific, Waltham, MA). Amplification of each cDNA was performed with iTaq™ Universal SYBR® Green Supermix (Bio-Rad, Hercules, CA) and quantified by CFX96 qPCR System according to the protocol provided by manufacturer (Bio-Rad, Hercules, CA). The expression of the target genes was normalized to that of β-actin, which was selected as the housekeeping gene based on it stable expression during adipocyte differentiation. Linear regression was used to test for the statistical significance of each gene between control siRNA and *Cav1* siRNAs while adjusting for batch effect and siRNA differences.

**Validation of KDs and Subnetworks using TWAS and GWAS Data from HMDP**

To determine whether the subnetwork genes exhibited a global trend of having elevated association with cardiometabolic traits in HMDP, we performed enrichment test on the subnetworks of the top 15 KDs, using the MSEA module. In specific, the tissue and trait-specific TWAS data in HMDP was extracted. Genes were sorted by their association p-value and only genes with top 50% association were kept. The determination of enrichment statistics was the same as the one used for the SNPs, other than genes were used as markers directly without mapping SNPs to them.

We also took advantage of the HMDP GWAS and eQTL data in the aim of drawing causal relationship between the subnetworks and cardiometabolic traits. ~21000 mouse SNPs were reported in both the HF-HMDP and atherogenic-HMDP. The top 50% SNPs were retrieved and filtered by linkage disequilibrium threshold at 0.5. In addition, we collected eQTLs of adipose and aorta tissues from the two HMDP panels with p < 1e-5. eQTLs within the 1MB region of the transcription staring site of genes were categorized as “cis-eQTLs”, and were used to map SNPs to mouse genes. Lastly, MSEA was used to test for enrichment of association with cardiometabolic traits within the eQTLs mapped to the subnetwork genes.

**Supplemental References**

1. Shu L, Zhao Y, Kurt Z, Byars SG, Tukiainen T, Kettunen J, et al. Mergeomics: multidimensional data integration to identify pathogenic perturbations to biological systems. BMC genomics. 2016;17(1):874. doi: 10.1186/s12864-016-3198-9. PubMed PMID: 27814671; PubMed Central PMCID: PMC5097440.

2. Thorisson GA, Smith AV, Krishnan L, Stein LD. The International HapMap Project Web site. Genome research. 2005;15(11):1592-3. doi: 10.1101/gr.4413105. PubMed PMID: 16251469; PubMed Central PMCID: PMC1310647.

3. 1000 Genomes Project Consortium. An integrated map of genetic variation from 1,092 human genomes. Nature. 2012;491(7422):56-65.

4. Boyle AP, Hong EL, Hariharan M, Cheng Y, Schaub MA, Kasowski M, et al. Annotation of functional variation in personal genomes using RegulomeDB. Genome research. 2012;22(9):1790-7. doi: 10.1101/gr.137323.112. PubMed PMID: WOS:000308272800019.

5. Liberzon A, Subramanian A, Pinchback R, Thorvaldsdottir H, Tamayo P, Mesirov JP. Molecular signatures database (MSigDB) 3.0. Bioinformatics. 2011;27(12):1739-40. doi: 10.1093/bioinformatics/btr260. PubMed PMID: 21546393; PubMed Central PMCID: PMC3106198.

6. Zhu J, Zhang B, Smith EN, Drees B, Brem RB, Kruglyak L, et al. Integrating large-scale functional genomic data to dissect the complexity of yeast regulatory networks. Nature genetics. 2008;40(7):854-61. Epub 2008/06/17. doi: 10.1038/ng.167. PubMed PMID: 18552845; PubMed Central PMCID: PMC2573859.

7. Zhu J, Wiener MC, Zhang C, Fridman A, Minch E, Lum PY, et al. Increasing the power to detect causal associations by combining genotypic and expression data in segregating populations. PLoS Comput Biol. 2007;3(4):e69. Epub 2007/04/17. doi: 10.1371/journal.pcbi.0030069. PubMed PMID: 17432931; PubMed Central PMCID: PMC1851982.

8. Pearl J. Probabilistic reasoning in intelligent systems : networks of plausible inference. San Mateo, Calif.: Morgan Kaufmann Publishers; 1988. xix, 552 p. p.

9. Madigan DaY, J. Bayesian graphical models for discrete data. International Statistical Review. 1995;63:215-32.

10. Zhu J, Lum PY, Lamb J, GuhaThakurta D, Edwards SW, Thieringer R, et al. An integrative genomics approach to the reconstruction of gene networks in segregating populations. Cytogenet Genome Res. 2004;105(2-4):363-74. PubMed PMID: 15237224.

11. Greene CS, Krishnan A, Wong AK, Ricciotti E, Zelaya RA, Himmelstein DS, et al. Understanding multicellular function and disease with human tissue-specific networks. Nature genetics. 2015;47(6):569-76. doi: 10.1038/ng.3259. PubMed PMID: 25915600.

12. Pinero J, Queralt-Rosinach N, Bravo A, Deu-Pons J, Bauer-Mehren A, Baron M, et al. DisGeNET: a discovery platform for the dynamical exploration of human diseases and their genes. Database-Oxford. 2015. doi: ARTN bav02810.1093/database/bav028. PubMed PMID: WOS:000361048600001.

13. Welter D, MacArthur J, Morales J, Burdett T, Hall P, Junkins H, et al. The NHGRI GWAS Catalog, a curated resource of SNP-trait associations. Nucleic Acids Res. 2014;42(Database issue):D1001-6. doi: 10.1093/nar/gkt1229. PubMed PMID: 24316577; PubMed Central PMCID: PMC3965119.

14. Erbilgin A, Civelek M, Romanoski CE, Pan C, Hagopian R, Berliner JA, et al. Identification of CAD candidate genes in GWAS loci and their expression in vascular cells. Journal of Lipid Research. 2013;54(7):1894-905. doi: 10.1194/jlr.M037085. PubMed PMID: WOS:000320235200017.

15. Emilsson V, Thorleifsson G, Zhang B, Leonardson AS, Zink F, Zhu J, et al. Genetics of gene expression and its effect on disease. Nature. 2008;452(7186):423-8. doi: 10.1038/nature06758. PubMed PMID: 18344981.

16. Greenawalt DM, Dobrin R, Chudin E, Hatoum IJ, Suver C, Beaulaurier J, et al. A survey of the genetics of stomach, liver, and adipose gene expression from a morbidly obese cohort. Genome research. 2011;21(7):1008-16. Epub 2011/05/24. doi: 10.1101/gr.112821.110. PubMed PMID: 21602305; PubMed Central PMCID: PMC3129244.

17. Derry JM, Zhong H, Molony C, MacNeil D, Guhathakurta D, Zhang B, et al. Identification of genes and networks driving cardiovascular and metabolic phenotypes in a mouse F2 intercross. PloS one. 2010;5(12):e14319. doi: 10.1371/journal.pone.0014319. PubMed PMID: 21179467; PubMed Central PMCID: PMC3001864.

18. Wang SS, Schadt EE, Wang H, Wang X, Ingram-Drake L, Shi W, et al. Identification of pathways for atherosclerosis in mice: integration of quantitative trait locus analysis and global gene expression data. Circulation research. 2007;101(3):e11-30. doi: 10.1161/CIRCRESAHA.107.152975. PubMed PMID: 17641228.

19. Yang X, Schadt EE, Wang S, Wang H, Arnold AP, Ingram-Drake L, et al. Tissue-specific expression and regulation of sexually dimorphic genes in mice. Genome research. 2006;16(8):995-1004. Epub 2006/07/11. doi: 10.1101/gr.5217506. PubMed PMID: 16825664; PubMed Central PMCID: PMC1524872.

20. Schadt EE, Molony C, Chudin E, Hao K, Yang X, Lum PY, et al. Mapping the genetic architecture of gene expression in human liver. PLoS biology. 2008;6(5):e107. Epub 2008/05/09. doi: 10.1371/journal.pbio.0060107. PubMed PMID: 18462017; PubMed Central PMCID: PMC2365981.

21. Tu Z, Keller MP, Zhang C, Rabaglia ME, Greenawalt DM, Yang X, et al. Integrative analysis of a cross-loci regulation network identifies App as a gene regulating insulin secretion from pancreatic islets. PLoS genetics. 2012;8(12):e1003107. Epub 2012/12/14. doi: 10.1371/journal.pgen.1003107. PubMed PMID: 23236292; PubMed Central PMCID: PMC3516550.

22. Huan T, Zhang B, Wang Z, Joehanes R, Zhu J, Johnson AD, et al. A systems biology framework identifies molecular underpinnings of coronary heart disease. Arteriosclerosis, thrombosis, and vascular biology. 2013;33(6):1427-34. doi: 10.1161/ATVBAHA.112.300112. PubMed PMID: 23539213; PubMed Central PMCID: PMC3752786.

23. G. TEx Consortium. The Genotype-Tissue Expression (GTEx) project. Nature genetics. 2013;45(6):580-5. doi: 10.1038/ng.2653. PubMed PMID: 23715323; PubMed Central PMCID: PMC4010069.

24. Nica AC, Parts L, Glass D, Nisbet J, Barrett A, Sekowska M, et al. The architecture of gene regulatory variation across multiple human tissues: the MuTHER study. PLoS genetics. 2011;7(2):e1002003. doi: 10.1371/journal.pgen.1002003. PubMed PMID: 21304890; PubMed Central PMCID: PMC3033383.

25. Fehrmann RSN, Jansen RC, Veldink JH, Westra HJ, Arends D, Bonder MJ, et al. Trans-eQTLs Reveal That Independent Genetic Variants Associated with a Complex Phenotype Converge on Intermediate Genes, with a Major Role for the HLA. PLoS genetics. 2011;7(8). doi: ARTN e1002197

10.1371/journal.pgen.1002197. PubMed PMID: WOS:000294297000003.

26. Dimas AS, Deutsch S, Stranger BE, Montgomery SB, Borel C, Attar-Cohen H, et al. Common regulatory variation impacts gene expression in a cell type-dependent manner. Science. 2009;325(5945):1246-50. doi: 10.1126/science.1174148. PubMed PMID: 19644074; PubMed Central PMCID: PMC2867218.

27. Dixon AL, Liang L, Moffatt MF, Chen W, Heath S, Wong KC, et al. A genome-wide association study of global gene expression. Nature genetics. 2007;39(10):1202-7. doi: 10.1038/ng2109. PubMed PMID: 17873877.

28. Montgomery SB, Sammeth M, Gutierrez-Arcelus M, Lach RP, Ingle C, Nisbett J, et al. Transcriptome genetics using second generation sequencing in a Caucasian population. Nature. 2010;464(7289):773-U151. doi: 10.1038/nature08903. PubMed PMID: WOS:000276205000048.

29. Stranger BE, Nica AC, Forrest MS, Dimas A, Bird CP, Beazley C, et al. Population genomics of human gene expression. Nature genetics. 2007;39(10):1217-24. doi: 10.1038/ng2142. PubMed PMID: WOS:000249737400017.

30. Stranger BE, Montgomery SB, Dimas AS, Parts L, Stegle O, Ingle CE, et al. Patterns of Cis Regulatory Variation in Diverse Human Populations. PLoS genetics. 2012;8(4):272-84. doi: ARTN e100263910.1371/journal.pgen.1002639. PubMed PMID: WOS:000303441800020.

31. Duan S, Huang RS, Zhang W, Bleibel WK, Roe CA, Clark TA, et al. Genetic architecture of transcript-level variation in humans. Am J Hum Genet. 2008;82(5):1101-13. doi: 10.1016/j.ajhg.2008.03.006. PubMed PMID: WOS:000255923600009.

32. Garnier S, Truong V, Brocheton J, Zeller T, Rovital M, Wild PS, et al. Genome-wide haplotype analysis of cis expression quantitative trait loci in monocytes. PLoS genetics. 2013;9(1):e1003240. doi: 10.1371/journal.pgen.1003240. PubMed PMID: 23382694; PubMed Central PMCID: PMC3561129.
