# Supplementary material for: Shared genetic regulatory networks for cardiovascular disease and type 2 diabetes in multiple populations of diverse ethnicities in the United States
Source: PLoS Genet. 2017 Sep 28;13(9):e1007040. doi: 10.1371/journal.pgen.1007040 (PMC5634657; doi:10.1371/journal.pgen.1007040)
Supplement: S1 Table — (DOCX) [file pgen.1007040.s009.docx]

**S1 Table.** Summary of significant co-expression modules (FDR < 5%) associated with CVD or T2D

| **Trait** | **Module** | **Tissue** | **Annotation** | **Size** | **CAR+C4D/DIAGRAM** | **JHS** | **FHS** | **WHI** | **WHI** | **WHI** | ***P_meta_*** | ***FDR_meta_*** |
| --- | --- | --- | --- | --- | --- | --- | --- | --- | --- | --- | --- | --- |
|  |  |  |  |  | **Mixed** | **AA** | **EA** | **EA** | **AA** | **HA** |  |  |
| CVD | 4406 | Oth | NA | 154 | 3.32E-10 | NS | - | 4.41E-03 | 2.83E-02 | NS | 5.73E-09 | 0.00% |
| CVD | 4522 | Adp, Lv, T | REACTOME_SIGNALING_BY_FGFR_MUTANTS | 2072 | 1.03E-04 | 1.62E-02 | - | 5.53E-03 | 3.80E-02 | 2.86E-02 | 3.39E-08 | 0.00% |
| CVD | 4540 | Oth | NA | 1233 | 9.72E-04 | NS | - | 1.50E-02 | NS | 5.52E-04 | 5.07E-07 | 0.06% |
| CVD | 5242 | Adr | REACTOME_CHOLESTEROL_BIOSYNTHESIS, REACTOME_METABOLISM_OF_LIPIDS_AND_LIPOPROTEINS, LIPID_METABOLIC_PROCESS, REACTOME_FATTY_ACID_TRIACYLGLYCEROL_AND_KETONE_BODY_METABOLISM | 306 | 4.19E-06 | 4.71E-02 | - | 2.31E-02 | NS | NS | 2.64E-06 | 0.08% |
| CVD | 4087 | Adp, Dg | CARBOXYLIC_ACID_METABOLIC_PROCESS, ORGANIC_ACID_METABOLIC_PROCESS, REACTOME_METABOLISM_OF_AMINO_ACIDS_AND_DERIVATIVES, KEGG_PPAR_SIGNALING_PATHWAY | 158 | 2.34E-06 | NS | - | 8.63E-03 | NS | 2.17E-02 | 4.24E-06 | 0.09% |
| CVD | 4019 | Ly | REACTOME_TRANSMEMBRANE_TRANSPORT_OF_SMALL_MOLECULES, KEGG_GLYCINE_SERINE_AND_THREONINE_METABOLISM | 2876 | 1.89E-03 | 4.46E-02 | - | NS | NS | 6.85E-04 | 7.91E-06 | 0.20% |
| CVD | 4941 | Oth | ESTABLISHMENT_OF_LOCALIZATION, PHAGOCYTOSIS, TRANSPORT, REACTOME_TCA_CYCLE_AND_RESPIRATORY_ELECTRON_TRANSPORT | 908 | 8.97E-06 | 1.52E-02 | - | NS | NS | 3.94E-02 | 2.72E-06 | 0.21% |
| CVD | 5023 | Ly | REACTOME_TCA_CYCLE_AND_RESPIRATORY_ELECTRON_TRANSPORT, REACTOME_STRIATED_MUSCLE_CONTRACTION, REACTOME_RESPIRATORY_ELECTRON_TRANSPORT_ATP_SYNTHESIS_BY_CHEMIOSMOTIC_COUPLING_AND_HEAT_PRODUCTION_BY_UNCOUPLING_PROTEINS_, KEGG_ALZHEIMERS_DISEASE | 2890 | NS | 6.37E-05 | - | NS | 1.53E-03 | 1.50E-02 | 1.15E-05 | 0.22% |
| CVD | blue | Oth | REACTOME_CELL_CYCLE, NEGATIVE_REGULATION_OF_CELLULAR_METABOLIC_PROCESS, REACTOME_CELL_CYCLE_MITOTIC, NEGATIVE_REGULATION_OF_METABOLIC_PROCESS | 657 | 1.08E-02 | NS | - | NS | NS | 1.77E-04 | 3.85E-06 | 0.30% |
| CVD | 5329 | Adr | REACTOME_BIOLOGICAL_OXIDATIONS, KEGG_DRUG_METABOLISM_CYTOCHROME_P450, KEGG_PEROXISOME, KEGG_METABOLISM_OF_XENOBIOTICS_BY_CYTOCHROME_P450 | 1028 | NS | 2.33E-02 | - | 3.26E-02 | 5.01E-03 | 2.26E-02 | 2.21E-05 | 0.35% |
| CVD | 124 | Oth | NA | 14 | NS | 1.48E-03 | - | 7.05E-07 | NS | NS | 4.86E-06 | 0.55% |
| CVD | 4656 | Oth | CELLULAR_PROTEIN_COMPLEX_ASSEMBLY | 371 | NS | NS | - | 3.64E-03 | NS | 2.27E-04 | 8.85E-06 | 0.67% |
| CVD | 4147 | Oth | NA | 111 | 1.55E-02 | 2.06E-04 | - | 8.85E-03 | NS | NS | 5.72E-06 | 0.68% |
| CVD | 4989 | Adr | REACTOME_METABOLISM_OF_AMINO_ACIDS_AND_DERIVATIVES, REACTOME_BIOLOGICAL_OXIDATIONS, ORGANIC_ACID_METABOLIC_PROCESS, KEGG_COMPLEMENT_AND_COAGULATION_CASCADES | 453 | 1.86E-03 | 7.41E-03 | - | 3.71E-04 | NS | NS | 7.81E-05 | 0.82% |
| CVD | 5723 | Adp, Dg | NA | 656 | 9.30E-03 | NS | - | 8.98E-05 | NS | 3.06E-02 | 9.12E-06 | 1.07% |
| CVD | 4658 | Oth | REACTOME_SHC_MEDIATED_CASCADE, REACTOME_FGFR1_LIGAND_BINDING_AND_ACTIVATION, REACTOME_SIGNALING_BY_ACTIVATED_POINT_MUTANTS_OF_FGFR1, REACTOME_ACTIVATED_POINT_MUTANTS_OF_FGFR2 | 1293 | 3.38E-02 | 2.43E-03 | - | 2.39E-03 | NA | NS | 1.43E-05 | 1.12% |
| CVD | 4041 | Oth | REACTOME_DNA_REPLICATION, REACTOME_MITOTIC_M_M_G1_PHASES, REACTOME_ANTIGEN_PROCESSING_UBIQUITINATION_PROTEASOME_DEGRADATION, REACTOME_CELL_CYCLE_MITOTIC | 717 | 6.24E-05 | NS | - | NS | NS | 1.86E-02 | 1.99E-05 | 1.17% |
| CVD | 4594 | Oth | REACTOME_METABOLISM_OF_PROTEINS, REACTOME_SRP_DEPENDENT_COTRANSLATIONAL_PROTEIN_TARGETING_TO_MEMBRANE, REACTOME_TRANSLATION | 832 | 2.30E-03 | 5.17E-03 | - | NS | 1.71E-02 | NS | 2.07E-05 | 1.23% |
| CVD | 4335 | Aor | KEGG_GLIOMA, KEGG_PATHWAYS_IN_CANCER, REACTOME_SMOOTH_MUSCLE_CONTRACTION, KEGG_MAPK_SIGNALING_PATHWAY | 753 | 2.18E-03 | NS | - | NS | 8.37E-03 | NS | 6.07E-05 | 1.32% |
| CVD | 4387 | Adp, Aor | REACTOME_COMMON_PATHWAY, KEGG_COMPLEMENT_AND_COAGULATION_CASCADES, JAK_STAT_CASCADE, BIOCARTA_FIBRINOLYSIS_PATHWAY | 941 | NS | 4.46E-02 | - | NS | 6.66E-03 | 4.34E-02 | 4.67E-05 | 1.32% |
| CVD | 4561 | Aor, Dg | REACTOME_TCA_CYCLE_AND_RESPIRATORY_ELECTRON_TRANSPORT, KEGG_PARKINSONS_DISEASE, REACTOME_RESPIRATORY_ELECTRON_TRANSPORT_ATP_SYNTHESIS_BY_CHEMIOSMOTIC_COUPLING_AND_HEAT_PRODUCTION_BY_UNCOUPLING_PROTEINS_, KEGG_OXIDATIVE_PHOSPHORYLATION | 148 | 1.82E-03 | 6.03E-03 | - | 4.79E-02 | NS | NS | 1.92E-05 | 1.32% |
| CVD | 4811 | Aor | REACTOME_REGULATION_OF_WATER_BALANCE_BY_RENAL_AQUAPORINS, BIOCARTA_CREB_PATHWAY, REACTOME_NGF_SIGNALLING_VIA_TRKA_FROM_THE_PLASMA_MEMBRANE, REACTOME_AQUAPORIN_MEDIATED_TRANSPORT | 998 | 1.89E-03 | NS | - | NS | 1.97E-04 | NS | 2.79E-05 | 1.32% |
| CVD | 4062 | Mn | REACTOME_HOST_INTERACTIONS_OF_HIV_FACTORS, REACTOME_HIV_INFECTION | 107 | 2.93E-04 | 7.85E-03 | - | NS | 4.80E-05 | NS | 1.01E-05 | 1.45% |
| CVD | 4702 | Ve | NA | 1210 | NS | NA | - | 4.47E-03 | 4.26E-03 | NS | 3.93E-05 | 1.54% |
| CVD | 4037 | Oth | NA | 121 | 4.66E-04 | 7.46E-04 | - | NS | NS | NS | 3.39E-05 | 1.61% |
| CVD | 4281 | Aor | KEGG_RIBOSOME, REACTOME_SIGNALLING_BY_NGF, REACTOME_PEPTIDE_CHAIN_ELONGATION, POST_TRANSLATIONAL_PROTEIN_MODIFICATION | 503 | 3.24E-02 | NS | - | 5.10E-05 | NS | 1.35E-02 | 1.11E-04 | 1.77% |
| CVD | 159 | Ly | REACTOME_NONSENSE_MEDIATED_DECAY_ENHANCED_BY_THE_EXON_JUNCTION_COMPLEX, KEGG_RIBOSOME, REACTOME_3_UTR_MEDIATED_TRANSLATIONAL_REGULATION, REACTOME_PEPTIDE_CHAIN_ELONGATION | 1218 | NS | 6.68E-06 | - | NS | 2.61E-02 | NS | 2.48E-04 | 1.88% |
| CVD | 4363 | Oth | REACTOME_SRP_DEPENDENT_COTRANSLATIONAL_PROTEIN_TARGETING_TO_MEMBRANE, REACTOME_PEPTIDE_CHAIN_ELONGATION, REACTOME_TRANSLATION, KEGG_RIBOSOME | 796 | NS | 4.68E-06 | - | NS | NS | NS | 7.17E-05 | 1.89% |
| CVD | 5604 | Oth | RNA_METABOLIC_PROCESS | 387 | 3.69E-02 | 2.08E-03 | - | 2.95E-02 | NS | NS | 4.95E-05 | 1.89% |
| CVD | 4391 | Oth | KEGG_PPAR_SIGNALING_PATHWAY, REACTOME_GLUCOSE_METABOLISM, REACTOME_FATTY_ACYL_COA_BIOSYNTHESIS, REACTOME_TRIGLYCERIDE_BIOSYNTHESIS | 175 | 9.15E-04 | NS | - | NS | 4.34E-02 | 4.87E-02 | 6.49E-05 | 2.16% |
| CVD | 6437 | Aor | NA | 121 | NS | 1.13E-02 | - | 3.50E-02 | 6.18E-03 | NS | 1.87E-04 | 2.36% |
| CVD | 4644 | Lv | REACTOME_CELL_CYCLE_MITOTIC, REACTOME_CELL_CYCLE, REACTOME_DNA_REPLICATION, CELL_CYCLE_GO_0007049 | 264 | 1.17E-02 | NS | - | 3.51E-02 | NS | NS | 5.13E-05 | 2.40% |
| CVD | 4461 | Adp | CHROMATIN_ASSEMBLY_OR_DISASSEMBLY, CHROMATIN_ASSEMBLY, NUCLEOBASENUCLEOSIDENUCLEOTIDE_AND_NUCLEIC_ACID_METABOLIC_PROCESS, BIOPOLYMER_METABOLIC_PROCESS | 2296 | 3.80E-02 | NS | - | NS | 7.31E-03 | 1.68E-02 | 8.39E-05 | 2.47% |
| CVD | 4482 | Adp | REACTOME_PACKAGING_OF_TELOMERE_ENDS, REACTOME_MEIOTIC_SYNAPSIS, REACTOME_RNA_POL_I_PROMOTER_OPENING, REACTOME_DEPOSITION_OF_NEW_CENPA_CONTAINING_NUCLEOSOMES_AT_THE_CENTROMERE | 739 | 2.77E-03 | NS | - | NS | NS | 1.50E-02 | 5.44E-05 | 2.47% |
| CVD | 4904 | Adp | REACTOME_HIV_INFECTION, REACTOME_HOST_INTERACTIONS_OF_HIV_FACTORS, REACTOME_HIV_LIFE_CYCLE, KEGG_UBIQUITIN_MEDIATED_PROTEOLYSIS | 1184 | 6.81E-03 | 1.63E-02 | - | NS | NS | 1.13E-04 | 5.75E-05 | 2.47% |
| CVD | 5005 | Adp | REACTOME_HEMOSTASIS, REACTOME_PLATELET_ACTIVATION_SIGNALING_AND_AGGREGATION, SIGNAL_TRANSDUCTION, REACTOME_RESPONSE_TO_ELEVATED_PLATELET_CYTOSOLIC_CA2_ | 1463 | NS | 6.81E-04 | - | NS | NS | 1.11E-02 | 4.24E-05 | 2.47% |
| CVD | 6664 | Adp | NA | 40 | 2.40E-04 | 1.95E-04 | - | NS | NS | NS | 8.24E-05 | 2.47% |
| CVD | 5201 | Oth | NA | 18 | 8.69E-05 | NS | - | NS | NS | 2.77E-04 | 4.23E-05 | 2.49% |
| CVD | 4309 | Oth | REACTOME_COLLAGEN_FORMATION, REACTOME_EXTRACELLULAR_MATRIX_ORGANIZATION, KEGG_ECM_RECEPTOR_INTERACTION, KEGG_FOCAL_ADHESION | 453 | 5.61E-03 | NS | - | 4.64E-04 | NS | NS | 4.36E-05 | 2.54% |
| CVD | 4226 | Oth | REACTOME_TRANSPORT_TO_THE_GOLGI_AND_SUBSEQUENT_MODIFICATION | 568 | NS | NS | - | 3.24E-02 | 1.11E-02 | 6.40E-03 | 5.73E-05 | 2.64% |
| CVD | 4439 | Oth | NA | 90 | 1.64E-02 | NS | - | NS | 9.38E-03 | NS | 6.73E-05 | 2.64% |
| CVD | 4774 | Adr | REACTOME_RESPIRATORY_ELECTRON_TRANSPORT_ATP_SYNTHESIS_BY_CHEMIOSMOTIC_COUPLING_AND_HEAT_PRODUCTION_BY_UNCOUPLING_PROTEINS_, REGULATION_OF_GROWTH, NEGATIVE_REGULATION_OF_GROWTH, KEGG_HUNTINGTONS_DISEASE | 654 | NS | NS | - | NS | 1.20E-02 | 2.26E-04 | 3.86E-04 | 2.69% |
| CVD | 4820 | Mac | KEGG_TYPE_II_DIABETES_MELLITUS, KEGG_OTHER_GLYCAN_DEGRADATION | 366 | NS | NS | - | 1.54E-02 | 2.23E-03 | 9.64E-03 | 1.99E-05 | 2.69% |
| CVD | 4530 | Oth | NA | 166 | 5.81E-03 | NS | - | NS | NS | 2.26E-02 | 1.38E-04 | 2.71% |
| CVD | 5426 | Lv | NA | 315 | NS | NS | - | NS | NS | 3.93E-06 | 1.08E-04 | 2.78% |
| CVD | 5671 | Lv | REACTOME_PEPTIDE_CHAIN_ELONGATION, KEGG_RIBOSOME, REACTOME_3_UTR_MEDIATED_TRANSLATIONAL_REGULATION, REACTOME_INFLUENZA_VIRAL_RNA_TRANSCRIPTION_AND_REPLICATION | 1558 | 7.31E-04 | NS | - | NS | NS | 5.65E-03 | 1.19E-04 | 2.78% |
| CVD | 6861 | Lv | KEGG_OLFACTORY_TRANSDUCTION, REACTOME_OLFACTORY_SIGNALING_PATHWAY | 113 | NS | NS | - | 4.99E-04 | 1.90E-02 | 1.19E-02 | 8.24E-05 | 2.78% |
| CVD | 5422 | Oth | NA | 93 | NS | 1.28E-07 | - | NS | NS | NS | 1.47E-04 | 2.79% |
| CVD | 5473 | Oth | NA | 15 | NS | 4.14E-03 | - | 6.83E-04 | NS | 4.99E-03 | 1.31E-04 | 2.79% |
| CVD | 6392 | Oth | NA | 42 | NS | NS | - | 2.08E-04 | NS | 9.98E-04 | 1.21E-04 | 2.79% |
| CVD | 6627 | Oth | NA | 305 | 1.55E-02 | 1.69E-03 | - | NS | NS | 1.14E-02 | 1.85E-04 | 2.79% |
| CVD | 7231 | Oth | KEGG_PPAR_SIGNALING_PATHWAY, REACTOME_TRIGLYCERIDE_BIOSYNTHESIS, REACTOME_METABOLISM_OF_LIPIDS_AND_LIPOPROTEINS, REACTOME_FATTY_ACID_TRIACYLGLYCEROL_AND_KETONE_BODY_METABOLISM | 698 | 3.16E-03 | NS | - | NS | 8.79E-04 | 1.78E-03 | 1.79E-04 | 2.79% |
| CVD | 4590 | Ve | NA | 52 | 4.09E-03 | 1.52E-02 | - | NS | NA | 3.41E-03 | 1.93E-05 | 2.85% |
| CVD | 4184 | Mn | NA | 287 | NS | NS | - | 3.71E-02 | NS | 4.75E-04 | 1.45E-04 | 2.94% |
| CVD | 4295 | Mn | REACTOME_HEMOSTASIS, SYSTEM_DEVELOPMENT, REACTOME_PLATELET_ACTIVATION_SIGNALING_AND_AGGREGATION, ANATOMICAL_STRUCTURE_DEVELOPMENT | 1166 | 1.04E-03 | 1.32E-02 | - | NS | NS | 1.74E-02 | 1.15E-04 | 2.94% |
| CVD | 5039 | Mn | REACTOME_INFLUENZA_VIRAL_RNA_TRANSCRIPTION_AND_REPLICATION, REACTOME_SRP_DEPENDENT_COTRANSLATIONAL_PROTEIN_TARGETING_TO_MEMBRANE, REACTOME_METABOLISM_OF_MRNA, REACTOME_PEPTIDE_CHAIN_ELONGATION | 189 | 1.70E-02 | 1.16E-03 | - | NS | 3.42E-02 | NS | 1.44E-04 | 2.94% |
| CVD | 7236 | Oth | NA | 31 | NS | NS | - | NS | 9.38E-03 | 1.07E-04 | 1.04E-04 | 3.00% |
| CVD | 4141 | C | REACTOME_GPCR_LIGAND_BINDING, REGULATION_OF_BIOLOGICAL_QUALITY, REACTOME_FORMATION_OF_FIBRIN_CLOT_CLOTTING_CASCADE, KEGG_COMPLEMENT_AND_COAGULATION_CASCADES | 883 | 1.28E-02 | 3.64E-04 | - | NS | 4.05E-02 | NS | 1.12E-04 | 3.05% |
| CVD | 4524 | C | CARBOHYDRATE_METABOLIC_PROCESS, CELLULAR_CARBOHYDRATE_METABOLIC_PROCESS, CARBOHYDRATE_METABOLIC_PROCESS, CELLULAR_CARBOHYDRATE_METABOLIC_PROCESS | 748 | NS | 3.92E-03 | - | NS | 1.65E-02 | 3.58E-02 | 1.30E-04 | 3.05% |
| CVD | 4738 | Aor | REACTOME_CHEMOKINE_RECEPTORS_BIND_CHEMOKINES, DEFENSE_RESPONSE, RESPONSE_TO_WOUNDING, KEGG_CYTOKINE_CYTOKINE_RECEPTOR_INTERACTION | 2036 | 1.06E-02 | NS | - | NS | 1.82E-02 | 3.07E-02 | 3.11E-04 | 3.31% |
| CVD | 6626 | Lv | REACTOME_RNA_POL_I_PROMOTER_OPENING, REACTOME_AMYLOIDS, REACTOME_MEIOTIC_SYNAPSIS, REACTOME_MEIOSIS | 2780 | NS | 1.10E-02 | - | 3.14E-04 | NS | NS | 1.69E-04 | 3.40% |
| CVD | 5582 | Oth | BIOCARTA_MET_PATHWAY, INTRACELLULAR_TRANSPORT, KEGG_EPITHELIAL_CELL_SIGNALING_IN_HELICOBACTER_PYLORI_INFECTION, REACTOME_CYTOKINE_SIGNALING_IN_IMMUNE_SYSTEM | 544 | 4.81E-02 | 1.50E-02 | - | 2.42E-04 | NS | NS | 1.47E-04 | 3.45% |
| CVD | 5570 | Oth | REACTOME_DEVELOPMENTAL_BIOLOGY | 24 | 3.15E-03 | NS | - | 1.31E-03 | NS | 1.56E-02 | 2.80E-04 | 3.49% |
| CVD | 4161 | Mn | KEGG_FOCAL_ADHESION, REACTOME_TRANS_GOLGI_NETWORK_VESICLE_BUDDING, INTRACELLULAR_TRANSPORT, ESTABLISHMENT_OF_CELLULAR_LOCALIZATION | 1863 | NS | 1.33E-03 | - | 6.21E-04 | NS | NS | 2.51E-04 | 3.59% |
| CVD | 5270 | Oth | REACTOME_NONSENSE_MEDIATED_DECAY_ENHANCED_BY_THE_EXON_JUNCTION_COMPLEX, REACTOME_TRANSLATION, REACTOME_PEPTIDE_CHAIN_ELONGATION, KEGG_RIBOSOME | 1100 | 3.41E-03 | 5.02E-03 | - | NS | 2.30E-02 | NS | 3.24E-04 | 3.66% |
| CVD | 6452 | Oth | NA | 28 | 2.20E-02 | NS | - | NS | NS | 2.57E-04 | 2.18E-04 | 3.72% |
| CVD | 4033 | Adp | REACTOME_PEPTIDE_CHAIN_ELONGATION, REACTOME_TRANSLATION, KEGG_RIBOSOME, REACTOME_3_UTR_MEDIATED_TRANSLATIONAL_REGULATION | 105 | NS | NS | - | 1.04E-02 | NS | NS | 1.94E-04 | 3.81% |
| CVD | 5060 | Adp | REACTOME_PEPTIDE_CHAIN_ELONGATION, KEGG_RIBOSOME, REACTOME_INFLUENZA_VIRAL_RNA_TRANSCRIPTION_AND_REPLICATION, REACTOME_SRP_DEPENDENT_COTRANSLATIONAL_PROTEIN_TARGETING_TO_MEMBRANE | 1288 | 2.64E-02 | NS | - | NS | 9.33E-03 | 6.49E-03 | 1.86E-04 | 3.81% |
| CVD | 152 | Oth | NA | 17 | 3.74E-02 | NS | - | 1.64E-03 | 5.52E-03 | NS | 2.32E-04 | 3.82% |
| CVD | 4065 | Oth | NA | 38 | NS | NS | - | NS | 1.40E-02 | 1.51E-02 | 2.04E-04 | 3.82% |
| CVD | 4070 | Oth | REACTOME_COLLAGEN_FORMATION, REACTOME_EXTRACELLULAR_MATRIX_ORGANIZATION, KEGG_ECM_RECEPTOR_INTERACTION, REACTOME_NCAM1_INTERACTIONS | 861 | NS | 4.29E-02 | - | 9.44E-04 | NS | NS | 1.11E-04 | 3.82% |
| CVD | 4448 | Oth | REACTOME_STRIATED_MUSCLE_CONTRACTION, REACTOME_MUSCLE_CONTRACTION, STRIATED_MUSCLE_CONTRACTION_GO_0006941 | 545 | 3.57E-03 | NS | - | 2.51E-02 | 2.20E-02 | NS | 1.64E-04 | 3.82% |
| CVD | 5194 | Oth | REACTOME_RESPONSE_TO_ELEVATED_PLATELET_CYTOSOLIC_CA2_, EXCRETION, REGULATION_OF_BODY_FLUID_LEVELS | 339 | NS | NS | - | 1.20E-03 | 2.76E-02 | 1.07E-02 | 1.45E-04 | 3.82% |
| CVD | 6754 | Oth | NA | 54 | NS | 5.10E-03 | - | 3.28E-02 | NS | 2.44E-02 | 2.63E-04 | 3.82% |
| CVD | 4355 | Dg | REACTOME_METABOLISM_OF_LIPIDS_AND_LIPOPROTEINS, KEGG_FATTY_ACID_METABOLISM, REACTOME_AMYLOIDS, KEGG_PPAR_SIGNALING_PATHWAY | 1549 | 1.00E-03 | 4.42E-03 | - | 1.11E-02 | NS | 3.57E-02 | 9.51E-05 | 3.85% |
| CVD | 4519 | Dg | REACTOME_G_ALPHA1213_SIGNALLING_EVENTS | 563 | 5.95E-03 | 1.96E-03 | - | NS | 3.51E-02 | NS | 9.33E-05 | 3.85% |
| CVD | 5515 | Is | NA | 501 | NS | 2.57E-02 | - | NS | 4.13E-02 | 1.48E-04 | 5.65E-05 | 4.54% |
| CVD | 5767 | Ve | REACTOME_CELL_CYCLE, REACTOME_CELL_CYCLE_MITOTIC, REACTOME_DNA_REPLICATION, REACTOME_MITOTIC_M_M_G1_PHASES | 226 | NS | 1.12E-02 | - | NS | 9.86E-04 | 2.01E-02 | 7.70E-05 | 4.58% |
| CVD | 4758 | Oth | REACTOME_DEADENYLATION_DEPENDENT_MRNA_DECAY, KEGG_RNA_DEGRADATION, CELL_PROJECTION_BIOGENESIS | 495 | NS | 5.10E-03 | - | 3.79E-02 | NS | NS | 2.71E-04 | 4.91% |
| T2D | 5323 | Mn | NA | 38 | 8.68E-04 | NS | NS | 1.05E-03 | 2.25E-04 | NS | 1.58E-07 | 0.02% |
| T2D | 5250 | Adp, Dg, Mn | NA | 37 | 4.78E-05 | NS | NS | 3.46E-07 | 3.01E-02 | NS | 4.32E-07 | 0.03% |
| T2D | 4880 | Mn | NA | 141 | 8.96E-03 | NS | 1.18E-02 | NS | 5.06E-04 | NS | 1.61E-06 | 0.06% |
| T2D | 6872 | Mn | NA | 119 | NS | 1.26E-03 | 7.44E-03 | NS | 7.79E-03 | NS | 1.26E-06 | 0.06% |
| T2D | 4879 | Ms | NA | 376 | 3.18E-02 | NS | 5.88E-04 | 2.66E-03 | NS | 2.20E-03 | 1.19E-06 | 0.14% |
| T2D | 6533 | Mn | REACTOME_CHOLESTEROL_BIOSYNTHESIS, KEGG_STEROID_BIOSYNTHESIS, REACTOME_METABOLISM_OF_LIPIDS_AND_LIPOPROTEINS, KEGG_TERPENOID_BACKBONE_BIOSYNTHESIS | 48 | NS | 5.02E-03 | NS | NS | NA | 1.26E-06 | 1.06E-05 | 0.25% |
| T2D | 6977 | Bld | NA | 40 | 3.66E-02 | NS | 4.01E-05 | 1.81E-02 | NS | 4.05E-02 | 1.71E-06 | 0.39% |
| T2D | 6675 | Mn | REACTOME_CHOLESTEROL_BIOSYNTHESIS, REACTOME_METABOLISM_OF_LIPIDS_AND_LIPOPROTEINS, KEGG_STEROID_BIOSYNTHESIS, LIPID_METABOLIC_PROCESS | 152 | 3.72E-03 | 3.35E-02 | NS | NS | NS | 2.06E-05 | 2.56E-05 | 0.52% |
| T2D | 37 | Oth | NA | 34 | 1.94E-03 | 5.53E-03 | NS | 9.38E-04 | NS | NS | 4.95E-06 | 0.57% |
| T2D | 4302 | Adp | NA | 40 | 2.07E-03 | NS | NS | 4.05E-06 | 4.80E-03 | NS | 9.89E-06 | 0.71% |
| T2D | 6690 | Adr | KEGG_COMPLEMENT_AND_COAGULATION_CASCADES, REACTOME_P130CAS_LINKAGE_TO_MAPK_SIGNALING_FOR_INTEGRINS, REACTOME_GRB2_SOS_PROVIDES_LINKAGE_TO_MAPK_SIGNALING_FOR_INTERGRINS_, BIOCARTA_INTRINSIC_PATHWAY | 641 | 1.93E-02 | 1.01E-04 | NS | NS | 2.24E-02 | NS | 1.36E-05 | 0.86% |
| T2D | 4059 | Dg | REACTOME_SLC_MEDIATED_TRANSMEMBRANE_TRANSPORT, REACTOME_TRANSMEMBRANE_TRANSPORT_OF_SMALL_MOLECULES, CARBOXYLIC_ACID_METABOLIC_PROCESS, ORGANIC_ACID_METABOLIC_PROCESS | 51 | NS | 3.05E-02 | 5.80E-03 | 1.50E-02 | NS | NS | 1.29E-05 | 0.86% |
| T2D | 4937 | Dg | AMINO_ACID_METABOLIC_PROCESS, REACTOME_METABOLISM_OF_AMINO_ACIDS_AND_DERIVATIVES | 80 | 9.21E-03 | NS | 5.88E-03 | 1.37E-03 | NS | NS | 2.11E-05 | 0.89% |
| T2D | 5059 | Ve | REACTOME_TCA_CYCLE_AND_RESPIRATORY_ELECTRON_TRANSPORT, REACTOME_RESPIRATORY_ELECTRON_TRANSPORT_ATP_SYNTHESIS_BY_CHEMIOSMOTIC_COUPLING_AND_HEAT_PRODUCTION_BY_UNCOUPLING_PROTEINS_, REACTOME_RESPIRATORY_ELECTRON_TRANSPORT, KEGG_OXIDATIVE_PHOSPHORYLATION | 164 | 7.31E-04 | NS | 2.74E-02 | NS | 8.66E-04 | NS | 6.64E-06 | 0.95% |
| T2D | 133 | Mn | REACTOME_CHOLESTEROL_BIOSYNTHESIS, REACTOME_METABOLISM_OF_LIPIDS_AND_LIPOPROTEINS, KEGG_STEROID_BIOSYNTHESIS, KEGG_TERPENOID_BACKBONE_BIOSYNTHESIS | 57 | 2.25E-02 | NS | 1.53E-02 | NS | NS | 4.95E-05 | 6.90E-05 | 1.03% |
| T2D | 5611 | Mn | NA | 91 | NS | 5.84E-03 | NS | NS | NS | NS | 7.19E-05 | 1.03% |
| T2D | 4155 | Mn | REACTOME_CHOLESTEROL_BIOSYNTHESIS, REACTOME_GLYCOLYSIS | 197 | NS | 3.99E-02 | NS | NS | NS | 1.66E-05 | 8.49E-05 | 1.10% |
| T2D | 4820 | Ve | KEGG_TYPE_II_DIABETES_MELLITUS, KEGG_OTHER_GLYCAN_DEGRADATION | 366 | 2.55E-03 | NS | NS | NS | 4.92E-05 | NS | 1.16E-04 | 1.10% |
| T2D | 6833 | Mn | REACTOME_METABOLISM_OF_LIPIDS_AND_LIPOPROTEINS, REACTOME_FATTY_ACID_TRIACYLGLYCEROL_AND_KETONE_BODY_METABOLISM, KEGG_FATTY_ACID_METABOLISM, KEGG_PPAR_SIGNALING_PATHWAY | 93 | 9.32E-03 | NS | NS | 2.73E-02 | NS | 5.09E-04 | 1.02E-04 | 1.10% |
| T2D | 26 | Dg | REACTOME_IMMUNE_SYSTEM, IMMUNE_SYSTEM_PROCESS, REACTOME_INTERFERON_GAMMA_SIGNALING, IMMUNE_RESPONSE | 237 | 1.64E-04 | 1.06E-02 | NS | 4.01E-05 | NS | NA | 4.43E-05 | 1.24% |
| T2D | 5018 | Dg | KEGG_PPAR_SIGNALING_PATHWAY, REACTOME_GLUCOSE_METABOLISM, REACTOME_FATTY_ACYL_COA_BIOSYNTHESIS, LIPID_METABOLIC_PROCESS | 416 | 1.98E-03 | NS | NS | 2.30E-05 | 5.69E-04 | NS | 2.77E-05 | 1.24% |
| T2D | 4238 | Ve | REACTOME_TCA_CYCLE_AND_RESPIRATORY_ELECTRON_TRANSPORT, REACTOME_RESPIRATORY_ELECTRON_TRANSPORT_ATP_SYNTHESIS_BY_CHEMIOSMOTIC_COUPLING_AND_HEAT_PRODUCTION_BY_UNCOUPLING_PROTEINS_, REACTOME_RESPIRATORY_ELECTRON_TRANSPORT, KEGG_OXIDATIVE_PHOSPHORYLATION | 265 | 4.72E-02 | 4.83E-02 | NS | 4.69E-02 | 1.06E-04 | NS | 2.00E-05 | 1.34% |
| T2D | 7038 | Ve | CELLULAR_CATABOLIC_PROCESS, CATABOLIC_PROCESS | 55 | 4.96E-04 | NS | 2.72E-02 | NS | NS | 6.57E-04 | 2.79E-05 | 1.34% |
| T2D | 4956 | Oth | NA | 209 | 6.02E-07 | NS | 4.34E-03 | NS | NS | NS | 2.28E-05 | 1.35% |
| T2D | 4750 | Adp | NA | 51 | NS | 4.34E-02 | NS | 1.68E-02 | 1.58E-03 | NS | 1.87E-04 | 1.57% |
| T2D | 183 | Oth | NA | 19 | 3.62E-05 | NS | NS | 1.41E-03 | 4.31E-02 | NS | 2.12E-05 | 1.63% |
| T2D | 7249 | Mn | NA | 67 | 3.32E-02 | NS | 1.06E-03 | 2.02E-02 | NS | NS | 2.92E-04 | 2.08% |
| T2D | 5070 | Oth | NA | 20 | 7.34E-03 | NS | 4.48E-02 | NS | NS | 3.69E-03 | 1.84E-05 | 2.16% |
| T2D | 4862 | Bld | REACTOME_RESPIRATORY_ELECTRON_TRANSPORT_ATP_SYNTHESIS_BY_CHEMIOSMOTIC_COUPLING_AND_HEAT_PRODUCTION_BY_UNCOUPLING_PROTEINS_, KEGG_OXIDATIVE_PHOSPHORYLATION, REACTOME_TCA_CYCLE_AND_RESPIRATORY_ELECTRON_TRANSPORT, KEGG_HUNTINGTONS_DISEASE | 384 | 4.89E-04 | 2.60E-02 | NS | NS | 2.91E-02 | NS | 1.18E-05 | 2.16% |
| T2D | 7040 | Mn | KEGG_TYPE_I_DIABETES_MELLITUS, KEGG_ANTIGEN_PROCESSING_AND_PRESENTATION | 50 | 1.32E-02 | 3.88E-02 | NS | 1.89E-03 | 8.95E-04 | NS | 3.56E-04 | 2.42% |
| T2D | 4393 | Adp, Bld | REACTOME_PEPTIDE_CHAIN_ELONGATION, REACTOME_INFLUENZA_VIRAL_RNA_TRANSCRIPTION_AND_REPLICATION, KEGG_RIBOSOME, REACTOME_3_UTR_MEDIATED_TRANSLATIONAL_REGULATION | 160 | 4.46E-03 | NS | NS | 5.95E-03 | 8.48E-04 | NS | 1.03E-04 | 2.57% |
| T2D | 4844 | Ve | BIOPOLYMER_METABOLIC_PROCESS, CELLULAR_MACROMOLECULE_METABOLIC_PROCESS, REACTOME_GLUCOSE_METABOLISM, CELLULAR_PROTEIN_METABOLIC_PROCESS | 446 | NS | NS | NS | 1.70E-02 | 3.37E-03 | NS | 9.24E-05 | 2.76% |
| T2D | 4722 | Mn | REACTOME_METABOLISM_OF_LIPIDS_AND_LIPOPROTEINS, REACTOME_CHOLESTEROL_BIOSYNTHESIS, REACTOME_FATTY_ACYL_COA_BIOSYNTHESIS, REACTOME_TRIGLYCERIDE_BIOSYNTHESIS | 91 | 4.44E-02 | 1.50E-02 | 1.11E-02 | NS | NS | 5.22E-03 | 4.49E-04 | 2.79% |
| T2D | 5041 | Ve | NA | 52 | 1.34E-02 | 4.58E-02 | 2.03E-02 | 1.06E-02 | NS | NS | 1.18E-04 | 2.83% |
| T2D | 4721 | Mn | NA | 52 | 4.41E-03 | NS | 6.80E-03 | NS | 4.70E-02 | NS | 4.88E-04 | 2.90% |
| T2D | 6689 | Mn | KEGG_DRUG_METABOLISM_CYTOCHROME_P450, BIOCARTA_PAR1_PATHWAY, REACTOME_BIOLOGICAL_OXIDATIONS, REACTOME_GLYCOSPHINGOLIPID_METABOLISM | 2151 | 7.75E-03 | 2.92E-03 | NS | NS | 1.16E-02 | NS | 5.20E-04 | 2.97% |
| T2D | 4524 | Bld | CARBOHYDRATE_METABOLIC_PROCESS, CELLULAR_CARBOHYDRATE_METABOLIC_PROCESS, CARBOHYDRATE_METABOLIC_PROCESS, CELLULAR_CARBOHYDRATE_METABOLIC_PROCESS | 748 | NS | NS | 1.46E-04 | NS | 7.32E-04 | NS | 6.37E-05 | 3.00% |
| T2D | 4428 | Mn | REACTOME_METABOLISM_OF_RNA, REACTOME_PEPTIDE_CHAIN_ELONGATION, KEGG_RIBOSOME, REACTOME_NONSENSE_MEDIATED_DECAY_ENHANCED_BY_THE_EXON_JUNCTION_COMPLEX | 713 | 3.51E-04 | NS | NS | 6.96E-03 | 2.71E-03 | NS | 4.15E-05 | 3.11% |
| T2D | 4566 | Adp, Mac | REACTOME_METABOLISM_OF_LIPIDS_AND_LIPOPROTEINS, KEGG_PPAR_SIGNALING_PATHWAY, REACTOME_FATTY_ACID_TRIACYLGLYCEROL_AND_KETONE_BODY_METABOLISM, REACTOME_TRIGLYCERIDE_BIOSYNTHESIS | 569 | 1.35E-02 | 3.61E-03 | 3.47E-02 | NS | 1.53E-03 | NS | 2.33E-05 | 3.13% |
| T2D | 4998 | Adp | REACTOME_IMMUNE_SYSTEM, REACTOME_INTERFERON_GAMMA_SIGNALING, REACTOME_INTERFERON_SIGNALING, REACTOME_CYTOKINE_SIGNALING_IN_IMMUNE_SYSTEM | 107 | 9.96E-03 | NS | 5.18E-03 | 5.38E-06 | NS | NS | 1.58E-04 | 3.18% |
| T2D | 4669 | Mn | REACTOME_GLUCOSE_METABOLISM, KEGG_PEROXISOME, REACTOME_FATTY_ACID_TRIACYLGLYCEROL_AND_KETONE_BODY_METABOLISM, REACTOME_METABOLISM_OF_LIPIDS_AND_LIPOPROTEINS | 401 | 3.65E-02 | NS | 5.04E-03 | NS | 2.71E-02 | 1.37E-03 | 5.90E-04 | 3.21% |
| T2D | 4651 | Mn | REACTOME_TCA_CYCLE_AND_RESPIRATORY_ELECTRON_TRANSPORT, REACTOME_RESPIRATORY_ELECTRON_TRANSPORT_ATP_SYNTHESIS_BY_CHEMIOSMOTIC_COUPLING_AND_HEAT_PRODUCTION_BY_UNCOUPLING_PROTEINS_, REACTOME_RESPIRATORY_ELECTRON_TRANSPORT, KEGG_OXIDATIVE_PHOSPHORYLATION | 319 | 4.95E-03 | NS | NS | 2.64E-04 | 7.33E-03 | NS | 6.81E-04 | 3.36% |
| T2D | 4822 | Bld | SYNAPTIC_TRANSMISSION, TRANSMISSION_OF_NERVE_IMPULSE, KEGG_OXIDATIVE_PHOSPHORYLATION, REACTOME_TRANSMISSION_ACROSS_CHEMICAL_SYNAPSES | 1426 | 9.71E-04 | NS | NS | NS | 1.18E-03 | 1.21E-02 | 9.66E-05 | 3.54% |
| T2D | 5539 | Dg | REACTOME_BIOLOGICAL_OXIDATIONS, KEGG_DRUG_METABOLISM_CYTOCHROME_P450, KEGG_METABOLISM_OF_XENOBIOTICS_BY_CYTOCHROME_P450, REACTOME_METABOLISM_OF_AMINO_ACIDS_AND_DERIVATIVES | 232 | 3.62E-02 | NS | 2.39E-02 | NS | NS | NS | 8.89E-05 | 3.54% |
| T2D | 5685 | Oth | NA | 13 | NS | NS | 1.63E-04 | NS | 5.47E-03 | NS | 1.08E-04 | 3.64% |
| T2D | brown | Mn | BIOCARTA_AHSP_PATHWAY, REACTOME_METABOLISM_OF_PORPHYRINS, REACTOME_MHC_CLASS_II_ANTIGEN_PRESENTATION, COFACTOR_BIOSYNTHETIC_PROCESS | 480 | 2.55E-02 | NS | NS | 2.81E-02 | NS | 1.91E-03 | 1.01E-04 | 3.64% |
| T2D | 4094 | Mn | KEGG_ALZHEIMERS_DISEASE, KEGG_HUNTINGTONS_DISEASE, KEGG_PARKINSONS_DISEASE, KEGG_OXIDATIVE_PHOSPHORYLATION | 65 | NS | NS | NS | NS | 3.11E-02 | NS | 9.31E-04 | 3.80% |
| T2D | 4127 | Mn | KEGG_METABOLISM_OF_XENOBIOTICS_BY_CYTOCHROME_P450, KEGG_DRUG_METABOLISM_CYTOCHROME_P450, REACTOME_BIOLOGICAL_OXIDATIONS, REACTOME_METABOLISM_OF_AMINO_ACIDS_AND_DERIVATIVES | 948 | NS | NS | NS | NS | 3.18E-02 | 5.97E-03 | 9.58E-04 | 3.80% |
| T2D | 4865 | Mn | NA | 122 | 4.43E-02 | NS | NS | 3.44E-02 | 5.33E-03 | NS | 9.42E-04 | 3.80% |
| T2D | 5055 | Mn | REACTOME_STRIATED_MUSCLE_CONTRACTION, REACTOME_MUSCLE_CONTRACTION, REACTOME_INTERFERON_ALPHA_BETA_SIGNALING, TUBE_DEVELOPMENT | 988 | NS | NS | NS | NS | 7.26E-05 | 1.43E-02 | 9.08E-04 | 3.80% |
| T2D | 6630 | Mn | REACTOME_INTERFERON_ALPHA_BETA_SIGNALING, REACTOME_IMMUNE_SYSTEM, REACTOME_CYTOKINE_SIGNALING_IN_IMMUNE_SYSTEM, REACTOME_INTERFERON_SIGNALING | 73 | NS | NS | NS | 8.00E-04 | 1.33E-02 | NS | 9.36E-04 | 3.80% |
| T2D | 4555 | Oth | REACTOME_ADP_SIGNALLING_THROUGH_P2RY1, REACTOME_SIGNAL_AMPLIFICATION, REACTOME_THROMBIN_SIGNALLING_THROUGH_PROTEINASE_ACTIVATED_RECEPTORS_PARS | 206 | 8.57E-04 | NS | NS | NS | 2.30E-02 | NS | 9.91E-05 | 3.92% |
| T2D | 4723 | Ve | INTRACELLULAR_TRANSPORT, REACTOME_HIV_LIFE_CYCLE, TRANSPORT, REACTOME_HIV_INFECTION | 2836 | NS | NS | 4.13E-02 | 1.32E-04 | 4.33E-02 | NS | 3.72E-04 | 4.11% |
| T2D | 5219 | Oth | NA | 13 | 7.64E-04 | 3.24E-02 | NS | 1.41E-02 | NS | NS | 1.59E-04 | 4.38% |

***** The annotation refers to the top 4 functional categories enriched in the co-expression modules (Bonferroni-corrected p< 0.05 based on Fisher’s exact test, number of direct overlapping genes > 5). Adp – adipose tissue; Adr – adrenal gland; Aor – Aorta; Art – artery; Bld – Blood; C – Coronary artery; Dg –digestive tract; Is – Islet; Hy – hypothalamus; Lv – liver; Ly – lymphocyte; Ms – muscle; Mac – Macrophage; Mn – Monocyte; Oth – Modules mapped by distance or ENCODE or all eQTLs combined; T – thyroid gland; Ve – vascular endothelium.
