# Supplementary material for: Shared genetic regulatory networks for cardiovascular disease and type 2 diabetes in multiple populations of diverse ethnicities in the United States
Source: PLoS Genet. 2017 Sep 28;13(9):e1007040. doi: 10.1371/journal.pgen.1007040 (PMC5634657; doi:10.1371/journal.pgen.1007040)
Supplement: S2 Table — (DOCX) [file pgen.1007040.s010.docx]

**S2 Table.** Functional annotation terms of the significant co-expression modules

| **Co-expression Module** | **Annotation Term** | **Fold Enrichment** | **Pvalue** | **FDR** | **Size of Co-expression Module** | **Size of Annotation Term** | **Number of Overlapping Genes** | **Overlap Ratio (Module)** | **Overlap Ratio (Annotation term)** |
| --- | --- | --- | --- | --- | --- | --- | --- | --- | --- |
| 4238 | REACTOME RESPIRATORY ELECTRON TRANSPORT ATP SYNTHESIS BY CHEMIOSMOTIC COUPLING AND HEAT PRODUCTION BY UNCOUPLING PROTEINS | 39.92 | 1.94E-57 | 1.84E-54 | 238 | 80 | 40 | 0.17 | 0.50 |
| 4651 | REACTOME RESPIRATORY ELECTRON TRANSPORT ATP SYNTHESIS BY CHEMIOSMOTIC COUPLING AND HEAT PRODUCTION BY UNCOUPLING PROTEINS | 31.83 | 1.02E-51 | 9.67E-49 | 291 | 80 | 39 | 0.13 | 0.49 |
| 26 | REACTOME IMMUNE SYSTEM | 7.39 | 6.84E-45 | 1.30E-41 | 210 | 894 | 73 | 0.35 | 0.08 |
| 4033 | REACTOME SRP DEPENDENT COTRANSLATIONAL PROTEIN TARGETING TO MEMBRANE | 51.08 | 1.04E-41 | 3.30E-39 | 93 | 108 | 27 | 0.29 | 0.25 |
| 6533 | REACTOME CHOLESTEROL BIOSYNTHESIS | 308.46 | 8.99E-40 | 1.71E-36 | 42 | 22 | 15 | 0.36 | 0.68 |
| 133 | REACTOME CHOLESTEROL BIOSYNTHESIS | 239.91 | 1.14E-37 | 2.16E-34 | 54 | 22 | 15 | 0.28 | 0.68 |
| 4989 | REACTOME METABOLISM OF AMINO ACIDS AND DERIVATIVES | 11.33 | 2.46E-37 | 4.68E-34 | 402 | 196 | 47 | 0.12 | 0.24 |
| 5059 | REACTOME RESPIRATORY ELECTRON TRANSPORT ATP SYNTHESIS BY CHEMIOSMOTIC COUPLING AND HEAT PRODUCTION BY UNCOUPLING PROTEINS | 40.36 | 2.80E-37 | 2.66E-34 | 153 | 80 | 26 | 0.17 | 0.33 |
| 5671 | REACTOME SRP DEPENDENT COTRANSLATIONAL PROTEIN TARGETING TO MEMBRANE | 6.87 | 5.04E-34 | 1.60E-31 | 1382 | 108 | 54 | 0.04 | 0.50 |
| 4989 | REACTOME BIOLOGICAL OXIDATIONS | 13.56 | 5.00E-33 | 3.25E-30 | 402 | 129 | 37 | 0.09 | 0.29 |
| 6675 | REACTOME CHOLESTEROL BIOSYNTHESIS | 95.30 | 9.77E-33 | 1.86E-29 | 145 | 22 | 16 | 0.11 | 0.73 |
| 6675 | REACTOME METABOLISM OF LIPIDS AND LIPOPROTEINS | 10.90 | 8.14E-31 | 7.74E-28 | 145 | 469 | 39 | 0.27 | 0.08 |
| 4651 | KEGG CITRATE CYCLE TCA CYCLE | 41.35 | 6.84E-30 | 1.44E-27 | 291 | 30 | 19 | 0.07 | 0.63 |
| 4238 | KEGG CITRATE CYCLE TCA CYCLE | 47.90 | 1.68E-29 | 3.56E-27 | 238 | 30 | 18 | 0.08 | 0.60 |
| 133 | REACTOME METABOLISM OF LIPIDS AND LIPOPROTEINS | 19.51 | 1.97E-29 | 1.87E-26 | 54 | 469 | 26 | 0.48 | 0.06 |
| 4998 | REACTOME IMMUNE SYSTEM | 8.73 | 2.89E-28 | 5.49E-25 | 95 | 894 | 39 | 0.41 | 0.04 |
| 26 | REACTOME CYTOKINE SIGNALING IN IMMUNE SYSTEM | 11.74 | 8.53E-28 | 3.24E-25 | 210 | 262 | 34 | 0.16 | 0.13 |
| 4561 | REACTOME RESPIRATORY ELECTRON TRANSPORT ATP SYNTHESIS BY CHEMIOSMOTIC COUPLING AND HEAT PRODUCTION BY UNCOUPLING PROTEINS | 37.83 | 9.09E-26 | 5.76E-23 | 113 | 80 | 18 | 0.16 | 0.23 |
| 4393 | REACTOME SRP DEPENDENT COTRANSLATIONAL PROTEIN TARGETING TO MEMBRANE | 25.31 | 2.13E-24 | 5.78E-22 | 139 | 108 | 20 | 0.14 | 0.19 |
| 4644 | REACTOME CELL CYCLE MITOTIC | 8.57 | 1.14E-23 | 2.17E-20 | 252 | 308 | 35 | 0.14 | 0.11 |
| 6533 | REACTOME METABOLISM OF LIPIDS AND LIPOPROTEINS | 19.29 | 3.72E-23 | 2.36E-20 | 42 | 469 | 20 | 0.48 | 0.04 |
| 5767 | REACTOME CELL CYCLE MITOTIC | 9.14 | 1.03E-22 | 9.76E-20 | 216 | 308 | 32 | 0.15 | 0.10 |
| 4989 | REACTOME METABOLISM OF LIPIDS AND LIPOPROTEINS | 5.04 | 4.39E-22 | 8.35E-20 | 402 | 469 | 50 | 0.12 | 0.11 |
| 5242 | REACTOME CHOLESTEROL BIOSYNTHESIS | 40.83 | 3.66E-21 | 6.97E-18 | 275 | 22 | 13 | 0.05 | 0.59 |
| 4998 | REACTOME CYTOKINE SIGNALING IN IMMUNE SYSTEM | 16.03 | 4.05E-21 | 1.93E-18 | 95 | 262 | 21 | 0.22 | 0.08 |
| 4127 | REACTOME BIOLOGICAL OXIDATIONS | 6.19 | 4.18E-20 | 2.65E-17 | 857 | 129 | 36 | 0.04 | 0.28 |
| 5242 | REACTOME METABOLISM OF LIPIDS AND LIPOPROTEINS | 5.75 | 1.12E-19 | 1.06E-16 | 275 | 469 | 39 | 0.14 | 0.08 |
| 4309 | REACTOME COLLAGEN FORMATION | 15.76 | 1.19E-19 | 2.27E-16 | 402 | 57 | 19 | 0.05 | 0.33 |
| 4651 | KEGG FATTY ACID METABOLISM | 23.32 | 7.06E-19 | 1.12E-16 | 291 | 42 | 15 | 0.05 | 0.36 |
| 5059 | REACTOME METABOLISM OF LIPIDS AND LIPOPROTEINS | 7.68 | 8.97E-19 | 1.90E-16 | 153 | 469 | 29 | 0.19 | 0.06 |
| 4238 | KEGG FATTY ACID METABOLISM | 26.61 | 1.37E-18 | 1.87E-16 | 238 | 42 | 14 | 0.06 | 0.33 |
| 4566 | REACTOME METABOLISM OF LIPIDS AND LIPOPROTEINS | 4.08 | 2.21E-18 | 2.55E-15 | 506 | 469 | 51 | 0.10 | 0.11 |
| 4566 | KEGG PPAR SIGNALING PATHWAY | 11.43 | 2.68E-18 | 2.55E-15 | 506 | 69 | 21 | 0.04 | 0.30 |
| 5059 | KEGG CITRATE CYCLE TCA CYCLE | 45.54 | 3.66E-18 | 6.33E-16 | 153 | 30 | 11 | 0.07 | 0.37 |
| 6833 | REACTOME METABOLISM OF LIPIDS AND LIPOPROTEINS | 10.61 | 3.90E-18 | 7.43E-15 | 84 | 469 | 22 | 0.26 | 0.05 |
| 5539 | REACTOME BIOLOGICAL OXIDATIONS | 13.85 | 6.76E-18 | 1.28E-14 | 202 | 129 | 19 | 0.09 | 0.15 |
| 5059 | KEGG FATTY ACID METABOLISM | 35.48 | 7.44E-18 | 1.18E-15 | 153 | 42 | 12 | 0.08 | 0.29 |
| 4127 | REACTOME METABOLISM OF AMINO ACIDS AND DERIVATIVES | 4.52 | 1.06E-16 | 5.04E-14 | 857 | 196 | 40 | 0.05 | 0.20 |
| 4651 | REACTOME METABOLISM OF LIPIDS AND LIPOPROTEINS | 5.01 | 2.53E-16 | 3.00E-14 | 291 | 469 | 36 | 0.12 | 0.08 |
| 7231 | KEGG PPAR SIGNALING PATHWAY | 9.18 | 2.70E-16 | 5.14E-13 | 630 | 69 | 21 | 0.03 | 0.30 |
| 4989 | KEGG GLYCINE SERINE AND THREONINE METABOLISM | 19.82 | 5.50E-16 | 4.65E-14 | 402 | 31 | 13 | 0.03 | 0.42 |
| 7231 | REACTOME TRIGLYCERIDE BIOSYNTHESIS | 12.70 | 8.61E-16 | 8.19E-13 | 630 | 38 | 16 | 0.03 | 0.42 |
| 4989 | KEGG PPAR SIGNALING PATHWAY | 11.65 | 3.17E-15 | 2.32E-13 | 402 | 69 | 17 | 0.04 | 0.25 |
| 4070 | REACTOME COLLAGEN FORMATION | 8.48 | 3.57E-15 | 6.80E-12 | 786 | 57 | 20 | 0.03 | 0.35 |
| 7231 | REACTOME METABOLISM OF LIPIDS AND LIPOPROTEINS | 3.34 | 5.38E-15 | 3.41E-12 | 630 | 469 | 52 | 0.08 | 0.11 |
| 4722 | REACTOME METABOLISM OF LIPIDS AND LIPOPROTEINS | 9.27 | 9.19E-15 | 1.75E-11 | 83 | 469 | 19 | 0.23 | 0.04 |
| 4087 | REACTOME METABOLISM OF AMINO ACIDS AND DERIVATIVES | 11.37 | 1.06E-14 | 6.70E-12 | 145 | 196 | 17 | 0.12 | 0.09 |
| 4561 | REACTOME TRIGLYCERIDE BIOSYNTHESIS | 39.83 | 1.52E-14 | 3.60E-12 | 113 | 38 | 9 | 0.08 | 0.24 |
| 4566 | REACTOME TRIGLYCERIDE BIOSYNTHESIS | 13.83 | 1.72E-14 | 8.20E-12 | 506 | 38 | 14 | 0.03 | 0.37 |
| 4989 | REACTOME SYNTHESIS OF BILE ACIDS AND BILE SALTS | 24.88 | 2.16E-14 | 1.41E-12 | 402 | 19 | 10 | 0.02 | 0.53 |
| 5060 | REACTOME SRP DEPENDENT COTRANSLATIONAL PROTEIN TARGETING TO MEMBRANE | 4.72 | 2.40E-14 | 1.14E-11 | 1155 | 108 | 31 | 0.03 | 0.29 |
| 5329 | REACTOME BIOLOGICAL OXIDATIONS | 4.96 | 2.94E-14 | 5.59E-11 | 890 | 129 | 30 | 0.03 | 0.23 |
| 4238 | REACTOME METABOLISM OF LIPIDS AND LIPOPROTEINS | 5.11 | 3.69E-14 | 2.92E-12 | 238 | 469 | 30 | 0.13 | 0.06 |
| 5039 | REACTOME SRP DEPENDENT COTRANSLATIONAL PROTEIN TARGETING TO MEMBRANE | 14.49 | 4.30E-14 | 4.09E-11 | 170 | 108 | 14 | 0.08 | 0.13 |
| 6630 | REACTOME IMMUNE SYSTEM | 7.20 | 1.00E-13 | 9.52E-11 | 59 | 894 | 20 | 0.34 | 0.02 |
| 133 | REACTOME TRIGLYCERIDE BIOSYNTHESIS | 64.82 | 1.13E-13 | 3.08E-11 | 54 | 38 | 7 | 0.13 | 0.18 |
| 4309 | KEGG ECM RECEPTOR INTERACTION | 9.57 | 1.31E-13 | 8.31E-11 | 402 | 84 | 17 | 0.04 | 0.20 |
| 4087 | KEGG PPAR SIGNALING PATHWAY | 20.89 | 1.49E-13 | 7.10E-11 | 145 | 69 | 11 | 0.08 | 0.16 |
| 6833 | KEGG FATTY ACID METABOLISM | 43.09 | 1.64E-13 | 1.04E-10 | 84 | 42 | 8 | 0.10 | 0.19 |
| 4989 | REACTOME LIPID DIGESTION MOBILIZATION AND TRANSPORT | 13.65 | 2.64E-13 | 1.44E-11 | 402 | 45 | 13 | 0.03 | 0.29 |
| 5059 | KEGG PPAR SIGNALING PATHWAY | 19.80 | 2.85E-13 | 2.26E-11 | 153 | 69 | 11 | 0.07 | 0.16 |
| 4561 | KEGG BIOSYNTHESIS OF UNSATURATED FATTY ACIDS | 53.50 | 3.63E-13 | 6.90E-11 | 113 | 22 | 7 | 0.06 | 0.32 |
| 6833 | KEGG PPAR SIGNALING PATHWAY | 29.50 | 4.51E-13 | 2.15E-10 | 84 | 69 | 9 | 0.11 | 0.13 |
| 7231 | KEGG PEROXISOME | 7.35 | 6.27E-13 | 2.39E-10 | 630 | 78 | 19 | 0.03 | 0.24 |
| 6630 | REACTOME CYTOKINE SIGNALING IN IMMUNE SYSTEM | 14.75 | 1.11E-12 | 6.13E-10 | 59 | 262 | 12 | 0.20 | 0.05 |
| 4363 | REACTOME SRP DEPENDENT COTRANSLATIONAL PROTEIN TARGETING TO MEMBRANE | 5.64 | 1.58E-12 | 3.01E-09 | 718 | 108 | 23 | 0.03 | 0.21 |
| 6833 | KEGG PEROXISOME | 26.10 | 1.62E-12 | 6.16E-10 | 84 | 78 | 9 | 0.11 | 0.12 |
| 5023 | REACTOME RESPIRATORY ELECTRON TRANSPORT ATP SYNTHESIS BY CHEMIOSMOTIC COUPLING AND HEAT PRODUCTION BY UNCOUPLING PROTEINS | 3.29 | 1.81E-12 | 1.15E-09 | 2602 | 80 | 36 | 0.01 | 0.45 |
| 4722 | REACTOME CHOLESTEROL BIOSYNTHESIS | 62.43 | 3.79E-12 | 3.60E-09 | 83 | 22 | 6 | 0.07 | 0.27 |
| 5018 | KEGG PPAR SIGNALING PATHWAY | 10.34 | 4.06E-12 | 7.71E-09 | 373 | 69 | 14 | 0.04 | 0.20 |
| 4355 | REACTOME METABOLISM OF LIPIDS AND LIPOPROTEINS | 2.26 | 4.29E-12 | 8.17E-09 | 1383 | 469 | 77 | 0.06 | 0.16 |
| 4989 | REACTOME TRYPTOPHAN CATABOLISM | 30.08 | 5.85E-12 | 2.71E-10 | 402 | 11 | 7 | 0.02 | 0.64 |
| 4428 | REACTOME SRP DEPENDENT COTRANSLATIONAL PROTEIN TARGETING TO MEMBRANE | 5.75 | 9.60E-12 | 2.03E-09 | 642 | 108 | 21 | 0.03 | 0.19 |
| 4059 | REACTOME TRANSMEMBRANE TRANSPORT OF SMALL MOLECULES | 11.86 | 1.28E-11 | 1.21E-08 | 47 | 409 | 12 | 0.26 | 0.03 |
| 4155 | REACTOME CHOLESTEROL BIOSYNTHESIS | 33.59 | 1.58E-11 | 3.01E-08 | 180 | 22 | 7 | 0.04 | 0.32 |
| 4355 | KEGG FATTY ACID METABOLISM | 5.89 | 1.88E-11 | 1.79E-08 | 1383 | 42 | 18 | 0.01 | 0.43 |
| 4989 | KEGG ALANINE ASPARTATE AND GLUTAMATE METABOLISM | 14.77 | 2.87E-11 | 1.21E-09 | 402 | 32 | 10 | 0.02 | 0.31 |
| 5539 | REACTOME METABOLISM OF AMINO ACIDS AND DERIVATIVES | 7.68 | 3.48E-11 | 1.65E-08 | 202 | 196 | 16 | 0.08 | 0.08 |
| 4561 | REACTOME METABOLISM OF LIPIDS AND LIPOPROTEINS | 6.45 | 3.95E-11 | 6.26E-09 | 113 | 469 | 18 | 0.16 | 0.04 |
| 5329 | KEGG PEROXISOME | 5.47 | 4.15E-11 | 2.63E-08 | 890 | 78 | 20 | 0.02 | 0.26 |
| 4989 | KEGG PEROXISOME | 8.48 | 7.62E-11 | 2.84E-09 | 402 | 78 | 14 | 0.03 | 0.18 |
| 4087 | KEGG ALANINE ASPARTATE AND GLUTAMATE METABOLISM | 28.67 | 8.53E-11 | 2.03E-08 | 145 | 32 | 7 | 0.05 | 0.22 |
| 5023 | REACTOME MUSCLE CONTRACTION | 3.81 | 8.67E-11 | 3.30E-08 | 2602 | 46 | 24 | 0.01 | 0.52 |
| 4094 | REACTOME RESPIRATORY ELECTRON TRANSPORT ATP SYNTHESIS BY CHEMIOSMOTIC COUPLING AND HEAT PRODUCTION BY UNCOUPLING PROTEINS | 29.17 | 9.65E-11 | 3.67E-08 | 57 | 80 | 7 | 0.12 | 0.09 |
| 5242 | REACTOME TRIGLYCERIDE BIOSYNTHESIS | 16.36 | 1.13E-10 | 2.69E-08 | 275 | 38 | 9 | 0.03 | 0.24 |
| 26 | KEGG PRIMARY IMMUNODEFICIENCY | 20.68 | 1.14E-10 | 6.38E-09 | 210 | 35 | 8 | 0.04 | 0.23 |
| 4070 | KEGG ECM RECEPTOR INTERACTION | 5.47 | 1.49E-10 | 9.48E-08 | 786 | 84 | 19 | 0.02 | 0.23 |
| 4904 | REACTOME HIV INFECTION | 3.22 | 1.76E-10 | 3.35E-07 | 1075 | 192 | 35 | 0.03 | 0.18 |
| 4566 | KEGG PEROXISOME | 7.22 | 1.90E-10 | 4.52E-08 | 506 | 78 | 15 | 0.03 | 0.19 |
| 4651 | REACTOME TRIGLYCERIDE BIOSYNTHESIS | 15.46 | 1.97E-10 | 9.14E-09 | 291 | 38 | 9 | 0.03 | 0.24 |
| 159 | REACTOME SRP DEPENDENT COTRANSLATIONAL PROTEIN TARGETING TO MEMBRANE | 4.07 | 2.50E-10 | 3.97E-08 | 1082 | 108 | 25 | 0.02 | 0.23 |
| 4355 | KEGG PPAR SIGNALING PATHWAY | 4.38 | 2.60E-10 | 1.21E-07 | 1383 | 69 | 22 | 0.02 | 0.32 |
| 4391 | KEGG PPAR SIGNALING PATHWAY | 15.49 | 3.00E-10 | 5.70E-07 | 160 | 69 | 9 | 0.06 | 0.13 |
| 4087 | REACTOME TRIGLYCERIDE BIOSYNTHESIS | 24.14 | 3.82E-10 | 7.53E-08 | 145 | 38 | 7 | 0.05 | 0.18 |
| 6675 | REACTOME TRIGLYCERIDE BIOSYNTHESIS | 24.14 | 3.82E-10 | 1.04E-07 | 145 | 38 | 7 | 0.05 | 0.18 |
| 4561 | KEGG CITRATE CYCLE TCA CYCLE | 33.63 | 3.96E-10 | 4.71E-08 | 113 | 30 | 6 | 0.05 | 0.20 |
| 4087 | REACTOME METABOLISM OF LIPIDS AND LIPOPROTEINS | 5.31 | 4.81E-10 | 8.32E-08 | 145 | 469 | 19 | 0.13 | 0.04 |
| 5005 | REACTOME HEMOSTASIS | 2.16 | 5.32E-10 | 1.01E-06 | 1327 | 451 | 68 | 0.05 | 0.15 |
| 4651 | KEGG PPAR SIGNALING PATHWAY | 10.41 | 5.38E-10 | 2.44E-08 | 291 | 69 | 11 | 0.04 | 0.16 |
| 5059 | REACTOME TRIGLYCERIDE BIOSYNTHESIS | 22.88 | 5.86E-10 | 3.18E-08 | 153 | 38 | 7 | 0.05 | 0.18 |
| 5059 | KEGG PEROXISOME | 14.33 | 6.71E-10 | 3.55E-08 | 153 | 78 | 9 | 0.06 | 0.12 |
| 4238 | KEGG PPAR SIGNALING PATHWAY | 11.57 | 9.10E-10 | 4.02E-08 | 238 | 69 | 10 | 0.04 | 0.14 |
| 26 | REACTOME PEPTIDE LIGAND BINDING RECEPTORS | 7.08 | 1.43E-09 | 6.82E-08 | 210 | 179 | 14 | 0.07 | 0.08 |
| 4355 | REACTOME BIOLOGICAL OXIDATIONS | 3.20 | 2.26E-09 | 6.15E-07 | 1383 | 129 | 30 | 0.02 | 0.23 |
| 4566 | KEGG BIOSYNTHESIS OF UNSATURATED FATTY ACIDS | 13.66 | 2.29E-09 | 4.36E-07 | 506 | 22 | 8 | 0.02 | 0.36 |
| 4651 | KEGG PEROXISOME | 9.21 | 2.34E-09 | 9.69E-08 | 291 | 78 | 11 | 0.04 | 0.14 |
| 26 | KEGG SYSTEMIC LUPUS ERYTHEMATOSUS | 8.10 | 3.09E-09 | 1.31E-07 | 210 | 134 | 12 | 0.06 | 0.09 |
| 4391 | REACTOME GLUCOSE METABOLISM | 14.84 | 3.15E-09 | 2.99E-06 | 160 | 64 | 8 | 0.05 | 0.13 |
| 4238 | KEGG PEROXISOME | 10.24 | 3.51E-09 | 1.49E-07 | 238 | 78 | 10 | 0.04 | 0.13 |
| 5018 | REACTOME GLUCOSE METABOLISM | 8.76 | 3.60E-09 | 3.42E-06 | 373 | 64 | 11 | 0.03 | 0.17 |
| 4989 | REACTOME TRANSMEMBRANE TRANSPORT OF SMALL MOLECULES | 3.35 | 3.77E-09 | 9.96E-08 | 402 | 409 | 29 | 0.07 | 0.07 |
| 4087 | REACTOME BIOLOGICAL OXIDATIONS | 10.16 | 4.30E-09 | 6.29E-07 | 145 | 129 | 10 | 0.07 | 0.08 |
| 4723 | REACTOME HIV INFECTION | 2.15 | 4.39E-09 | 2.09E-06 | 2582 | 192 | 56 | 0.02 | 0.29 |
| 5767 | REACTOME KINESINS | 21.99 | 6.54E-09 | 4.61E-07 | 216 | 24 | 6 | 0.03 | 0.25 |
| 4295 | REACTOME HEMOSTASIS | 2.24 | 6.99E-09 | 1.33E-05 | 1035 | 451 | 55 | 0.05 | 0.12 |
| brown | REACTOME METABOLISM OF PORPHYRINS | 18.78 | 7.82E-09 | 7.44E-06 | 467 | 13 | 6 | 0.01 | 0.46 |
| 4989 | KEGG BIOSYNTHESIS OF UNSATURATED FATTY ACIDS | 15.04 | 9.24E-09 | 2.34E-07 | 402 | 22 | 7 | 0.02 | 0.32 |
| 5767 | REACTOME CYCLIN A B1 ASSOCIATED EVENTS DURING G2 M TRANSITION | 29.32 | 9.25E-09 | 6.28E-07 | 216 | 15 | 5 | 0.02 | 0.33 |
| 4566 | REACTOME GLUCOSE METABOLISM | 7.04 | 1.10E-08 | 1.75E-06 | 506 | 64 | 12 | 0.02 | 0.19 |
| 4087 | KEGG BIOSYNTHESIS OF UNSATURATED FATTY ACIDS | 29.78 | 1.20E-08 | 1.43E-06 | 145 | 22 | 5 | 0.03 | 0.23 |
| 4722 | REACTOME TRIGLYCERIDE BIOSYNTHESIS | 30.12 | 1.43E-08 | 6.79E-06 | 83 | 38 | 5 | 0.06 | 0.13 |
| 7231 | KEGG BIOSYNTHESIS OF UNSATURATED FATTY ACIDS | 10.97 | 1.55E-08 | 3.27E-06 | 630 | 22 | 8 | 0.01 | 0.36 |
| 4448 | REACTOME MUSCLE CONTRACTION | 8.52 | 1.59E-08 | 1.26E-05 | 485 | 46 | 10 | 0.02 | 0.22 |
| 4989 | REACTOME GAMMA CARBOXYLATION TRANSPORT AND AMINO TERMINAL CLEAVAGE OF PROTEINS | 23.63 | 1.69E-08 | 4.07E-07 | 402 | 10 | 5 | 0.01 | 0.50 |
| 5242 | KEGG BIOSYNTHESIS OF UNSATURATED FATTY ACIDS | 18.84 | 1.75E-08 | 2.55E-06 | 275 | 22 | 6 | 0.02 | 0.27 |
| 4989 | KEGG PANTOTHENATE AND COA BIOSYNTHESIS | 17.72 | 1.75E-08 | 4.10E-07 | 402 | 16 | 6 | 0.01 | 0.38 |
| 5329 | KEGG BIOSYNTHESIS OF UNSATURATED FATTY ACIDS | 8.73 | 1.87E-08 | 3.56E-06 | 890 | 22 | 9 | 0.01 | 0.41 |
| 4998 | KEGG PRIMARY IMMUNODEFICIENCY | 28.57 | 1.92E-08 | 8.31E-07 | 95 | 35 | 5 | 0.05 | 0.14 |
| 4391 | REACTOME TRIGLYCERIDE BIOSYNTHESIS | 18.75 | 2.67E-08 | 1.27E-05 | 160 | 38 | 6 | 0.04 | 0.16 |
| 4059 | REACTOME BIOLOGICAL OXIDATIONS | 18.80 | 2.83E-08 | 5.39E-06 | 47 | 129 | 6 | 0.13 | 0.05 |
| 5270 | REACTOME SRP DEPENDENT COTRANSLATIONAL PROTEIN TARGETING TO MEMBRANE | 3.74 | 2.98E-08 | 9.45E-06 | 987 | 108 | 21 | 0.02 | 0.19 |
| 4594 | REACTOME SRP DEPENDENT COTRANSLATIONAL PROTEIN TARGETING TO MEMBRANE | 4.25 | 3.79E-08 | 3.61E-05 | 746 | 108 | 18 | 0.02 | 0.17 |
| 5767 | KEGG ECM RECEPTOR INTERACTION | 9.43 | 3.88E-08 | 2.17E-06 | 216 | 84 | 9 | 0.04 | 0.11 |
| 4041 | REACTOME CELL CYCLE MITOTIC | 2.87 | 4.42E-08 | 2.10E-05 | 666 | 308 | 31 | 0.05 | 0.10 |
| 5059 | REACTOME GLUCOSE METABOLISM | 13.58 | 4.44E-08 | 1.92E-06 | 153 | 64 | 7 | 0.05 | 0.11 |
| 5329 | REACTOME METABOLISM OF LIPIDS AND LIPOPROTEINS | 2.23 | 5.19E-08 | 7.59E-06 | 890 | 469 | 49 | 0.06 | 0.10 |
| 5539 | REACTOME TRANSMEMBRANE TRANSPORT OF SMALL MOLECULES | 4.14 | 8.26E-08 | 1.21E-05 | 202 | 409 | 18 | 0.09 | 0.04 |
| 5055 | REACTOME MUSCLE CONTRACTION | 5.70 | 8.88E-08 | 8.44E-05 | 870 | 46 | 12 | 0.01 | 0.26 |
| 4391 | REACTOME METABOLISM OF LIPIDS AND LIPOPROTEINS | 4.30 | 9.08E-08 | 3.45E-05 | 160 | 469 | 17 | 0.11 | 0.04 |
| 4937 | REACTOME METABOLISM OF AMINO ACIDS AND DERIVATIVES | 10.07 | 9.69E-08 | 3.55E-05 | 77 | 196 | 8 | 0.10 | 0.04 |
| 5329 | REACTOME TRANSMEMBRANE TRANSPORT OF SMALL MOLECULES | 2.30 | 9.74E-08 | 1.23E-05 | 890 | 409 | 44 | 0.05 | 0.11 |
| 4059 | REACTOME TRANSPORT OF INORGANIC CATIONS ANIONS AND AMINO ACIDS OLIGOPEPTIDES | 21.74 | 1.07E-07 | 1.43E-05 | 47 | 93 | 5 | 0.11 | 0.05 |
| 159 | REACTOME SPRY REGULATION OF FGF SIGNALING | 9.46 | 1.07E-07 | 1.46E-05 | 1082 | 13 | 7 | 0.01 | 0.54 |
| 4989 | REACTOME REGULATION OF GENE EXPRESSION IN BETA CELLS | 14.18 | 1.10E-07 | 2.30E-06 | 402 | 20 | 6 | 0.01 | 0.30 |
| 4937 | REACTOME COLLAGEN FORMATION | 21.65 | 1.12E-07 | 3.55E-05 | 77 | 57 | 5 | 0.06 | 0.09 |
| 4094 | REACTOME BIOLOGICAL OXIDATIONS | 15.50 | 1.13E-07 | 1.34E-05 | 57 | 129 | 6 | 0.11 | 0.05 |
| 4094 | KEGG PEROXISOME | 21.37 | 1.21E-07 | 1.35E-05 | 57 | 78 | 5 | 0.09 | 0.06 |
| 4041 | REACTOME ASSOCIATION OF TRIC CCT WITH TARGET PROTEINS DURING BIOSYNTHESIS | 8.78 | 1.38E-07 | 5.27E-05 | 666 | 26 | 8 | 0.01 | 0.31 |
| 5329 | REACTOME METABOLISM OF AMINO ACIDS AND DERIVATIVES | 2.94 | 1.41E-07 | 1.58E-05 | 890 | 196 | 27 | 0.03 | 0.14 |
| 7231 | REACTOME GLUCOSE METABOLISM | 5.66 | 1.44E-07 | 2.28E-05 | 630 | 64 | 12 | 0.02 | 0.19 |
| 5539 | REACTOME METABOLISM OF LIPIDS AND LIPOPROTEINS | 3.81 | 1.49E-07 | 1.84E-05 | 202 | 469 | 19 | 0.09 | 0.04 |
| 26 | REACTOME HEMOSTASIS | 3.81 | 1.50E-07 | 4.39E-06 | 210 | 451 | 19 | 0.09 | 0.04 |
| 4561 | KEGG FATTY ACID METABOLISM | 20.02 | 1.71E-07 | 1.27E-05 | 113 | 42 | 5 | 0.04 | 0.12 |
| 4561 | KEGG PPAR SIGNALING PATHWAY | 14.62 | 1.74E-07 | 1.27E-05 | 113 | 69 | 6 | 0.05 | 0.09 |
| 5018 | REACTOME METABOLISM OF LIPIDS AND LIPOPROTEINS | 2.93 | 1.99E-07 | 5.10E-05 | 373 | 469 | 27 | 0.07 | 0.06 |
| blue | REACTOME CELL CYCLE MITOTIC | 2.78 | 2.25E-07 | 1.30E-04 | 643 | 308 | 29 | 0.05 | 0.09 |
| 5329 | REACTOME TRANSPORT OF INORGANIC CATIONS ANIONS AND AMINO ACIDS OLIGOPEPTIDES | 3.90 | 2.61E-07 | 2.62E-05 | 890 | 93 | 17 | 0.02 | 0.18 |
| 4998 | REACTOME PEPTIDE LIGAND BINDING RECEPTORS | 8.94 | 2.80E-07 | 9.34E-06 | 95 | 179 | 8 | 0.08 | 0.04 |
| 4041 | REACTOME HIV INFECTION | 3.27 | 2.85E-07 | 7.75E-05 | 666 | 192 | 22 | 0.03 | 0.11 |
| 5023 | KEGG CITRATE CYCLE TCA CYCLE | 3.65 | 3.15E-07 | 3.36E-05 | 2602 | 30 | 15 | 0.01 | 0.50 |
| 26 | REACTOME INNATE IMMUNE SYSTEM | 4.73 | 3.16E-07 | 8.24E-06 | 210 | 268 | 14 | 0.07 | 0.05 |
| 4127 | KEGG PEROXISOME | 4.26 | 3.18E-07 | 3.36E-05 | 857 | 78 | 15 | 0.02 | 0.19 |
| 5242 | REACTOME GLUCOSE METABOLISM | 8.64 | 3.36E-07 | 3.76E-05 | 275 | 64 | 8 | 0.03 | 0.13 |
| 4127 | KEGG ABC TRANSPORTERS | 5.54 | 3.64E-07 | 3.65E-05 | 857 | 44 | 11 | 0.01 | 0.25 |
| 6861 | KEGG OLFACTORY TRANSDUCTION | 5.86 | 3.70E-07 | 7.04E-04 | 92 | 388 | 11 | 0.12 | 0.03 |
| 5023 | KEGG TGF BETA SIGNALING PATHWAY | 2.49 | 4.07E-07 | 3.68E-05 | 2602 | 85 | 29 | 0.01 | 0.34 |
| 4669 | REACTOME GLUCOSE METABOLISM | 7.20 | 4.23E-07 | 8.05E-04 | 371 | 64 | 9 | 0.02 | 0.14 |
| 159 | REACTOME RESPIRATORY ELECTRON TRANSPORT ATP SYNTHESIS BY CHEMIOSMOTIC COUPLING AND HEAT PRODUCTION BY UNCOUPLING PROTEINS | 3.73 | 4.24E-07 | 5.04E-05 | 1082 | 80 | 17 | 0.02 | 0.21 |
| 5582 | REACTOME CYTOKINE SIGNALING IN IMMUNE SYSTEM | 3.20 | 4.76E-07 | 2.26E-04 | 499 | 262 | 22 | 0.04 | 0.08 |
| 4355 | KEGG PEROXISOME | 3.35 | 5.07E-07 | 4.59E-05 | 1383 | 78 | 19 | 0.01 | 0.24 |
| 4758 | REACTOME DEADENYLATION DEPENDENT MRNA DECAY | 8.02 | 5.13E-07 | 9.76E-04 | 431 | 44 | 8 | 0.02 | 0.18 |
| 4155 | REACTOME METABOLISM OF LIPIDS AND LIPOPROTEINS | 3.83 | 5.39E-07 | 2.05E-04 | 180 | 469 | 17 | 0.09 | 0.04 |
| 4651 | REACTOME GLUCOSE METABOLISM | 8.16 | 5.40E-07 | 1.94E-05 | 291 | 64 | 8 | 0.03 | 0.13 |
| 4644 | REACTOME KINESINS | 15.71 | 5.65E-07 | 3.16E-05 | 252 | 24 | 5 | 0.02 | 0.21 |
| 4161 | REACTOME DEVELOPMENTAL BIOLOGY | 1.85 | 5.65E-07 | 2.15E-04 | 1682 | 384 | 63 | 0.04 | 0.16 |
| 5018 | REACTOME TRIGLYCERIDE BIOSYNTHESIS | 9.38 | 5.98E-07 | 9.48E-05 | 373 | 38 | 7 | 0.02 | 0.18 |
| 4723 | REACTOME CTNNB1 PHOSPHORYLATION CASCADE | 4.60 | 6.46E-07 | 8.11E-05 | 2582 | 16 | 10 | 0.00 | 0.63 |
| 5242 | KEGG PPAR SIGNALING PATHWAY | 8.01 | 6.49E-07 | 6.17E-05 | 275 | 69 | 8 | 0.03 | 0.12 |
| 4070 | KEGG PPAR SIGNALING PATHWAY | 4.55 | 7.13E-07 | 8.37E-05 | 786 | 69 | 13 | 0.02 | 0.19 |
| 4651 | KEGG BIOSYNTHESIS OF UNSATURATED FATTY ACIDS | 14.84 | 7.44E-07 | 2.57E-05 | 291 | 22 | 5 | 0.02 | 0.23 |
| 4141 | REACTOME HEMOSTASIS | 2.20 | 8.00E-07 | 3.04E-04 | 784 | 451 | 41 | 0.05 | 0.09 |
| 7231 | KEGG FATTY ACID METABOLISM | 6.46 | 8.43E-07 | 9.43E-05 | 630 | 42 | 9 | 0.01 | 0.21 |
| 4651 | REACTOME MITOCHONDRIAL PROTEIN IMPORT | 8.96 | 9.89E-07 | 3.30E-05 | 291 | 51 | 7 | 0.02 | 0.14 |
| 4723 | REACTOME MEMBRANE TRAFFICKING | 2.16 | 1.03E-06 | 1.08E-04 | 2582 | 126 | 37 | 0.01 | 0.29 |
| 5767 | REACTOME HEMOSTASIS | 3.51 | 1.04E-06 | 3.74E-05 | 216 | 451 | 18 | 0.08 | 0.04 |
| 4141 | REACTOME TRANSMEMBRANE TRANSPORT OF SMALL MOLECULES | 2.25 | 1.10E-06 | 3.29E-04 | 784 | 409 | 38 | 0.05 | 0.09 |
| 4937 | KEGG ECM RECEPTOR INTERACTION | 14.69 | 1.15E-06 | 1.82E-04 | 77 | 84 | 5 | 0.06 | 0.06 |
| 4355 | KEGG BIOSYNTHESIS OF UNSATURATED FATTY ACIDS | 5.62 | 1.16E-06 | 8.83E-05 | 1383 | 22 | 9 | 0.01 | 0.41 |
| 4094 | REACTOME METABOLISM OF LIPIDS AND LIPOPROTEINS | 6.40 | 1.17E-06 | 8.54E-05 | 57 | 469 | 9 | 0.16 | 0.02 |
| 4844 | REACTOME GLUCOSE METABOLISM | 6.39 | 1.25E-06 | 7.93E-04 | 418 | 64 | 9 | 0.02 | 0.14 |
| 4566 | KEGG FATTY ACID METABOLISM | 7.15 | 1.28E-06 | 1.35E-04 | 506 | 42 | 8 | 0.02 | 0.19 |
| 7231 | REACTOME BIOLOGICAL OXIDATIONS | 3.74 | 1.28E-06 | 1.27E-04 | 630 | 129 | 16 | 0.03 | 0.12 |
| 4723 | REACTOME IMMUNE SYSTEM | 1.40 | 1.39E-06 | 1.38E-04 | 2582 | 894 | 170 | 0.07 | 0.19 |
| 4862 | REACTOME RESPIRATORY ELECTRON TRANSPORT ATP SYNTHESIS BY CHEMIOSMOTIC COUPLING AND HEAT PRODUCTION BY UNCOUPLING PROTEINS | 6.36 | 1.44E-06 | 2.74E-03 | 336 | 80 | 9 | 0.03 | 0.11 |
| 4070 | REACTOME CHOLESTEROL BIOSYNTHESIS | 7.69 | 1.58E-06 | 1.50E-04 | 786 | 22 | 7 | 0.01 | 0.32 |
| 4127 | REACTOME METABOLISM OF LIPIDS AND LIPOPROTEINS | 2.08 | 1.61E-06 | 1.24E-04 | 857 | 469 | 44 | 0.05 | 0.09 |
| 5539 | KEGG PEROXISOME | 8.44 | 1.76E-06 | 1.52E-04 | 202 | 78 | 7 | 0.03 | 0.09 |
| 7231 | KEGG SULFUR METABOLISM | 11.60 | 1.82E-06 | 1.65E-04 | 630 | 13 | 5 | 0.01 | 0.38 |
| 4723 | REACTOME CELL CYCLE MITOTIC | 1.70 | 1.99E-06 | 1.54E-04 | 2582 | 308 | 71 | 0.03 | 0.23 |
| 6675 | KEGG PEROXISOME | 10.08 | 2.19E-06 | 2.45E-04 | 145 | 78 | 6 | 0.04 | 0.08 |
| 5539 | KEGG ARACHIDONIC ACID METABOLISM | 9.90 | 2.33E-06 | 1.85E-04 | 202 | 57 | 6 | 0.03 | 0.11 |
| 4989 | KEGG FATTY ACID METABOLISM | 7.88 | 2.35E-06 | 4.35E-05 | 402 | 42 | 7 | 0.02 | 0.17 |
| 4566 | REACTOME LIPID DIGESTION MOBILIZATION AND TRANSPORT | 6.68 | 2.37E-06 | 2.25E-04 | 506 | 45 | 8 | 0.02 | 0.18 |
| 4155 | REACTOME GLUCOSE METABOLISM | 9.90 | 2.40E-06 | 7.61E-04 | 180 | 64 | 6 | 0.03 | 0.09 |
| 4309 | REACTOME DEVELOPMENTAL BIOLOGY | 2.83 | 2.51E-06 | 3.98E-04 | 402 | 384 | 23 | 0.06 | 0.06 |
| 5329 | KEGG FATTY ACID METABOLISM | 5.08 | 2.52E-06 | 1.71E-04 | 890 | 42 | 10 | 0.01 | 0.24 |
| 4669 | KEGG PEROXISOME | 5.91 | 2.76E-06 | 2.62E-03 | 371 | 78 | 9 | 0.02 | 0.12 |
| 4989 | KEGG ARACHIDONIC ACID METABOLISM | 6.63 | 2.85E-06 | 5.11E-05 | 402 | 57 | 8 | 0.02 | 0.14 |
| 4295 | REACTOME NEURONAL SYSTEM | 2.26 | 2.89E-06 | 7.86E-04 | 1035 | 276 | 34 | 0.03 | 0.12 |
| 5767 | KEGG SYSTEMIC LUPUS ERYTHEMATOSUS | 5.91 | 3.13E-06 | 1.04E-04 | 216 | 134 | 9 | 0.04 | 0.07 |
| 5018 | KEGG BIOSYNTHESIS OF UNSATURATED FATTY ACIDS | 11.58 | 3.14E-06 | 3.99E-04 | 373 | 22 | 5 | 0.01 | 0.23 |
| 4238 | REACTOME MITOCHONDRIAL PROTEIN IMPORT | 9.39 | 3.21E-06 | 1.15E-04 | 238 | 51 | 6 | 0.03 | 0.12 |
| 5059 | REACTOME MITOCHONDRIAL PROTEIN IMPORT | 12.18 | 3.30E-06 | 1.10E-04 | 153 | 51 | 5 | 0.03 | 0.10 |
| 4482 | REACTOME MUSCLE CONTRACTION | 5.60 | 3.31E-06 | 4.85E-04 | 664 | 46 | 9 | 0.01 | 0.20 |
| 4989 | KEGG ABC TRANSPORTERS | 7.52 | 3.41E-06 | 5.89E-05 | 402 | 44 | 7 | 0.02 | 0.16 |
| 5005 | REACTOME METABOLISM OF LIPIDS AND LIPOPROTEINS | 1.80 | 3.85E-06 | 3.33E-04 | 1327 | 469 | 59 | 0.04 | 0.13 |
| 4141 | REACTOME NEURONAL SYSTEM | 2.46 | 4.03E-06 | 6.60E-04 | 784 | 276 | 28 | 0.04 | 0.10 |
| 4989 | REACTOME HEMOSTASIS | 2.62 | 4.22E-06 | 7.16E-05 | 402 | 451 | 25 | 0.06 | 0.06 |
| 159 | REACTOME HIV INFECTION | 2.47 | 4.39E-06 | 3.21E-04 | 1082 | 192 | 27 | 0.02 | 0.14 |
| 4482 | KEGG SYSTEMIC LUPUS ERYTHEMATOSUS | 3.42 | 4.42E-06 | 6.00E-04 | 664 | 134 | 16 | 0.02 | 0.12 |
| 4127 | REACTOME GLUCOSE METABOLISM | 4.16 | 4.53E-06 | 2.97E-04 | 857 | 64 | 12 | 0.01 | 0.19 |
| 5005 | REACTOME SRP DEPENDENT COTRANSLATIONAL PROTEIN TARGETING TO MEMBRANE | 2.78 | 4.54E-06 | 3.76E-04 | 1327 | 108 | 21 | 0.02 | 0.19 |
| 4019 | REACTOME TRANSMEMBRANE TRANSPORT OF SMALL MOLECULES | 1.58 | 4.63E-06 | 8.80E-03 | 2565 | 409 | 87 | 0.03 | 0.21 |
| 4041 | REACTOME IMMUNE SYSTEM | 1.82 | 4.73E-06 | 6.43E-04 | 666 | 894 | 57 | 0.09 | 0.06 |
| 4141 | REACTOME PEPTIDE LIGAND BINDING RECEPTORS | 2.84 | 4.83E-06 | 6.60E-04 | 784 | 179 | 21 | 0.03 | 0.12 |
| 4998 | KEGG SYSTEMIC LUPUS ERYTHEMATOSUS | 8.96 | 4.89E-06 | 1.15E-04 | 95 | 134 | 6 | 0.06 | 0.04 |
| 5570 | REACTOME DEVELOPMENTAL BIOLOGY | 10.76 | 4.94E-06 | 2.35E-03 | 23 | 384 | 5 | 0.22 | 0.01 |
| 5582 | REACTOME IMMUNE SYSTEM | 1.96 | 4.97E-06 | 1.05E-03 | 499 | 894 | 46 | 0.09 | 0.05 |
| 5005 | REACTOME ACTIVATED NOTCH1 TRANSMITS SIGNAL TO THE NUCLEUS | 4.96 | 5.03E-06 | 3.99E-04 | 1327 | 26 | 9 | 0.01 | 0.35 |
| 4904 | REACTOME CELL CYCLE MITOTIC | 2.12 | 5.24E-06 | 6.64E-04 | 1075 | 308 | 37 | 0.03 | 0.12 |
| 4461 | REACTOME ENOS ACTIVATION AND REGULATION | 4.53 | 5.86E-06 | 2.23E-03 | 1989 | 19 | 9 | 0.00 | 0.47 |
| 4127 | KEGG GLYCINE SERINE AND THREONINE METABOLISM | 5.72 | 6.09E-06 | 3.62E-04 | 857 | 31 | 8 | 0.01 | 0.26 |
| 4669 | REACTOME METABOLISM OF LIPIDS AND LIPOPROTEINS | 2.62 | 6.23E-06 | 2.96E-03 | 371 | 469 | 24 | 0.06 | 0.05 |
| 4062 | REACTOME HIV INFECTION | 7.14 | 6.30E-06 | 3.51E-03 | 97 | 192 | 7 | 0.07 | 0.04 |
| 4033 | REACTOME INNATE IMMUNE SYSTEM | 6.10 | 6.61E-06 | 4.06E-04 | 93 | 268 | 8 | 0.09 | 0.03 |
| 6675 | REACTOME BIOLOGICAL OXIDATIONS | 7.11 | 6.63E-06 | 5.74E-04 | 145 | 129 | 7 | 0.05 | 0.05 |
| 4482 | REACTOME CHOLESTEROL BIOSYNTHESIS | 7.80 | 6.65E-06 | 8.43E-04 | 664 | 22 | 6 | 0.01 | 0.27 |
| 4774 | REACTOME RESPIRATORY ELECTRON TRANSPORT ATP SYNTHESIS BY CHEMIOSMOTIC COUPLING AND HEAT PRODUCTION BY UNCOUPLING PROTEINS | 4.40 | 6.75E-06 | 1.06E-02 | 594 | 80 | 11 | 0.02 | 0.14 |
| 6626 | REACTOME HEMOSTASIS | 1.55 | 6.93E-06 | 6.94E-04 | 2445 | 451 | 90 | 0.04 | 0.20 |
| 6689 | REACTOME BIOLOGICAL OXIDATIONS | 2.26 | 7.52E-06 | 4.06E-03 | 1890 | 129 | 29 | 0.02 | 0.22 |
| 4281 | REACTOME SRP DEPENDENT COTRANSLATIONAL PROTEIN TARGETING TO MEMBRANE | 4.78 | 7.98E-06 | 2.53E-03 | 368 | 108 | 10 | 0.03 | 0.09 |
| 4295 | REACTOME DOPAMINE NEUROTRANSMITTER RELEASE CYCLE | 8.34 | 9.39E-06 | 1.79E-03 | 1035 | 11 | 5 | 0.00 | 0.45 |
| 4161 | REACTOME METABOLISM OF LIPIDS AND LIPOPROTEINS | 1.66 | 9.88E-06 | 1.28E-03 | 1682 | 469 | 69 | 0.04 | 0.15 |
| 6626 | KEGG SYSTEMIC LUPUS ERYTHEMATOSUS | 2.03 | 1.02E-05 | 9.23E-04 | 2445 | 134 | 35 | 0.01 | 0.26 |
| 4070 | KEGG SYSTEMIC LUPUS ERYTHEMATOSUS | 3.07 | 1.05E-05 | 5.87E-04 | 786 | 134 | 17 | 0.02 | 0.13 |
| 4141 | REACTOME CLASS B 2 SECRETIN FAMILY RECEPTORS | 3.66 | 1.06E-05 | 8.67E-04 | 784 | 86 | 13 | 0.02 | 0.15 |
| 4309 | REACTOME A TETRASACCHARIDE LINKER SEQUENCE IS REQUIRED FOR GAG SYNTHESIS | 9.45 | 1.09E-05 | 1.43E-03 | 402 | 25 | 5 | 0.01 | 0.20 |
| 4127 | REACTOME SYNTHESIS OF BILE ACIDS AND BILE SALTS | 7.00 | 1.16E-05 | 6.30E-04 | 857 | 19 | 6 | 0.01 | 0.32 |
| 4033 | REACTOME HEMOSTASIS | 4.53 | 1.26E-05 | 7.06E-04 | 93 | 451 | 10 | 0.11 | 0.02 |
| 4127 | KEGG FATTY ACID METABOLISM | 4.75 | 1.31E-05 | 6.71E-04 | 857 | 42 | 9 | 0.01 | 0.21 |
| 5582 | REACTOME HIV INFECTION | 3.17 | 1.32E-05 | 1.64E-03 | 499 | 192 | 16 | 0.03 | 0.08 |
| 5329 | KEGG SULFUR METABOLISM | 8.21 | 1.34E-05 | 6.55E-04 | 890 | 13 | 5 | 0.01 | 0.38 |
| 4226 | REACTOME TRANSPORT TO THE GOLGI AND SUBSEQUENT MODIFICATION | 9.14 | 1.50E-05 | 1.43E-02 | 315 | 33 | 5 | 0.02 | 0.15 |
| 4355 | REACTOME MUSCLE CONTRACTION | 3.58 | 1.60E-05 | 8.44E-04 | 1383 | 46 | 12 | 0.01 | 0.26 |
| 5329 | KEGG PPAR SIGNALING PATHWAY | 3.71 | 1.62E-05 | 7.15E-04 | 890 | 69 | 12 | 0.01 | 0.17 |
| 5023 | REACTOME METABOLISM OF LIPIDS AND LIPOPROTEINS | 1.49 | 1.62E-05 | 6.31E-04 | 2602 | 469 | 96 | 0.04 | 0.20 |
| 5023 | REACTOME TRIGLYCERIDE BIOSYNTHESIS | 2.88 | 1.63E-05 | 6.31E-04 | 2602 | 38 | 15 | 0.01 | 0.39 |
| 5023 | KEGG FATTY ACID METABOLISM | 2.78 | 1.67E-05 | 6.34E-04 | 2602 | 42 | 16 | 0.01 | 0.38 |
| 5242 | KEGG PEROXISOME | 6.20 | 1.70E-05 | 8.73E-04 | 275 | 78 | 7 | 0.03 | 0.09 |
| 4127 | REACTOME REGULATION OF GENE EXPRESSION IN BETA CELLS | 6.65 | 1.71E-05 | 8.36E-04 | 857 | 20 | 6 | 0.01 | 0.30 |
| 4019 | KEGG GLYCINE SERINE AND THREONINE METABOLISM | 3.11 | 1.78E-05 | 1.25E-02 | 2565 | 31 | 13 | 0.01 | 0.42 |
| 4738 | REACTOME TRYPTOPHAN CATABOLISM | 5.64 | 1.81E-05 | 2.29E-03 | 1836 | 11 | 6 | 0.00 | 0.55 |
| 6630 | REACTOME INNATE IMMUNE SYSTEM | 7.21 | 1.87E-05 | 1.55E-03 | 59 | 268 | 6 | 0.10 | 0.02 |
| 6675 | REACTOME SRP DEPENDENT COTRANSLATIONAL PROTEIN TARGETING TO MEMBRANE | 7.28 | 1.91E-05 | 1.35E-03 | 145 | 108 | 6 | 0.04 | 0.06 |
| 4822 | REACTOME NEURONAL SYSTEM | 2.00 | 1.97E-05 | 5.15E-03 | 1276 | 276 | 37 | 0.03 | 0.13 |
| brown | REACTOME KINESINS | 8.48 | 1.97E-05 | 2.61E-03 | 467 | 24 | 5 | 0.01 | 0.21 |
| 4281 | REACTOME P75 NTR RECEPTOR MEDIATED SIGNALLING | 5.23 | 2.17E-05 | 5.04E-03 | 368 | 79 | 8 | 0.02 | 0.10 |
| 5018 | KEGG CITRATE CYCLE TCA CYCLE | 8.49 | 2.19E-05 | 1.98E-03 | 373 | 30 | 5 | 0.01 | 0.17 |
| 4141 | KEGG PPAR SIGNALING PATHWAY | 3.86 | 2.27E-05 | 1.23E-03 | 784 | 69 | 11 | 0.01 | 0.16 |
| 4141 | REACTOME BIOLOGICAL OXIDATIONS | 3.01 | 2.29E-05 | 1.23E-03 | 784 | 129 | 16 | 0.02 | 0.12 |
| 4070 | REACTOME BIOLOGICAL OXIDATIONS | 3.00 | 2.36E-05 | 1.21E-03 | 786 | 129 | 16 | 0.02 | 0.12 |
| 4355 | REACTOME GAMMA CARBOXYLATION TRANSPORT AND AMINO TERMINAL CLEAVAGE OF PROTEINS | 6.87 | 2.39E-05 | 1.11E-03 | 1383 | 10 | 5 | 0.00 | 0.50 |
| 4811 | KEGG VASOPRESSIN REGULATED WATER REABSORPTION | 4.97 | 2.50E-05 | 2.91E-03 | 695 | 44 | 8 | 0.01 | 0.18 |
| 5018 | REACTOME LIPID DIGESTION MOBILIZATION AND TRANSPORT | 6.79 | 2.53E-05 | 2.08E-03 | 373 | 45 | 6 | 0.02 | 0.13 |
| 5582 | REACTOME HEMOSTASIS | 2.28 | 2.55E-05 | 2.02E-03 | 499 | 451 | 27 | 0.05 | 0.06 |
| 4335 | KEGG P53 SIGNALING PATHWAY | 4.10 | 2.73E-05 | 2.73E-03 | 691 | 67 | 10 | 0.01 | 0.15 |
| 4522 | REACTOME SIGNALING BY FGFR MUTANTS | 3.15 | 2.83E-05 | 2.82E-02 | 1821 | 43 | 13 | 0.01 | 0.30 |
| 159 | REACTOME MEMBRANE TRAFFICKING | 2.65 | 2.88E-05 | 1.44E-03 | 1082 | 126 | 19 | 0.02 | 0.15 |
