# Supplementary material for: Shared genetic regulatory networks for cardiovascular disease and type 2 diabetes in multiple populations of diverse ethnicities in the United States
Source: PLoS Genet. 2017 Sep 28;13(9):e1007040. doi: 10.1371/journal.pgen.1007040 (PMC5634657; doi:10.1371/journal.pgen.1007040)
Supplement: S3 Table — (DOCX) [file pgen.1007040.s011.docx]

**S3 Table**. List of previously reported genes associated with CVD, T2D, and intermediate metabolic traits related to CVD, T2D from DisGeNET and GWAS Catalog.

| TRAIT | TYPE | GENE |
| --- | --- | --- |
| CAD | GWAS Catalog | ABO, ACAD10, ADAMTS7, ADORA2A, ALDH2, AP3D1, APOA1, APOA4, APOA5, APOC1, APOC3, APOE, ATP2B1, BTNL2, C12orf51, C6orf10, CDKN2A, CDKN2B, CELSR2, CHD3, CNNM2, COL4A1, COL4A2, CXCL12, CYP17A1, DOT1L, EDNRA, GGCX, GUCY1A3, HDAC9, HHIPL1, HLA-C, HSP90B1, IL6R, KCNE2, KIAA1462, KSR2, LDLR, LIPA, LPA, MIA3, MORF4L1, MRAS, MRPS6, MTAP, MYL2, NOS3, NT5C2, PDGFD, PEMT, PHACTR1, PITX2, PLCL2, PLG, PPAP2B, PSRC1, RAI1, RASD1, SF3A2, SH2B3, SLC22A3, SMAD3, SMARCA4, SMG6, SORT1, SRR, STK32B, TCF21, TTC32, VAMP5, VAMP8, WDR12, WDR35, ZC3HC1, ZEB2, ZNF259, ZNF507, SWAP70, BCAS3 |
| Obesity | GWAS Catalog | ADCY3, AGBL4, ALDH2, ATP2A1, ATXN2L, BCDIN3D, BDNF, C5orf37, C6orf106, C9orf93, CADM1, CADM2, CBLN1, CDKAL1, CHST8, CUGBP1, DGKG, DMXL2, DNAJC27, ELAVL4, ERBB4, ETV5, FAIM2, FANCL, FHIT, FTO, FUBP1, GALNT10, GIPR, GNAT2, GNPDA2, GPRC5B, GRP, GTF3A, HHIP, HIF1AN, HIP1, HMGCR, HNF4G, HOXB5, IQCK, ITIH4, KAT8, KCNK3, KCNMA1, KCNQ1, KCTD15, KLF9, LBXCOR1, LEPR, LINGO2, LMX1B, LRP1B, LRRN6C, MAF, MAP2K5, MC4R, MTCH2, MTIF3, MYL2, NAV1, NDUFS3, NEGR1, NFE2L3, NLRC3, NRXN3, NT5C2, OLFM4, PACS1, PARK2, PCSK1, PGPEP1, PMAIP1, POC5, POMC, PRKCH, PRKD1, PTBP2, QPCTL, RABEP2, RARB, RASA2, RBJ, RPL27A, RPTOR, SBK1, SCARB2, SEC16B, SFRS10, SH2B1, SLC39A8, STXBP6, SULT1A2, TCF7L2, TFAP2B, TLR4, TMEM160, TMEM18, TNNI3K, TOMM40, TRIM66, TUB, TUFM, UBE2E3, USP37, ZC3H4, ZFP64, ZNF608 |
| Lipid | GWAS Catalog | A2ML1, ABCA1, ABCA6, ABCA8, ABCB11, ABCG5, ABCG8, ABO, ACAD11, ADAMTS3, AFF1, AKR1C4, AKT1, ALOX5, ANGPTL1, ANGPTL3, ANGPTL4, ANGPTL8, ANKRD55, ANXA9, APOA1, APOA4, APOA5, APOB, APOC1, APOC2, APOC3, APOC4, APOE, APOH, ARL15, ASAP3, ATG7, BAZ1B, BCL7B, BRAP, BRCA2, BUD13, C12orf51, C3, C6orf106, C8orf35, CAPN3, CBLN3, CCDC92, CCR6, CD36, CELSR2, CEP68, CERS2, CETP, CILP2, CITED2, CMIP, COBLL1, CPS1, CSNK1G3, CSPG3, CTCF, CUBN, CYP26A1, CYP7A1, DAGLB, DHX38, DLG4, DLGAP2, DNAH11, DNAH17, DOCK6, DOCK7, DSCAML1, DUSP3, EHBP1, ERGIC3, EVI5, FADS1, FADS2, FADS3, FAM117B, FAM13A, FOLH1, FRK, FRMD5, FTO, FUT2, GALNT2, GCKR, GPAM, GPR146, GRINA, HAS1, HAVCR1, HBS1L, HERPUD1, HFE, HIST1H4C, HLA-C, HMGCR, HNF1A, HNF4A, HP, HPR, INSIG2, INSR, IRF2BP2, IRS1, JMJD1C, KANK2, KIAA0323, KLF14, KLHL8, LACTB, LCAT, LDLR, LDLRAP1, LILRA5, LILRB2, LIPC, LIPG, LPA, LPL, LRP1, LRP4, LRPAP1, MACF1, MADD, MAFB, MAP3K1, MARCH8, MC4R, MLXIPL, MMAB, MOSC1, MTHFD2L, MVK, MYL2, MYLIP, NAT2, NCAN, NIPSNAP3A, NIPSNAP3B, NLRC5, NPC1L1, NR0B2, NR1H3, NUP93, NYNRIN, OR4C46, OSBPL7, PABPC4, PBX4, PCSK9, PEPD, PGS1, PHC1, PIGV, PINX1, PLTP, PPARG, PPP1R3B, PRKAG3, PRMT8, PROX1, PSRC1, PVRL2, RAB3D, RAB3GAP1, RAF1, RANBP10, RBM5, RMI2, RPS3A, RSPO3, SBNO1, SCARB1, SETD2, SF4, SIK3, SLC12A3, SLC18A1, SLC39A8, SNRPC, SNX13, SNX5, SORT1, SOST, SOX17, SPC24, SPTLC3, SPTY2D1, ST3GAL4, STAB1, STARD3, TBL2, TIMD4, TM4SF5, TMEM57, TOMM20, TOMM40, TOP1, TRIB1, TRPS1, TSPAN16, TTC39B, TYW1B, UBASH3B, UBE2L3, UBR1, VEGFA, VIM, VLDLR, ZBTB42, ZNF259, ZNF648, ZNF664 |
| Glucose | GWAS Catalog | ABCB11, ABCC11, ADCY5, ADRA2A, ANK1, ARAP1, ATP11A, CCT3, CDKAL1, CDKN2A, CDKN2B, CRY2, DGKB, DPYSL5, FADS1, FN3K, FOXA2, G6PC2, G6PC3, GCK, GCKR, GLIS3, HBS1L, HFE, HK1, IGF1, KANK1, MADD, MRPL33, MTNR1B, MYB, MYO9B, PCSK1, PDK1, PDX1, PPP1R3B, PROX1, RAPGEF4, SIX2, SIX3, SLC2A2, SLC30A8, SPTA1, TCF7L2, TMEM195, TMEM79, TMPRSS6, TUBGCP3, VPS13C |
| Insulin | GWAS Catalog | ARAP1, C2CD4A, C2CD4B, CHL1, COBLL1, GCKR, GRB14, HTR1A, IGF1, IRS1, LARP6, MADD, PCSK1, PDGFC, PPP1R3B, SGSM2, SLC30A8, TCF7L2, TUBA3C, VPS13C |
| T2D | GWAS Catalog | AP3S2, ARF5, ARL15, BCL11A, C2CD4A, C2CD4B, CAMK1D, CDC123, CDKAL1, CDKN2A, CDKN2B, CENTD2, DUSP9, FAF1, FAM58A, FITM2, FTO, GCC1, GLIS3, GPSM1, GRK5, HHEX, HLA-B, HMG20A, HMGA2, HNF1B, HNF4A, IDE, IGF2BP2, IRS1, JAZF1, KCNJ11, KCNQ1, KLF14, LAMA1, LEP, LGR5, LPP, MAEA, MTNR1B, PAX4, POU5F1, PPARG, PRC1, PSMD6, PTPRD, R3HDML, RASGRP1, RBM43, RND3, RREB1, SLC16A11, SLC16A13, SLC30A8, SND1, SPRY2, SRR, SSR1, TCF19, TCF7L2, THADA, TMEM154, TP53INP1, TSPAN8, UBE2E2, WFS1, ZBED3, ZFAND3, ZFAND6, ZMIZ1 |
| CAD | DisGeNet | ABAT, ABCA1, ABCB1, ABCG5, ABI2, ABO, ACE, ACE2, ACOT2, ACTA2, ACTB, ACVR1B, ADAMTS1, ADD1, ADD3, ADIPOQ, ADM2, ADNP, ADORA1, ADORA3, ADRB1, ADRB2, ADRB3, AES, AGER, AGT, AGTR1, AGTR2, AHR, AIF1, AIMP1, AK1, AKAP1, AKR1B1, AKT1, AKT2, ALB, ALDH2, ALDH6A1, ALDOA, ALOX15, ALOX5, ALOX5AP, ANGPT1, ANGPT2, ANXA1, ANXA5, APAF1, APEX1, APLP2, APOA1, APOA4, APOA5, APOB, APOC1, APOC3, APOE, APOH, AQP3, AQP4, AQP5, AQP8, AREG, ARID1B, ARNTL, ASIC1, ATM, ATP1A1, AVP, AVPR1A, BACE1, BAD, BAX, BBC3, BCHE, BCL2, BCL2L1, BDKRB1, BDKRB2, BDNF, BECN1, BHLHE40, BIRC5, BMP6, BRAP, BTG2, C1QB, C3, C7, C8A, C8B, CACYBP, CALCA, CALR, CAMK2D, CANX, CAPN10, CASP12, CASP3, CASP6, CASP9, CASR, CAST, CAT, CAV1, CAV3, CBS, CCL11, CCL2, CCL20, CCL3, CCL4, CCL5, CCNA2, CCND1, CCND2, CCR3, CCR4, CCR5, CD14, CD36, CD38, CD40, CD40LG, CD59, CD68, CDH2, CDK5, CDKN1B, CDKN2A, CDKN2B, CEBPB, CEBPD, CELSR2, CES3, CETP, CFH, CFTR, CHRM1, CIRBP, CITED2, CKB, CKM, CLEC10A, CLU, CNR2, COX5B, CP, CPB2, CPT1B, CREB1, CREM, CRH, CRHR2, CRK, CRP, CRYAB, CSF2, CSF2RB, CSPG4, CST3, CTGF, CTSB, CTSC, CTSL, CTTNBP2, CX3CL1, CX3CR1, CXCL10, CXCL12, CXCL2, CXCL8, CXCR3, CYBA, CYBB, CYCS, CYP27A1, CYP2C19, CYP2C9, CYP2D6, CYP2J2, CYP4F2, DAB2, DAB2IP, DBH, DCN, DDIT3, DGKZ, DIABLO, DLAT, DNM1L, DPYSL2, DRD2, DUSP1, DUSP6, DVL1, DYNLL1, EDN1, EDN2, EDNRA, EDNRB, EGR1, EGR2, EIF2A, EIF2AK3, EIF4E, EIF4EBP1, EIF4G1, ELN, ENDOG, ENSA, EPAS1, EPO, EPOR, ESR1, ESR2, F13A1, F13B, F2, F2R, F2RL1, F3, F5, F7, F8, FABP2, FABP5, FAS, FASLG, FEV, FEZ2, FGA, FGB, FGF1, FGF2, FGG, FLT1, FN1, FXYD1, FZD2, G6PD, GAL, GATA4, GATM, GBP2, GC, GCH1, GCKR, GCLC, GCLM, GDF15, GFRA1, GGT1, GH1, GHR, GHRH, GJA1, GJA4, GK, GLUD1, GNA12, GNB3, GP1BA, GPX1, GSK3B, GSR, GSTA1, GSTM1, GSTM2, GSTP1, GYG1, HADH, HADHA, HFE, HGF, HIF1A, HIVEP2, HK1, HK2, HLA-E, HMGB1, HMGCR, HMGCS2, HMOX1, HP, HRH1, HSD11B2, HSPA1A, HSPA1B, HSPA2, HSPA5, HSPA8, HSPA9, HSPD1, HTR1B, ICAM1, ID1, ID3, IFNA2, IFNG, IFNGR1, IGF1, IGF1R, IGF2, IGFBP3, IL10, IL10RB, IL12RB1, IL17A, IL17RA, IL18, IL1A, IL1B, IL1RAPL1, IL1RN, IL4R, IL6, IL6R, IL6ST, INS, IRAK1, IRAK4, IRF1, IRS1, IRX4, ITGA2, ITGA2B, ITGA3, ITGAV, ITGB3, JAK2, JUN, JUNB, KCNJ2, KCNJ8, KCNK10, KCNK2, KDR, KIF6, KITLG, KLF4, KLK1, LCAT, LDHA, LDLR, LGALS2, LGALS3, LIF, LIPC, LONP1, LPA, LPL, LRP8, LTA, LTA4H, MAP1LC3B, MAP2, MAP2K1, MAP2K3, MAP3K5, MAP3K8, MAPK1, MAPK14, MAPK3, MAPK8, MAPK9, MAPRE1, MAPT, MB, MBL2, MDH2, MDK, MEF2A, MEF2C, MEOX2, MEPE, MIA3, MIF, MMP1, MMP13, MMP14, MMP2, MMP3, MMP9, MPO, MSI1, MSN, MTHFR, MTOR, MUC13, MUC2, MX1, MYH7, NAIP, NCL, NEDD4, NES, NFE2L2, NFKB1, NFKBIA, NOS1, NOS2, NOS3, NPC1, NPPA, NPPB, NPTN, NPTXR, NPY, NR3C1, NR3C2, NR4A1, NRF1, NRIP1, OASL, OLR1, OPRD1, OPRK1, OPRM1, OXT, P2RY12, P4HB, PAK1, PAPPA, PARP1, PCNA, PCSK7, PCSK9, PDE3A, PDE4B, PDE4D, PDE5A, PDGFD, PDHA1, PDK4, PDLIM5, PEBP1, PENK, PFKFB3, PFKM, PGF, PGM1, PHACTR1, PLA2G2A, PLA2G4A, PLA2G7, PLAT, PLAU, PLAUR, PLCB1, PLCB3, PLCD1, PLCL2, PLN, PLTP, POMC, PON1, PON2, POSTN, PPARA, PPARG, PPARGC1A, PPAT, PPM1B, PPP1R15A, PPP2CA, PPP3R1, PRDX6, PRKACA, PRKACB, PRKCA, PRKCD, PRKCE, PROCR, PSMA6, PSMB5, PTGES, PTGIS, PTGS2, PTK2, PTK2B, PTN, PTPRA, PTX3, PYGB, PYGM, RAB12, RAB1B, RAB5A, RAB7A, RAD50, RALBP1, RAMP2, RANBP1, RB1, REG1A, RELA, REN, RET, RETN, RGCC, RHOC, RLN3, RNF214, ROS1, RPL17, RPS6KB1, RSAD2, RTN1, RXRG, RYR2, S100A4, S100B, SCARB1, SCN5A, SDC1, SELE, SELL, SELP, SERPINE1, SERPINH1, SF1, SFRP2, SFRP4, SH2B3, SIRT1, SIRT3, SLC11A2, SLC12A2, SLC17A4, SLC27A1, SLC27A6, SLC29A1, SLC6A1, SLC6A4, SLC6A6, SLC8A1, SLC8A2, SLC8A3, SLC9A1, SLPI, SMARCA4, SNAP23, SOCS1, SOCS3, SOD1, SOD2, SOD3, SORT1, SPARC, SRC, STAT3, STAT5A, SULT1A1, TAGLN, TAP1, TBX3, TBX5, TCF7L2, TEK, TFRC, TGFB1, TGFB2, TGFBR2, TH, THBD, THBS1, THBS4, TIMP2, TJP1, TLE4, TLR2, TLR4, TM6SF2, TMED2, TMPO, TNC, TNF, TNFRSF12A, TNFRSF14, TNFRSF1A, TNFRSF1B, TNFSF4, TNNI3, TNNT2, TOMM40, TP53, TRPV1, TTN, TTPA, TTR, TUBA1A, TXNIP, TXNRD1, UCP2, UGCG, UQCRFS1, USF1, UTS2, UTS2R, VCAM1, VCAN, VDAC2, VEGFA, VIP, VKORC1, VWF, WDR12, XDH, YBX1, YWHAB, ZEB1, ZFP36 |
| Obesity | DisGeNet | AACS, ABO, ACACB, ACADM, ACE, ACKR1, ACKR2, ACLY, ACMSD, ACP1, ACP5, ACSL1, ADCY9, ADH1B, ADIPOQ, ADIPOR1, ADIPOR2, ADRA2A, ADRB1, ADRB2, ADRB3, AFF4, AGMO, AGRP, AGT, AKAP1, AKR1C3, AKT1, AKT2, ALDH1L1, ALDH6A1, ALMS1, ALOX12, ALPK1, ANGPTL6, ANKRD26, ANO10, AOC3, APLN, APOA1, APOA5, APOB, APOBEC1, APOC3, APOE, AQP7, AR, ARHGAP11A, ARHGEF3, ASIP, ATAD2B, ATPAF1, AUTS2, BAD, BBS1, BBS4, BCHE, BCL2, BDNF, BMP2, C16orf96, C2CD4C, C3, C5AR1, C8orf34, CA3, CACNA2D1, CACNB2, CADM2, CAMK1D, CAMK2A, CARTPT, CASP1, CAV1, CAV2, CCDC33, CCDC77, CCKAR, CCL2, CCL4, CCL5, CCR3, CD36, CD40, CDH4, CDKAL1, CDKN1B, CDKN2A, CEP72, CES1, CETP, CFD, CHL1, CHRNA3, CIDEA, CLEC16A, CLOCK, CNR1, COL4A1, COMT, COX7C, CPB2, CPE, CPS1, CPT1A, CRH, CRHBP, CRP, CS, CSMD1, CST3, CTF1, CTNNBL1, CTSC, CTSS, CYB5A, CYCS, CYP19A1, CYP26B1, CYP2E1, DAPL1, DCC, DCN, DCXR, DEFB1, DFNB31, DPYD, DRD1, DRD2, DRD4, DSCAM, DYNC1I1, ECHS1, EFNB1, EML6, ENPP1, EP300, ERBB3, ESR1, ESR2, ETFDH, ETV5, F2, F5, FAAH, FABP2, FADS1, FAIM2, FAM19A2, FARS2, FASN, FBXL7, FGF21, FGFR1, FOXO3, FTL, FTO, G6PD, GABRA6, GAD2, GALP, GAS7, GCG, GCH1, GCK, GFPT1, GH1, GHRL, GHSR, GIP, GIPR, GJA5, GLP1R, GLRX, GLUL, GNAS, GNAT2, GNB3, GNPDA2, GP2, GPAM, GPC5, GPC6, GPR12, GPSM3, GPX1, GPX3, GREB1, GSTM1, HADH, HCRT, HHEX, HK1, HK2, HMGB2, HMGCLL1, HMGCS1, HMOX1, HOXB3, HOXB5, HP, HRC, HRH3, HS6ST3, HSD11B1, HSD11B2, HTR1B, HTR2A, HTR2C, ICA1, ICAM1, IFI16, IFNG, IGF1, IGF2, IGF2BP2, IGFBP2, IGFBP3, IGSF9B, IL10, IL18, IL1B, IL6, IL6R, INADL, INPP5E, INS, INSIG2, INSR, IRS1, IRS2, ITPR1, ITPR3, JAK2, KCNJ11, KCNMA1, KCTD15, KDM3A, KITLG, KLF9, KLKB1, KMO, LACTB, LBP, LCN2, LDLR, LEP, LEPR, LHFPL3, LINGO2, LIPC, LIPE, LPL, MAP2K5, MATK, MC3R, MC4R, MCHR1, MDGA2, ME1, MKKS, MMP9, MOXD1, MPRIP, MTCH2, MTHFR, MTNR1B, MUT, MVB12B, MYC, MYOD1, MYOG, NAMPT, NAP1L1, NBEAL1, NCAM2, NCF2, NCOA1, NEGR1, NEIL1, NHLH2, NINJ1, NMB, NMNAT2, NMU, NOS1, NOS3, NOTCH4, NPC1, NPPB, NPY, NPY1R, NPY2R, NPY5R, NQO1, NR0B2, NR1I2, NR1I3, NR3C1, NRXN3, NTRK2, NUCB2, OTC, OXCT1, PACS1, PAK6, PARP1, PAX5, PCDH9, PCK1, PCSK1, PCSK1N, PCSK2, PFKFB3, PIK3R1, PLEKHS1, PLIN1, PLSCR3, PMCH, PNLIP, PNPLA3, POC5, POMC, PON1, PON2, PPARA, PPARD, PPARG, PPARGC1A, PPARGC1B, PPM1L, PPP1R12B, PRKAR2B, PRKCB, PRKCD, PRKCH, PRKCI, PRKG1, PRLH, PRLHR, PRM1, PROX1, PTGS2, PTPN1, PTPRF, PYROXD2, PYY, RABEP2, RABL3, RAI1, RASGEF1A, RBP4, RETN, RHOA, RICTOR, RPS6KB1, RPTOR, RSC1A1, RSU1, SAT1, SCD, SCNN1A, SDC1, SDC3, SDCCAG8, SEC16B, SEMA5A, SERPINA12, SERPINE1, SERPINF1, SFTPA1, SFTPB, SGCZ, SH2B1, SHANK2, SIM1, SIPA1L1, SIRT1, SIRT3, SIRT6, SLC16A7, SLC22A1, SLC22A23, SLC27A1, SLC29A3, SLC4A1, SLC6A4, SLC8A1, SLC9A3, SOCS1, SOCS3, SOD1, SOD2, SPOCK3, SPTB, SRD5A1, SREBF1, SREBF2, STAT3, STAT4, STK11, STS, SUCLA2, SUCLG2, TBC1D1, TBCE, TCF7L2, TENM4, TF, TFAP2B, TFRC, TGFB1, TLR4, TMEM18, TMEM229B, TNF, TNFRSF1B, TRIM66, TRPV1, TSEN34, TTC28, TUB, TUBGCP6, TWIST1, TYK2, UCP1, UCP2, UCP3, UGT2B7, UMODL1, UQCRC2, VDR, VEGFA, VLDLR, WDPCP, WWOX, ZNF446, ZPR1, ZZZ3 |
| Lipid | DisGeNet | ABCA1, ABCB1, ABCG5, ABCG8, ACE, ADIPOQ, ADRB2, ADRB3, AGT, AGTR1, AHR, ALB, ALPL, ANGPTL4, APC, APOA1, APOA2, APOA4, APOA5, APOB, APOBEC1, APOC3, APOE, ATP7B, CD40, CES1, CETP, COL3A1, CRP, CSF1, CTF1, CYP7A1, DNAH11, EDN1, EEF1A2, EPHX2, F7, G6PD, GCG, GFPT1, GHR, GNB3, GSR, HMGCR, HP, HSD11B1, ICAM1, IRS1, KL, LCAT, LDLR, LDLRAP1, LEP, LEPR, LIPC, LMX1B, LPA, LPL, MIF, MTHFR, MTTP, MYLK, NCF1, NEIL1, NOS3, NOX1, NR4A3, PCSK9, PLTP, PON1, PON2, PPARA, PPARG, PPARGC1B, RGN, SCAP, SDC1, SELE, SERPINF2, SHC1, SLC27A1, SMARCD1, SMEK2, SREBF1, SREBF2, STAP1, TGFB1, TLR2, TMEM57, TNF, VCAM1, VNN1 |
| Glucose | DisGeNet | ADA, ADIPOR1, ADRB1, ADRBK1, AGER, AKR1B1, AVP, CNTF, COL3A1, CYP11A1, DRD1, EPAS1, FBN1, GCG, GCK, GIPR, GLP1R, GPX1, HCRT, HCRTR2, HMGA1, HRH3, HSD11B1, HSPD1, HTR2A, ICAM1, IL1B, INS, INSR, IRS2, KCNJ11, LDHA, LEPR, MMP9, NFE2L2, NOS3, NQO1, PCSK1, PDX1, PKLR, PLA2G4A, PPARGC1A, PRDX4, PRKCB, PRKCZ, PRNP, PTGS2, SIM1, SP1, TCF7L2, TNF |
| Insulin | DisGeNet | ACACA, ACACB, ACE, ADIPOQ, ADORA1, ADRB2, ADRB3, AGTR1, AGTR2, AHR, APLN, APOC3, AR, ARG1, ATM, BAD, BDKRB1, C3, CASP1, CD36, CNR1, CNTF, CPE, CTF1, DRD1, EGFR, ENPP1, FABP2, FABP4, FOXO1, FRK, FTO, G6PD, GCK, GH1, GNAS, GPX3, HMGA1, HMOX1, HSD11B1, HSPD1, IGFALS, IGFBP2, IKBKB, IL6, INPPL1, INS, INSR, IRS1, KCNJ11, KCNMB1, LEP, LEPR, LIPC, LPL, MC4R, NEIL1, NOS2, NOS3, NR4A1, NR4A3, PDE3A, PDX1, PIK3R1, PLTP, PMCH, PPARA, PPARG, PPARGC1A, PRDX4, PRKAA1, PRKAA2, PRKCB, PRKCD, PRKCQ, PTEN, RARRES2, RELA, RETN, SCD, SEPP1, SERPINE1, SERPINF1, SI, SIRT1, SIRT4, SLC12A2, SLC27A1, SLC2A4, SOD2, SREBF1, SREBF2, STK11, STS, TCF7L2, TF, TNF, TRIB3 |
| T2D | DisGeNet | AANAT, ABCA1, ABCC2, ABCC8, ACACA, ACACB, ACE, ACOT2, ACP1, ADAMTS9, ADCY5, ADD1, ADIPOQ, ADIPOR1, ADIPOR2, ADORA1, ADRA2A, ADRA2B, ADRB2, ADRB3, AGER, AGRP, AGT, AGTR1, AGTR2, AHR, AK1, AKR1B1, AKT1, AKT2, ALB, ALDOB, ANK1, ANKRD23, AOC3, AP3S2, APLN, APOA1, APOA4, APOA5, APOB, APOC3, APOE, AR, ARAP1, ARG1, ARL15, ARX, ASIP, ATF3, ATM, ATP2A2, ATP2A3, AVP, BAD, BAX, BCL11A, BCL2, BCL2L1, BDKRB1, BDNF, BHMT, C2CD4A, C2CD4B, C3, CALCA, CALM3, CAMK1D, CAPN10, CARTPT, CASP1, CASP12, CASP3, CASP7, CASP8, CASQ1, CAT, CBS, CCKAR, CCL2, CCND2, CCR5, CD36, CD38, CDC123, CDKAL1, CDKN2A, CDKN2B, CDO1, CETP, CMIP, CNR1, CNTF, CPE, CPT1A, CRHR1, CRP, CRTC2, CTF1, CTGF, CTLA4, CYB5R4, CYBA, CYP11A1, CYP1A2, CYP2C19, CYP2C9, CYP2D6, CYP2E1, CYP3A4, DCD, DEFB1, DGKB, DNER, DNMT1, DRD1, ECE1, EDN1, EDNRA, EDNRB, EGFR, ELMO1, ENPP1, EPAS1, ESR1, EXT2, F3, F7, FABP2, FABP4, FAF1, FAM58A, FAS, FEM1B, FOXM1, FOXO1, FRK, FTO, G6PC, G6PC2, G6PD, GABRA4, GADD45GIP1, GCG, GCGR, GCK, GCKR, GH1, GHRL, GIP, GIPR, GJA1, GLIS3, GNAS, GNB3, GPD2, GPSM1, GPX1, GPX3, GRB14, GRK5, GSTM1, GYS1, HBA1, HFE, HHEX, HIF1A, HK1, HK2, HLA-A, HLA-DQA1, HLA-DQB1, HLA-DRB1, HMG20A, HMGA1, HMGA2, HMGCR, HMOX1, HNF1A, HNF1B, HNF4A, HP, HPX, HSD11B1, HSD17B3, HSPD1, HTR2C, IAPP, ICAM1, ID1, IDE, IGF1, IGF1R, IGF2, IGF2BP2, IGFALS, IGFBP2, IKBKB, IL10, IL1A, IL1B, IL1RN, IL4, IL6, IL6R, INPPL1, INS, INSR, INTS8, IRS1, IRS2, ITGA2, ITLN1, JAZF1, KCNJ11, KCNK16, KCNK17, KCNMB1, KCNQ1, KIF11, KL, KLF11, LAMA1, LDLR, LEP, LEPR, LGR5, LIPC, LMNA, LNPEP, LPL, LTA, MAEA, MAFA, MAPK8IP1, MAT1A, MC4R, MIF, MKI67, MMP1, MMP3, MMP9, MOK, MPHOSPH9, MPST, MTHFR, MTNR1A, MTNR1B, MTR, MTTP, NAMPT, NCOA6, NEIL1, NEUROD1, NEUROG3, NFE2L2, NFKB1, NKX6-1, NOG, NOS1, NOS1AP, NOS2, NOS3, NOTCH2, NPPA, NPPB, NPY, NR1D1, NR3C1, NR4A1, NR4A3, NUCB2, OASL, OGG1, OSBPL1A, OXCT1, PAM, PAX4, PAX6, PC, PCK1, PCNXL2, PCSK1, PCSK2, PDE3A, PDX1, PEPD, PHOX2A, PIK3R1, PLAT, PLN, PLTP, PMCH, PON1, PON2, POU5F1, PPARA, PPARD, PPARG, PPARGC1A, PPARGC1B, PPP1R3A, PPP1R3C, PPP2CA, PRC1, PRDX4, PRKAA1, PRKAA2, PRKCB, PRKCD, PRKCI, PRKCQ, PRKCZ, PROX1, PSMD6, PTEN, PTGDS, PTGES2, PTGS2, PTPN1, PTPN22, PTPRD, RARRES2, RASGRP1, RBMS1, RBP4, REG3A, RELA, RETN, RGS7, RNF6, S100A6, SCD, SCN1B, SELE, SEPP1, SERPINA12, SERPINE1, SERPINF1, SGCG, SH2B1, SHBG, SI, SIRT1, SIRT4, SLC12A2, SLC16A11, SLC16A13, SLC27A1, SLC2A1, SLC2A2, SLC2A4, SLC30A8, SLC9A1, SNAP25, SOD1, SOD2, SRBD1, SRC, SRD5A1, SREBF1, SREBF2, SRR, ST6GAL1, STAR, STK11, STS, SUMO4, TCF20, TCF7, TCF7L2, TF, TGFB1, TGFBR2, THADA, THBD, TIMP1, TLR4, TNF, TNFRSF1A, TNFRSF1B, TP53INP1, TRIB3, TRPC1, TRPC6, TSPAN8, UBE2E2, UCP1, UCP2, UCP3, USF1, UTS2, VCAM1, VDR, VEGFA, VPS26A, VPS33B, VWF, WFS1, WRN, ZFAND3, ZMIZ1 |
