# Supplementary material for: Shared genetic regulatory networks for cardiovascular disease and type 2 diabetes in multiple populations of diverse ethnicities in the United States
Source: PLoS Genet. 2017 Sep 28;13(9):e1007040. doi: 10.1371/journal.pgen.1007040 (PMC5634657; doi:10.1371/journal.pgen.1007040)
Supplement: S4 Table — (DOCX) [file pgen.1007040.s012.docx]

**S4 Table.** Summary information of genome-wide association studies

| **Study** | WHI-GARNET | WHI-SHARe | WHI-SHARe | Jackson Heart Study | Framingham Heart Study | CARDIoGRAM  plusC4D | DIAGRAM |
| --- | --- | --- | --- | --- | --- | --- | --- |
| **Origin** | Caucasian | African American | Hispanic  American | African American | Caucasian | Mostly Caucasian | Caucasian and Asian |
| **Imputation** | Hapmap3 | Hapmap3 | Hapmap3 | 1000G | 1000G | 1000G | Hapmap3 |
| **CVD Cases/Controls** | 545/2130 | 483/5880 | 131/2893 | 240/1908 | 471/5443 | 60801/123504 | - |
| **T2D Cases/Controls** | 1022/2130 | 1381/5739 | 581/2681 | 683/847 | 287/4657 | - | 26488/83964 |
| **Platform** | Illuminia | Affymetrix | Affymetrix | Affymetrix | Affymetrix | - | - |
| **N of SNPs** | 868609 | 836045 | 836060 | 8041765 | 5668200 | 6104813 | 2915012 |
