# Supplementary material for: Shared genetic regulatory networks for cardiovascular disease and type 2 diabetes in multiple populations of diverse ethnicities in the United States
Source: PLoS Genet. 2017 Sep 28;13(9):e1007040. doi: 10.1371/journal.pgen.1007040 (PMC5634657; doi:10.1371/journal.pgen.1007040)
Supplement: S5 Table — (DOCX) [file pgen.1007040.s013.docx]

**S5 Table.** Data resources and references for co-expression networks

| **Tissue** | **Species** | **Dataset** |
| --- | --- | --- |
| Aortic endothelial cells | Human | 147 heart transplant donors [[14](#_ENREF_14)] |
| Adipose tissue | Human | 1,675 individuals from two Icelandic cohorts [[15](#_ENREF_15)] |
|  | Human | 1,008 obese patients [[16](#_ENREF_16)] |
|  | Mouse | C57BL/6J x A/J mouse cross[[17](#_ENREF_17)] |
|  | Mouse | C57BL/6J x C3H ApoE -/- mouse cross [[18](#_ENREF_18), [19](#_ENREF_19)] |
|  | Mouse | C57BL/6J x C3H wildtype mouse cross [[20](#_ENREF_20)] |
|  | Mouse | C57BL/6J x BTBR Lepob mouse cross [[21](#_ENREF_21)] |
| Blood | Human | 1,675 individuals from two Icelandic cohorts [[15](#_ENREF_15)] |
|  | Human | 986 T2D patients and 5004 controls [[22](#_ENREF_22)] |
| Heart | Mouse | C57BL/6J x A/J mouse cross [[17](#_ENREF_17)] |
| Hypothalamus | Mouse | C57BL/6J x A/J mouse cross [[17](#_ENREF_17)] |
|  | Mouse | C57BL/6J x C3H ApoE -/- mouse cross [[18](#_ENREF_18), [19](#_ENREF_19)] |
|  | Mouse | C57BL/6J x BTBR Lepob mouse cross [[21](#_ENREF_21)] |
| Islet cells | Mouse | C57BL/6J x BTBR Lepob mouse cross [[21](#_ENREF_21)] |
| Kidney | Mouse | C57BL/6J x A/J mouse cross [[17](#_ENREF_17)] |
| Liver | Human | 427 individuals [[20](#_ENREF_20)] |
|  | Human | 1,008 obese patients [[16](#_ENREF_16)] |
|  | Mouse | C57BL/6J x A/J mouse cross [[17](#_ENREF_17)] |
|  | Mouse | C57BL/6J x C3H ApoE -/- mouse cross [[18](#_ENREF_18), [19](#_ENREF_19)] |
|  | Mouse | C57BL/6J x C3H wildtype mouse cross [[20](#_ENREF_20)] |
|  | Mouse | C57BL/6J x BTBR Lepob mouse cross [[21](#_ENREF_21)] |
| Muscle | Mouse | C57BL/6J x A/J mouse cross [[17](#_ENREF_17)] |
|  | Mouse | C57BL/6J x C3H ApoE -/- mouse cross [[18](#_ENREF_18), [19](#_ENREF_19)] |
|  | Mouse | C57BL/6J x C3H wildtype mouse cross [[20](#_ENREF_20)] |
|  | Mouse | C57BL/6J x BTBR Lepob mouse cross [[21](#_ENREF_21)] |
