# Supplementary material for: Shared genetic regulatory networks for cardiovascular disease and type 2 diabetes in multiple populations of diverse ethnicities in the United States
Source: PLoS Genet. 2017 Sep 28;13(9):e1007040. doi: 10.1371/journal.pgen.1007040 (PMC5634657; doi:10.1371/journal.pgen.1007040)
Supplement: S6 Table — (DOCX) [file pgen.1007040.s014.docx]

**S6 Table.** Data resources and references for expression QTLs

| **Tissue** | **Dataset** |
| --- | --- |
| Adipose | 449 human donors [[23](#_ENREF_23)] |
|  | 1,675 individuals from two Icelandic cohorts [[15](#_ENREF_15)] |
|  | 1,008 obese patients [[16](#_ENREF_16)] |
|  | 150 female twins [[24](#_ENREF_24)] |
| Adrenal gland | 449 human donors [[23](#_ENREF_23)] |
| Aorta | 449 human donors [[23](#_ENREF_23)] |
| Blood | 449 human donors [[23](#_ENREF_23)] |
|  | 1,675 individuals from two Icelandic cohorts [[15](#_ENREF_15)] |
|  | 1,469 unrelated individuals [[25](#_ENREF_25)] |
| Brain | 449 human donors [[23](#_ENREF_23)] |
| Colon | 449 human donors [[23](#_ENREF_23)] |
| Coronary artery | 449 human donors [[23](#_ENREF_23)] |
| Esophagus | 449 human donors [[23](#_ENREF_23)] |
| Hypothalamus | 449 human donors [[23](#_ENREF_23)] |
| Ileum | 449 human donors [[23](#_ENREF_23)] |
| Liver | 449 human donors [[23](#_ENREF_23)] |
|  | 427 individuals [[20](#_ENREF_20)] |
|  | 1,008 obese patients [[16](#_ENREF_16)] |
| Lymphocyte | 449 human donors [[23](#_ENREF_23)] |
|  | Umbilical cords of 85 Western European individuals [[26](#_ENREF_26)] |
|  | 400 children of families with a proband with asthma [[27](#_ENREF_27)] |
|  | 60 HapMap participants of European descent [[28](#_ENREF_28)] |
|  | 270 HapMap participants [[29](#_ENREF_29)] |
|  | 726 HapMap3 participants [[30](#_ENREF_30)] |
|  | 30 European and 30 Yoruba HapMap participants [[31](#_ENREF_31)] |
|  | 150 female twins [[24](#_ENREF_24)] |
| Macrophage | 758 individuals [[32](#_ENREF_32)] |
| Monocyte | 758 individuals [[32](#_ENREF_32)] |
| Skeletal muscle | 449 human donors [[23](#_ENREF_23)] |
| Pancreas | 449 human donors [[23](#_ENREF_23)] |
| Stomach | 449 human donors [[23](#_ENREF_23)] |
| Thyroid | 449 human donors [[23](#_ENREF_23)] |
| Vascular endothelium | 449 human donors [[23](#_ENREF_23)] |
|  | 147 heart transplant donors [[14](#_ENREF_14)] |
