# Supplementary material for: Shared genetic regulatory networks for cardiovascular disease and type 2 diabetes in multiple populations of diverse ethnicities in the United States
Source: PLoS Genet. 2017 Sep 28;13(9):e1007040. doi: 10.1371/journal.pgen.1007040 (PMC5634657; doi:10.1371/journal.pgen.1007040)
Supplement: S7 Table — (DOCX) [file pgen.1007040.s015.docx]

**S7 Table.** Data resources and references for gene-gene regulatory networks (GIANT and Bayesian networks)

| **Tissue** | **Source*** | **Dataset** |
| --- | --- | --- |
| Adipose | GIANT | Integrated 987 genome-scale data sets |
|  | Bayesian networks | 1,675 individuals from two Icelandic cohorts [[15](#_ENREF_15)] |
|  |  | C57BL/6J x A/J mouse cross [[17](#_ENREF_17)] |
|  |  | C57BL/6J x C3H ApoE -/- mouse cross [[18](#_ENREF_18), [19](#_ENREF_19)] |
|  |  | C57BL/6J x C3H wildtype mouse cross [[20](#_ENREF_20)] |
|  |  | C57BL/6J x BTBR Lepob mouse cross [[21](#_ENREF_21)] |
| Adrenal cortex | GIANT | Integrated 987 genome-scale data sets |
| Adrenal gland | GIANT | Integrated 987 genome-scale data sets |
| Aorta | GIANT | Integrated 987 genome-scale data sets |
| Artery | GIANT | Integrated 987 genome-scale data sets |
| Blood | GIANT | Integrated 987 genome-scale data sets |
|  | Bayesian networks | 1,675 individuals from two Icelandic cohorts [[15](#_ENREF_15)] |
| Colon | GIANT | Integrated 987 genome-scale data sets |
| Esophagus | GIANT | Integrated 987 genome-scale data sets |
| Hepatocyte | GIANT | Integrated 987 genome-scale data sets |
| Hypothalamus | GIANT | Integrated 987 genome-scale data sets |
|  | Bayesian networks | C57BL/6J x A/J mouse cross [[17](#_ENREF_17)] |
|  |  | C57BL/6J x C3H ApoE -/- mouse cross [[18](#_ENREF_18), [19](#_ENREF_19)] |
|  |  | C57BL/6J x BTBR Lepob mouse cross [[21](#_ENREF_21)] |
| Ileum | GIANT | Integrated 987 genome-scale data sets |
| Intestine | GIANT | Integrated 987 genome-scale data sets |
| Islet | GIANT | Integrated 987 genome-scale data sets |
|  | Bayesian networks | C57BL/6J x BTBR Lepob mouse cross [[21](#_ENREF_21)] |
| Jejunum | GIANT | Integrated 987 genome-scale data sets |
| Liver | GIANT | Integrated 987 genome-scale data sets |
|  | Bayesian networks | 427 individuals [[20](#_ENREF_20)] |
|  |  | C57BL/6J x A/J mouse cross [[17](#_ENREF_17)] |
|  |  | C57BL/6J x C3H ApoE -/- mouse cross [[18](#_ENREF_18), [19](#_ENREF_19)] |
|  |  | C57BL/6J x C3H wildtype mouse cross [[20](#_ENREF_20)] |
|  |  | C57BL/6J x BTBR Lepob mouse cross [[21](#_ENREF_21)] |
| Pancreas | GIANT | Integrated 987 genome-scale data sets |
| Kidney | GIANT | Integrated 987 genome-scale data sets |
|  | Bayesian networks | C57BL/6J x A/J mouse cross [[17](#_ENREF_17)] |
| Lymphocyte | GIANT | Integrated 987 genome-scale data sets |
| Macrophage | GIANT | Integrated 987 genome-scale data sets |
| Monocyte | GIANT | Integrated 987 genome-scale data sets |
| Skeletal Muscle | GIANT | Integrated 987 genome-scale data sets |
|  | Bayesian networks | C57BL/6J x A/J mouse cross [[17](#_ENREF_17)] |
|  |  | C57BL/6J x C3H ApoE -/- mouse cross [[18](#_ENREF_18), [19](#_ENREF_19)] |
|  |  | C57BL/6J x C3H wildtype mouse cross [[20](#_ENREF_20)] |
|  |  | C57BL/6J x BTBR Lepob mouse cross [[21](#_ENREF_21)] |
| Small Intestine | GIANT | Integrated 987 genome-scale data sets |
| Stomach | GIANT | Integrated 987 genome-scale data sets |
| Thyroid | GIANT | Integrated 987 genome-scale data sets |
| Vascular endothelium | GIANT | Integrated 987 genome-scale data sets |

*Gene-gene regulatory networks are collected from the online database provided in the Genome-scale Integrated Analysis of gene Networks in Tissues (GIANT) [[11](#_ENREF_11)], as well as other indicated independent studies.
